# Supplementary material for: EMAeHealth, a digital tool for the self-management of women’s health needs during pregnancy, childbirth and the puerperium: protocol for a hybrid effectiveness-implementation study
Source: BMJ Open. 2022 Sep 1;12(9):e055031. doi: 10.1136/bmjopen-2021-055031 (PMC9438065; doi:10.1136/bmjopen-2021-055031)
Supplement: Supplementary data [file bmjopen-2021-055031supp001.pdf]

Patient information sheet for clinical trial and consent

Patient Information Sheet

For years we have been trying to improve the initiatives we carry out at Osakidetza to improve care for women during pregnancy and postpartum. We are therefore contacting you to ask for your consent to participate in a study your health center and midwife are collaborating in.

Invitation to participate and description of the procedure

Your health center and particularly your midwife are actively collaborating with the Bizkaia Primary Care Research Unit, part of the Biocruces-Bizkaia institute, to promote maternal education that is better adapted to the needs of women today. As a result, our research team is developing a website to support Maternal Education and we want to know how effective it is, so we would like to ask for your participation.

This website will offer rigorous information, selected by health professionals, on topics of interest throughout the period of pregnancy, childbirth and postpartum. It will enable women to access their clinical data securely, and will contain and offer resources at key moments in the maternity/paternity process. It will inform and support women in leading a healthy lifestyle, helping in the development of a birth plan that is adapted to each woman, advising on the appropriate choice at the onset of childbirth and offering care in the first days postpartum.

The website will be available to some randomly selected health centers, while others will not have this resource. We are doing this to compare the results on the health of women who use this resource (which you access with a password provided by your midwife) with those of women who do not have access to it.

Your participation in the study, regardless of your reference health center (with or without the website) will consist of filling in a series of sociodemographic questionnaires (e.g. age, level of studies, profession) and clinical questions (such as quality of life related to health or perception of self-efficacy during the birth process and breastfeeding), and it will also involve giving us permission to collect data from your medical records related to your pregnancy, childbirth and postpartum history.

The questionnaires must be completed at the beginning of pregnancy (approximately week 8), at weeks 24 and 34-36, and at weeks 1 and 6 postpartum. You fill it in online; your midwife will ask you for your mobile phone number and/or email address, and you will be sent a link to access the questionnaires. You will be able to complete them on a mobile phone, tablet or computer.

If you decide that you wish to participate, we will ask you to sign the attached consent form, which will be filed with the rest of the study documents.

Data processing and protection

The data that we collect via your mobile phone will be processed in an anonymized form, since it will be accessed with a numerical code, and confidentiality will be guaranteed at all times. The data collected for the study will be identified by this code, so that it does not include information that can identify you, and only your study doctor/researchers will be able to relate this data to you and your medical records. Therefore, your identity will not be disclosed to any person,

except in cases of medical emergency or legal requirement. The processing, communication and transfer of personal data of all participants will comply with the provisions of the law.

This study complies with the provisions of REGULATION (EU) 2016/679 OF THE EUROPEAN PARLIAMENT AND OF THE COUNCIL of 27 April 2016 on the protection of natural persons with regard to the processing of personal data and the free movement of such data, and of Organic Law 3/2018, of 5 December 2018 on the Protection of Personal Data and guarantee of digital rights that repeals Organic Law 15/1999 of 5 December 1999, on the protection of personal data. Personal data will be processed by the Basque Health Service, Osakidetza. No data will be transferred to third parties, except under legal obligation. The patient will be informed that they have the right of access, rectification, deletion of their data, and limitation or opposition to its use. In addition, further information on data protection can be found at the following website: http://www.osakidetza.euskadi.eus/protecciondatos

Benefits and risks

Participation in the study is entirely altruistic and carries no risk to you. The only cost that can be incurred is the time dedicated to answering the questions in the questionnaires. You are not required to answer questions that you consider to be private.

In return, you will be collaborating with research dedicated to the evaluation and improvement of the health service.

Voluntary participation

Your participation in the study is entirely voluntary.

Right to withdraw from the study

You have the right to withdraw from the study at any time without consequence to you.

Who to contact about the study

You can contact the Lead Researcher, Isabel Artieta, on tel. 946007711 if you need any clarification or want to resolve any doubts regarding the study.

The Coordinating Center for the study is the Bizkaia Primary Care Research Unit, which is part of Biocruces-Bizkaia and is located in Building 3, C/Plaza de Cruces, s/n. 48903 Barakaldo (Bizkaia). Tel. 946006637.

This study has been supervised and approved by the Basque Clinical Research Ethics Committee. (CEIm)

DECLARATION OF CONSENT

Study Title: "**Effectiveness and usability of a website for the self-management of women's health needs during pregnancy, childbirth and the postpartum period: EMAeHealth**"

Centre: **Bizkaia Primary Care Research Unit (Biocruces-Bizkaia Institute)**

I (name and surname) declare that

………

I have read the information sheet given to me.

I was able to ask questions about the study.

I have received enough information about the study.

I understand that in case of doubt I can go to the lead researcher:

Isabel Artieta. Zuazo Health Center (Barakaldo). Tel. 946007711

I understand that my participation is voluntary and that at any time, without explanation and without any impact on my medical care, I can withdraw from the study.

I freely give my consent to participate in the study.

DATE: PARTICIPANT'S SIGNATURE

The data collected will be added to a computer database in anonymized form, and be used to evaluate the research. This study complies with the provisions of REGULATION (EU) 2016/679 OF THE EUROPEAN PARLIAMENT AND OF THE COUNCIL of 27 April 2016 on the protection of natural persons with regard to the processing of personal data and the free movement of such data, and of Organic Law 3/2018, of 5 December 2018 on the Protection of Personal Data and guarantee of digital rights that repeals Organic Law 15/1999 of 5 December 1999, on the protection of personal data. Personal data will be processed by the Basque Health Service, Osakidetza. No data will be transferred to third parties, except under legal obligation. The patient will be informed that they have the right of access, rectification, deletion of their data, and limitation or opposition to its use. In addition, further information on data protection can be found at the following website: http://www.osakidetza.euskadi.eus/protecciondatos

THIS STUDY HAS BEEN EVALUATED AND APPROVED BY THE BASQUE CLINICAL RESEARCH ETHICS COMMITTEE (CEIm)

Focus groups of patients and focus groups of professionals and consent

Patient Information Sheet

In Osakidetza, we are trying to improve care for women during pregnancy and postpartum, and we would like your participation in a focus group in order to **get your opinion about the EMAeHealth website** that we have developed.

Invitation to participate in a focus group and description of the procedure

Your health center and particularly your midwife, together with researchers from the Bizkaia Primary Care Research Unit (Biocruces-Bizkaia), are looking into how to promote care that is better adapted to the needs of women today. To achieve this goal, as you know, a website called EMAeHealth has been designed.

As we would like to get a better idea of the opinions of the users about this new resource for Maternal Education - in terms of its ease of use, appropriateness and effectiveness - we would like to invite you to attend a focus group to explore these issues in depth. This participation is voluntary and only means attending a focus group meeting once. This meeting will be held on xxxxx (date), at xxxx (address) and will last approximately 90 minutes. The discussion will be moderated by two researchers from the Research Unit (xx and xx) and will be attended by other web users (between 6 and 8 of them) from different health centers, to talk about the topics under discussion.

Data processing and protection

The conversation generated by the group will be recorded entirely in audio to facilitate its subsequent transcription and analysis, with the collaboration of the qualitative studies research company xxx, which complies with the relevant legislation on data protection and is based in the Basque Country. We assure you that your opinions will be anonymized and that the confidentiality of the data collected will be guaranteed at all times. The audio files of the recordings and their corresponding transcripts (with names previously removed) will be stored in the computers of the Research Unit, access to which is restricted to researchers and

password-protected. Before the start of the recording, each participant will sign a consent form to authorize their participation in the study.

This study complies with the provisions of REGULATION (EU) 2016/679 OF THE EUROPEAN PARLIAMENT AND OF THE COUNCIL of 27 April 2016 on the protection of natural persons with regard to the processing of personal data and the free movement of such data, and of Organic Law 3/2018, of 5 December 2018 on the Protection of Personal Data and guarantee of digital rights that repeals Organic Law 15/1999 of 5 December 1999, on the protection of personal data. Personal data will be processed by the Basque Health Service, Osakidetza. No data will be transferred to third parties, except under legal obligation. The patient will be informed that they have the right of access, rectification, deletion of their data, and limitation or opposition to its use. In addition, further information on data protection can be found at the following website: http://www.osakidetza.euskadi.eus/protecciondatos

Benefits and risks

During the focus group discussion, the moderators will explain in detail what the technique involves, and you will be able to ask any questions or raise doubts throughout the entire session to make sure that you feel comfortable in that situation. You will also be given all kinds of opportunities to express your opinions freely and voluntarily, guaranteeing you an atmosphere of respect and confidentiality at all times. The main benefit of this participation is sharing your experience about the website, reflecting on ways to improve it and adapt it to the objective of promoting Maternal Education to meet your needs, thus contributing to the improvement of our health system.

Moreover, the moderators will take measures to minimize the impact of any potential risk that you or other participants may perceive or feel during the discussion in the group (such as fatigue, discomfort with any questions or conflict between participants). For example, you can take breaks, remain silent, state that you wish not to continue in the study, request more information about the objectives and procedures of the study and so on.

We remain at your entire disposal to offer any clarifications and answer your questions. If you need more details, you can contact the lead researcher of the study or the Bizkaia Primary Care Research Unit:

- **Isabel Artieta. Zuazo Health Center (Barakaldo). Tel. 946007711**
- **Bizkaia Primary Care Research Unit (Biocruces-Bizkaia). Tel. 946006637.** (Maite Espinosa)

Healthcare Professional Information Sheet

For years we have been involved in improving the initiatives we carry out in Osakidetza to improve care for women during pregnancy and postpartum. Therefore, we are contacting you to request your participation in a focus group, in order to **get your opinion about the EMAeHealth website** that we are developing.

Invitation to participate in a focus group and description of the procedure

We work with the Bizkaia Primary Care Research Unit (UIAPB) - Biocruces Bizkaia, in the development of strategies to promote Maternal Education that is better adapted to the needs of women today. To achieve this goal, as you know a website, EMAeHealth, has been designed, which we intend to subject to a constant process of improvement and updating.

As we would like to hear the opinions of the professionals about this new resource for supporting maternal education - in terms of its ease of use, suitability and effectiveness - we would like to invite you to attend a group conversation, or focus group, to analyze these issues in depth. Participation is voluntary and only requires you to attend once. This meeting will be held on xxxxx (date), at xxxx (address) and will last approximately 90 minutes. The conversation will be moderated by two researchers from the Research Unit (xx and xx) and will be attended by other professional users of the web (between 6 and 8 people), from different health centers, to talk about the topics under discussion.

Data processing and protection

Following the methodology of a focus group, the conversation generated by the group will be recorded entirely in audio to facilitate its subsequent transcription and analysis. In this task, the qualitative studies company xxx will collaborate with the UIAPB researchers. The company XXX (description) meets all the applicable requirements in terms of quality and data protection, and is based in the Basque Country, at (address xxx). The opinions of the participants will be processed in anonymized form and the confidentiality of the data collected will be guaranteed at all times. The audio files of the recordings and their corresponding transcripts (previously anonymized) will be stored on the computers of the Research Unit,

access to which is restricted to researchers and password-protected. Before the start of the recording, each participant will sign a consent form to authorize their participation in the study.

This study complies with the provisions of REGULATION (EU) 2016/679 OF THE EUROPEAN PARLIAMENT AND OF THE COUNCIL of 27 April 2016 on the protection of natural persons with regard to the processing of personal data and the free movement of such data, and of Organic Law 3/2018, of 5 December 2018 on the Protection of Personal Data and guarantee of digital rights that repeals Organic Law 15/1999 of 5 December 1999, on the protection of personal data. Personal data will be processed by the Basque Health Service, Osakidetza. No data will be transferred to third parties, except under legal obligation. The patient will be informed that they have the right of access, rectification, deletion of their data, and limitation or opposition to its use. In addition, further information on data protection can be found at the following website: http://www.osakidetza.euskadi.eus/protecciondatos

Benefits and risks

At the beginning of the session, the moderators will explain in detail what the technique involves and you will be able to clarify any doubts about the interview. Likewise, the participants will be able to express their opinions freely, in a climate of respect and confidentiality. Participation in the group is entirely altruistic, the main benefit being the possibility of sharing your experience about the website with other colleagues, reflecting on ways to improve it and adapt it to the objective of promoting Maternal Education that is more appropriate to the needs of women today.

Moreover, the moderators will take measures to minimize the impact of any potential risk that you may perceive during the development of the focus group (such as fatigue, discomfort with any questions or conflict between participants). You can withdraw your participation if you wish at any time, even during the interview itself. We remain at your entire disposal to offer any clarification or to answer your questions. If you need more information, you can contact the lead researcher of the study or the Bizkaia Primary Care Research Unit:

- **Isabel Artieta. Zuazo Health Center (Barakaldo). Tel. 946007711**
- **Bizkaia Primary Care Research Unit (Biocruces-Bizkaia). Tel. 946006637.** (Maite Espinosa)

Thank you very much.

DECLARATION OF CONSENT

Study Title: “**Effectiveness and usability of a website for the self-management of women's health needs during pregnancy, childbirth and the postpartum period: EMAeHealth**”

Centre: **Bizkaia Primary Care Research Unit (Biocruces-Bizkaia Institute)**

I (name and surname) declare that

....

I have read the information sheet given to me.
I was able to ask questions about the study.
I have received enough information about the study.
I know that in case of doubt I can go to the lead researcher:

Isabel Artieta. Zuazo Health Center (Barakaldo). Phone 946007711

I understand that my participation in the focus group is voluntary and that I can leave the meeting at any time, without explanation and without repercussion.
I freely give my consent for the meeting to be audio recorded and for the content of the meeting, including my contributions, to be transcribed in anonymized form for later analysis and research purposes.

DATE: PARTICIPANT’S SIGNATURE.

The data collected will be added to a computer database in anonymized form, and be used to evaluate the research. This study complies with the provisions of REGULATION (EU) 2016/679 OF THE EUROPEAN PARLIAMENT AND OF THE COUNCIL of 27 April 2016 on the protection of natural persons with regard to the processing of personal data and the free movement of such data, and of Organic Law 3/2018, of 5 December 2018 on the Protection of Personal Data and guarantee of digital rights that repeals Organic Law 15/1999 of 5 December 1999, on the protection of personal data. Personal data will be processed by the Basque Health Service, Osakidetza. No data will be transferred to third parties, except under legal obligation. The patient will be informed that they have the right of access, rectification, deletion of their data, and limitation or opposition to its use. In addition, further information on data protection can be found at the following website: http://www.osakidetza.euskadi.eus/protecciondatos

THIS STUDY HAS BEEN EVALUATED AND APPROVED BY THE BASQUE CLINICAL RESEARCH ETHICS COMMITTEE
