# Supplementary material for: EMAeHealth, a digital tool for the self-management of women’s health needs during pregnancy, childbirth and the puerperium: protocol for a hybrid effectiveness-implementation study
Source: BMJ Open. 2022 Sep 1;12(9):e055031. doi: 10.1136/bmjopen-2021-055031 (PMC9438065; doi:10.1136/bmjopen-2021-055031)
Supplement: Author's manuscript [file bmjopen-2021-055031.draft_revisions.pdf]

BMJ Open is committed to open peer review. As part of this commitment we make the peer review history of every article we publish publicly available.

When an article is published we post the peer reviewers' comments and the authors' responses online. We also post the versions of the paper that were used during peer review. These are the versions that the peer review comments apply to.

The versions of the paper that follow are the versions that were submitted during the peer review process. They are not the versions of record or the final published versions. They should not be cited or distributed as the published version of this manuscript.

BMJ Open is an open access journal and the full, final, typeset and author-corrected version of record of the manuscript is available on our site with no access controls, subscription charges or pay-per-view fees (http://bmjopen.bmj.com).

If you have any questions on BMJ Open's open peer review process please email
info.bmjopen@bmj.com

BMJ Open

EMAeHealth, digital tool for the self-management of women's health needs during pregnancy, childbirth and the puerperium. Study protocol for an effectiveness-implementation hybrid design.

Journal:	*BMJ Open*
Manuscript ID	bmjopen-2021-055031
Article Type:	Protocol
Date Submitted by the Author:	01-Jul-2021
Complete List of Authors:	Espinosa Cifuentes, Maite; Osakidetza-Basque Health Service, Primary Care Research Unit of Biscay. Healthcare Subdirectorate.; Biocruces Bizkaia Health Research Institute, Primary Health Care researcher<br>Artieta-Pinedo, Isabel; Osakidetza-Basque Health Service, Zuazo Health Centre, Barakaldo, (Bizkaia); Biocruces Bizkaia Health Research Institute, Associate Professor of the School of Nursing, University of the Basque Country, Leioa, Bizkaia, Spain.<br>Paz-pascual, Carmen; Osakidetza-Basque Health Service, Sestao Primary Care Health Centre, Bizkaia; Biocruces Bizkaia Health Research Institute, Lecturer in the Midwifery Training Unit of the Basque Country, Bilbao, Bizkaia, Spain.<br>Bully-Garay, Paola; Biocruces Bizkaia Health Research Institute, Member of the Research Group on Primary health care, prevention and chronic diseases. Statistics<br>Group, emaQ ; Osakidetza-Basque Health Service, Ema-Q group is a multidisciplinary group made up mainly of Basque Health service midwives, as well as other medical professionals, psychologists, quality technicians and research experts: Itziar Estalella (IE), Mª José Trincado (MJT), Inés Cabeza (IC), Mari Pierre Gagnon (MPG), Ana Fernández (AF), Gorane Lozano (GL), Gemma Villanueva (GV), Jesús Sánchez (JS), Amaia Maquibar (AM), David Moreno (DM), Catalina Legarra (CL), Maria Jesús Mulas (MJM), Mónica Blas (MB), Pilar Amorrortu (PA), Sonia Alva; Biocruces Bizkaia Health Research Institute, Basque Country University, Labal University of Canada, Cochrane
Keywords:	Telemedicine < BIOTECHNOLOGY & BIOINFORMATICS, EDUCATION & TRAINING (see Medical Education & Training), Quality in health care < HEALTH SERVICES ADMINISTRATION & MANAGEMENT, Maternal medicine < OBSTETRICS, PRIMARY CARE

SCHOLARONE™
Manuscripts

Manuscript BMJ open

Title page

Title: **EMAeHealth, digital tool for the self-management of women's health needs during pregnancy, childbirth and the puerperium. Study protocol for an effectiveness-implementation hybrid design**.

Full names and institutional addresses of all authors:

Maite Espinosa. (*1,2*) *

Isabel Artieta-Pinedo. (*1,2,4)*

Mª Carmen Paz-Pascual. (*1,2,5*)

Paola Bully Garay. (*2*)

ema-Q Group (*3*)

***Correspondence to** Maite Espinosa. maite.espinosacifuentes@osakidetza.eus

1. Osakidetza-Basque Health Service.
2. Members of the Research Group on Primary health care, prevention and chronic diseases of the Biocruces-Bizkaia Health Research Institute.
3. Ema-Q Group Affiliations: Osakidetza-Basque Health Service, Biocruces-Bizkaia Health Research Institute, Basque Country University, Labal University of Canada, Cochrane
4. Primary Care Midwife Zuazo Health Centre, Barakaldo, (Bizkaia); and Associate Professor of the School of Nursing, University of the Basque Country, Leioa, Bizkaia, Spain.
5. Primary Care Midwife in Sestao Health Centre, Bizkaia; and Lecturer in the Midwifery Training Unit of the Basque Country, Bilbao, Bizkaia, Spain.

Isabel Artieta-Pinedo: mariaisabel.artietapinedo@osakidetza.eus
Carmen Paz-Pascual: mariacarmen.pazpascual@osakidetza.eus
Paola Bully: paola.bully01@gmail.com
Consortia: Ema-Q Group
Ema-Q group is a multidisciplinary group made up mainly of Basque Health service midwives, as well as other medical professionals, psychologists, quality technicians and research experts: Itziar Estalella (IE), Mª José Trincado (MJT), Inés Cabeza (IC), Mari Pierre Gagnon (MPG), Ana Fernández (AF), Gorane Lozano (GL), Gemma Villanueva (GV), Jesús Sánchez (JS), Amaia Maquibar (AM), David Moreno (DM), Catalina Legarra (CL), Maria Jesús Mulas (MJM), Mónica Blas (MB), Pilar Amorrortu (PA), Sonia Alvarez (SA) and Covadonga Pérez (CP).

For peer review only - http://bmjopen.bmj.com/site/about/guidelines.xhtml

Abstract

Introduction: The EHealth can help the user of health services to take a more active role in decision-making and serve the professional to guide the patient in this process. A digital tool has been designed to support Maternal Education (ME), organized into four areas: 1) an Information area, 2) Communication area, 3) Health self-management area; 4) a Clinical data area. The main objective of the study is to evaluate the effectiveness of the EMAeHealth digital tool, and its usability and acceptability under routine conditions.

Methods and analysis: Hybrid implementation-effectiveness design: 1) A cluster-randomized, prospective, longitudinal, multicenter clinical trial to evaluate the effectiveness of EMAeHealth -to improve the health-related quality of life (SF-36), reduce the number of visits to the obstetric emergency service, and other variables.-. The EMAeHealth intervention plus usual care will be compared to receiving only usual care, which includes traditional ME. N= 1080 participants, 540 for each study arm. Two measurements will be made throughout the pregnancy and three in the first 16 weeks postpartum. 2) A mixed method study to evaluate the usability and acceptability of the tool, barriers and facilitators for its use, and implementation in our health system. A qualitative study will be carried out by focus groups (women, professionals and agents involved) and a quantitative analysis of implementation indicators (adoption and frequency of use of EMAeHealth and its areas according to stratification profiles and correlates with other variables). Analysis: By intention to treat, using mixed models taking into account the hierarchical structure of the data, and per protocol to evaluate the effectiveness of the express use of the digital tool.

Ethics and dissemination: Clinical Research Ethics Committee of Euskadi, Spain, (Ref: PI2020044) approved this study. The results from will be actively disseminated through manuscript publications and conference presentations.

Trial registration number: NCT04937049

For peer review only

Strengths and limitations of this study

• Three-quarters of pregnant women and half of postpartum women regularly consult and share experiences on the Internet because it offers them immediate, detailed, entertaining, personalized or practical information.

• EHealth can help the user of health services to take a more active role in decision-making and serve the professional to guide the patient in this process. The participation of patients in the design and evaluation of these new technologies is essential.

For peer review only - http://bmjopen.bmj.com/site/about/guidelines.xhtml

• Involving professionals in the development and evaluation of digital tools also becomes essential but it is not an easy task. The health burden to which they are subjected is a very important barrier.

• The research team has designed a digital tool, called EMAeHealth, which involves healthcare professionals and patients. The tool will complement Maternal Education with resources that facilitate its accessibility and continuity, which will also improve its results.

• The effectiveness of the digital tool, while collecting useful information for its implementation under routine conditions, will be carried out using a hybrid effectiveness-implementation design.

Introduction

Need to adapt Maternal Education to the current context

The fact that practically all nulliparous women attend Maternal Education (ME) classes, and the interest it arouses, together with the fact that at this time of transition in their lives women are more receptive to interventions to promote health, makes ME an opportunity [1,2] which requires continuous improvement, reorienting objectives, format, duration and content to the needs of women in the current context [3,4,5].

Taking into account the bibliography and the needs expressed in focus groups by pregnant and postpartum women in our environment, the research team proposed the redesign of ME [6,7]. ME was redefined as a complex intervention, which should extend from the beginning of pregnancy to the end of the puerperium, focused on the needs of women and with the involvement of multiple health professionals. It is an intervention whose general objective is to train women in making appropriate decisions to (a) improve their health level and that of their family, (b) choose and responsibly face their birth and parenting model, and (c) maintain family and social support networks. The new model of ME includes multiple interventions capable of responding to the health needs of each woman at different moments of the maternity process [8].

Design and relevance of an eHealth tool to support Maternal Education: EMAeHealth

For peer review only - http://bmjopen.bmj.com/site/about/guidelines.xhtml

EHealth interventions are becoming increasingly important and frequent [9]. EHealth can help the user of health services to take a more active role in decision-making and serve the professional to guide the patient in this process [10]. It can also contribute, at organizational level, in a change towards a model that includes the patient's voice to a greater extent [11].

Furthermore, young women from all social strata systematically use the Internet as a source of information about their health [12,13,14,15]. Moreover, the information available on the websites that they consult correlates positively with the decisions they make regarding their health during pregnancy [12,16]. Three-quarters of pregnant women and half of postpartum women regularly consult and share experiences on the Internet because it offers them immediate, detailed, entertaining, personalized or practical information [13,17].

The lack of participation of health professionals in integrating health care and new technologies [12, 18] is felt, since the currently available range of information is not always reliable [19] or contextualized [20]. Since the origin of a website has a direct effect on reliability, the participation of health professionals in the use, advice and generation of new technologies is essential [21].

This team has designed a digital tool called EMAeHealth involving healthcare professionals and patients. The website, adapted to our context, is organized into 4 areas: 1) an Information area, which is open to the public, will provide information based on evidence and is permanently updated by healthcare professionals; 2) a Communication area, which allows women to contact others in a similar situation, through forums or conversations, or consult their reference professional 3) a Health Self-management area, which has valid and reliable self-assessment instruments for checking or reflecting on their own health needs, and decision support tools such as the detection of the moment of delivery or the detection of warning signs and symptoms in the postpartum; 4) a Clinical data area, which allows women to have keep their clinical data, to add the most recent, and share data with other professionals if they wish, under conditions of security. The tool is conceived as a complement that can reinforce maternal health education with resources that facilitate its accessibility, and rapid remote response, which will also improve its results. Its incorporation into the Osakidetza-Basque

For peer review only - http://bmjopen.bmj.com/site/about/guidelines.xhtml

Health Service corporate website will favor its adoption by patients and professionals, as well as its security of use.

Study of the effectiveness of the tool while we are collecting useful information for its implementation in routine conditions

The evaluation of the effectiveness of digital tools in education for health in pregnancy is scarce and has had controversial results [22,23], which makes it essential. In addition, there are barriers and facilitators that modify both the use of digital technology and its effectiveness in health care, where further study is needed [13,16,17,24, 25, 26]. Fortunately, there are hybrid designs that allow clinical intervention testing to be done while gathering information on its delivery during the effectiveness trial and/or its potential for implementation in a real-world situation [27]. There are also frameworks, such as RE-AIM and CFIR, which can help in the task of studying the degree of adoption of an innovation and guide in the data collection, analysis, and interpretation of barriers and facilitators to implementation, understanding which constructs, or sets of constructs promote or inhibit adoption [28, 29]

Aims

The main objective of the study is to evaluate the effectiveness of the EMAeHealth digital tool and its components (Information Area, Communication Area, Health Self-Management Area and Clinical Data Area), as well as its usability and acceptability under routine conditions.

The following are proposed as *specific objectives*:

- To develop the EMAeHealth prototype, through collaborative modeling, with the structure described above, capable of offering: information, responses via algorithm, links to available resources, secure data archiving and monitoring, connection between various systems and the possibility of data analysis establishing continuous feedback and readjustment of the

For peer review only - http://bmjopen.bmj.com/site/about/guidelines.xhtml

information presented. To provide it with content related to pregnancy, childbirth and postpartum.

- To evaluate its effectiveness in 1) improving health-related quality of life, 2) improving self-efficacy for labor and childbirth and self-efficacy in breastfeeding, and 3) reducing the number of visits to the obstetric emergency services and health center in situations of "non-pathological pregnancy", "false labor" and "non-pathological puerperium".
- To evaluate the usability and acceptability of the tool by women and professionals, and any barriers to its implementation.
- To estimate the differential impact that EMAeHealth could have, based on sociodemographic and/or population characteristics.

Methods and analysis

This is an *Effectiveness-implementation hybrid design type I* [26], which combines 1) a multicenter, longitudinal, prospective, cluster-randomized clinical trial to evaluate the effectiveness of EMAeHealth and 2) a mixed-method study to evaluate the usability and acceptability of the tool, barriers and facilitators for its use and implementation in our health system. The mixed method study is made up of a qualitative study by focus groups (women, professionals and agents involved) and a quantitative analysis of implementation indicators. **(Fig. 1)**

The study will be developed over the years 2021 to 2023, being the first year dedicated to the development of the digital tool EMAeHealth. The general scheme of the tool has been designed as described in the introduction and, at this moment, we are finishing the edition of contents and on the software development. We expect to start recruitment in January 2022, until June 2022. The participants will be followed-up for a year, the period of time that the intervention lasts. Final months of 2023 will be dedicated to analysis and diffusion of results.

Regarding the *study population*, all OSAKIDETZA-Basque Health Service public primary care centers that have midwives interested in optimizing their clinical practice by adopting innovations will be

For peer review only - http://bmjopen.bmj.com/site/about/guidelines.xhtml

eligible. In order to consider the *center eligible*, the requirement will be that at least 50% of the midwives belonging to the center have signed the collaboration agreement and the approval of the Head of the Primary Care Center (HPCC).

Osakidetza-Basque Health System is the public healthcare system of the Basque Country, a region located in the north of Spain. Osakidetza was created by the Health Department of the Basque Government in 1983. All the public hospitals and primary care centers of the Basque Region are under this organization, structured into several Integrated Health Organizations (IHO) spread throughout the Basque country. More than 30,000 professionals work for Osakidetza, which could be considered the largest organization in the Basque Country.

For the study of clinical effectiveness, an active surveillance system will be established to identify *eligible patients*, who are Spanish-speaking pregnant women who attend their first consultation with the midwife between week 6 and 10 of gestation. The midwives participating in the study will be in charge of recruiting them consecutively until they have the necessary sample from among the people exposed and not exposed to the EMAeHealth tool. **(Fig. 2)**

To evaluate the quantitative implementation indicators, all pregnant women who have seen their midwives at the start of gestation at the health centers will be included for one year after the implementation of EMAeHealth. The same goes for the *qualitative* evaluation, through focus groups of women and professionals from the health centers.

Random assignment of centers:

The eligible centers will be randomized centrally, blindly, and electronically, in Biocruces-Bizkaia Research Institute, stratified by Integrated Healthcare Organization (IHO) and in a 1: 1 ratio. Therefore, the participating centers will have access to EMAeHealth, while it will not be accessible to the control group centers.

Participating centers

For peer review only - http://bmjopen.bmj.com/site/about/guidelines.xhtml

For peer review only

All the patients in these centers, in the first consultation with the midwife, will be informed that they have EMAeHealth at their disposal and told about the content of the tool. In addition, they will be invited to participate in the study of its clinical effectiveness. If the answer is affirmative, they must sign the informed consent and will be provided with a link to the forms (baseline measurements and questionnaires for each measurement) to fill in electronically. Participants sign an informed consent approved by the Ethics Committee and can leave the study whenever they want.

Control centers

Patients in these centers will not have EMAehealth. They will receive, like the participating group, the usual care and the possibility of attending ME sessions. After being informed of the clinical effectiveness study, they will be asked for consent to participate. Women who sign the informed consent for participation in the study will be provided with the link to the forms for each measurement.

Outcomes

Access to EMAeHealth by women and professionals during the study period will be through access codes, which will allow the research team to track the use made by users of the tool while ensuring the confidentiality of the data. It also makes it possible, under safe conditions, to associate this use with clinical variables from medical records and with the record of the forms completed electronically at each measurement.

Results of clinical effectiveness: The primary outcome is change in Health-related quality of life, measured with the Spanish version of the SF-36 questionnaire [30]. The secondary variables are the number and reason for visits to the obstetric emergency service and to the health center for non-pathological pregnancy situations, and the labor stage at the time of going to the hospital for suspected labor onset (measurement is carried out using the Bishop test/dilation in cm, effacement and breaking of waters (YES/NO)) . As indirect indicators of autonomy in managing their own health and well-being, self-efficacy for labor and childbirth and self-efficacy in breastfeeding will be

For peer review only - http://bmjopen.bmj.com/site/about/guidelines.xhtml

measured using the Spanish version of Lowe's CBSEI scales [31] and the Breastfeeding Self-Efficacy Scale - Short Form. [32]

Measurements of these variables will be performed at weeks 8 and 37 of pregnancy, and at weeks 1, 6, and 16 postpartum. The contact with the woman and the response to the questionnaires will be carried out electronically (via mobile phone).

Indicators of the implementation process: Qualitative indicators: Usability and acceptability of the tool by patients and professionals, as well as barriers and facilitators for its use and adoption in the health system. The qualitative evaluation will be carried out through focus groups of women and professionals, between 6 and 8 per group and as many as necessary until the discourse is saturated [33]. A semi-structured script will be developed based on the theoretical constructs of the CFIR [28] for the sessions, which will also serve as the basis for the analysis. Sessions will be audio-recorded and moderated by an expert in qualitative techniques while an assistant will take notes. Later the sessions will be transcribed and analyzed. Quantitative indicators: adoption and frequency of use of EMAeHealth and its areas according to stratification profiles and correlates with other variables (indicators: patients exposed to the digital tool, by center and group; women with access to EMAeHealth who access the tool once; frequency of use of the tool, frequency of use of the different functions of the tool, etc.).

Predictive, modifying and confounding variables: *Patients*: Age, Parity, Nationality, Race, Social class, Educational level, Marital status, Work situation, Distance to hospital, Spontaneous pregnancy vs assisted reproductive technique, Obstetric history, and other clinical characteristics. *Professionals*: Sex, age, length of time in the position, type of contract, profile of adoption of innovations by professionals, measured by means of a questionnaire by Borracci et al., 2013. *Health centers*: reference IHOs, number of midwives and participation rate, rural or urban environment, and population served.

Statistical methods

For peer review only - http://bmjopen.bmj.com/site/about/guidelines.xhtml

For peer review only

EMAeHealth *Clinical Effectiveness Results*: quantitative comparisons will be made between intervention and control centers, with an intention-to-treat approach. Mixed models (linear or generalized as appropriate to the variable under study), that take into account the hierarchical structure of the data (patients grouped by midwives, midwives grouped by center), will be used and 95% confidence intervals will be calculated. Likewise, per-protocol analysis will be carried out, in which the effectiveness of the express use of both the tool in general and in particular of some of its components will be evaluated, adjusting for possible confounding factors. Statistical analyzes will be carried out by a professional who is not part of the research team.

Implementation process: For the *qualitative* evaluation of the tool and its implementation, the analysis of the focus groups, facilitated by the ATLAS.ti software program, will be structured in three phases: a deductive analysis to determine which CFIR constructs influence its use in real life, followed by an inductive analysis to identify others not included in the CFIR framework that could influence its use, and a third validation phase using triangulation. The analysis results report will be provided to each patient and professional, who will have the opportunity to provide feedback to the team of analysts with their comments ("response validity").

For the *quantitative* evaluation, the distribution of the indicators associated with the use and scope of the tool as well as its components will be described. Measures of central tendency, dispersion and proportions will be calculated, with 95% confidence intervals, and the relationship between the indicators and the predictor/confounding variables will be analyzed using mixed models.

Study size

For the clinical effectiveness study, 18 centers will need to recruit 30 patients each, in each arm of the study. This will make it possible to detect a difference of 2 points as significant in the mental component of the SF-36 test, with an alpha error of less than 5% and power greater than 80%. A 10-point standard deviation is assumed for the mental SF-36 and an intra-center correlation of 0.01.

For peer review only - http://bmjopen.bmj.com/site/about/guidelines.xhtml

The Spanish version of the SF-36 questionnaire has been used to assess the health-related quality of life perceived by pregnant women in several studies [34; 35], showing good psychometric characteristics. The SF-36 questionnaire is made up of 8 domains, of which 4 are oriented towards physical aspects and another 4 towards mental aspects of health-related quality of life. In addition, two summaries are made of this physical and mental component, which explain between 80 and 85% of the variance of the 8 domains [30]. The scores of the questionnaire are transformed on a scale from 0 to 100 points; the higher the score, the better the perception of health-related quality of life. A difference of 5 points in the domain score, or between 2 and 3 points in the summary score is considered clinically relevant. [30]

Patient involvement

In previous studies, a multidisciplinary group that combines midwives, nurses, pediatricians, epidemiologists, psychologists and pregnant and puerperal women participated in the description and prioritization of needs regarding to Maternal Education [3, 6, 7, 8, 15]. In the present study, patients will be involved in the design and evaluation of the tool that will serve as a complement to Maternal Education. The patient will participate in the evaluation of the effectiveness of the digital tool, as study subject, and in the evaluation and improvement of the digital tool, expressing her experience in focus groups of patients designed for this purpose.

Discussion

As the number of health applications for smartphones, wearables, tablets and other digital technologies grows, more people will use them to take control of their own healthcare, and therefore, the issue of the quality of these applications is not a trivial question.

The functions and contents of EMAeHealth have emerged from a research process on the effectiveness of current ME and the needs of women in the period of pregnancy, childbirth and postpartum, as well as the aspects prioritized in ME by midwives and other professionals who work in contact with women during this period. The result is a new ME that primarily aims for the woman

For peer review only - http://bmjopen.bmj.com/site/about/guidelines.xhtml

For peer review only

and her partner to take an active role in her own health and that of their family. It is important to note that this is not a closed process, since the tool itself is designed to provide data on its use and allow great flexibility of content and functions in an endless improvement cycle [37], which favors ME evolving in parallel with the needs of women and their partners.

Given that we are faced with a complex intervention, as defined by the MRC, with multiple components that interact with each other and various organizational levels involved in its implementation [36], ensuring the effectiveness of the intervention would not in itself guarantee its implementation. Randomized controlled trials are regarded as the gold standard for establishing the effectiveness of interventions, when randomization is feasible. However, “effect sizes” do not provide policy makers with information on how an intervention might be replicated in their specific context, or whether trial outcomes will be reproduced. [38]

An intervention may not be highly effective because it is poorly designed or because it has not been correctly implemented; on the contrary, good results can be achieved despite not being implemented as designed, which is why it is so important to study all these aspects, in our case through a hybrid design of implementation effectiveness [27] and following the RE-AIM guidelines and the CFIR constructs as a guide for evaluating implementation and its barriers [28, 29]. This will give way to later studies focused on strategies to increase the use of the tool in our system and also in other contexts. We believe that in this way we will be in a position not only to offer a good support tool for maternal education, but also one that is sustainable and useful for women and professionals.

Ethics and dissemination

The study protocol has been reviewed and ethics approval obtained from the by the Clinical Research Ethics Committee of Euskadi, Spain in November 2020 (Ref: PI2020044).-This approval covers all centers included in the study. Participation in this study will be voluntary; individual written (or online) consent will be sought from all participants. Each participant will receive a study information sheet that outlines the project and expectations in plain language

For peer review only

Manuscripts will be published in open access journals and findings will be further disseminated through local and international Conferences.

Acknowledgements

We would like to acknowledge the statistical support provided by Arturo García, member of Osakidetza Primary care Research group and Biocruces-Bizkaia Health Research Institute.

References

1. Ahldén I, Göransson A, Josefsson A, Alehagen S. Parenthood education in Swedish antenatal care: perceptions of midwives and obstetricians in charge. *J Perinat Educ*. 2008;17(2):21-27. doi:10.1624/105812408X298354
2. *Healthy People Initiative 2020.* https://www.healthypeople.gov/2020/topics-objectives/topic/maternal-infant-and-child-health/objectives
3. Artieta-Pinedo I, Paz-Pascual C, Grandes G, et al. The benefits of antenatal education for the childbirth process in Spain. *Nurs Res*. 2010;59(3):194-202. doi:10.1097/NNR.0b013e3181dbbb4e.
4. Billingham K. Preparing for parenthood: the role of antenatal education. Community Practitioner : the Journal of the Community Practitioners' & Health Visitors' Association. 2011 May;84(5):36-38.
5. McMillan, Anita & Barlow, Jane & Redshaw, Margaret & Britain, Great. (2009). Birth and beyond: a review of the evidence about antenatal education. https://www.researchgate.net/publication/258369289_Birth_and_beyond_a_review_of_the_evidence_about_antenatal_education
6. Paz Pascual C, Artieta Pinedo I, Grandes G, Espinosa M, Gaminde Inda I, Payo Gordon J. [Perceived needs of women regarding maternity. Qualitative study to redesign maternal education]. Aten Primaria. 2016;48(10):657-664. doi:10.1016/j.aprim.2015.12.004

For peer review only - http://bmjopen.bmj.com/site/about/guidelines.xhtml

7. Artieta-Pinedo I, Paz-Pascual C, Grandes G, Espinosa M. Framework for the establishment of a feasible, tailored and effective perinatal education programme. *BMC Pregnancy Childbirth*. 2017;17(1):58. Published 2017 Feb 8. doi:10.1186/s12884-017-1234-7

8. Paz-Pascual C, Artieta-Pinedo I, Grandes G; ema.Q Group. Consensus on priorities in maternal education: results of Delphi and nominal group technique approaches. *BMC Pregnancy Childbirth*. 2019;19(1):264. Published 2019 Jul 24. doi:10.1186/s12884-019-2382-8

9. World Health Organization. Digital Health. https://www.who.int/ehealth/en/

10. Ruiz-Azarola A, Perestelo-Pérez L. Participación ciudadana en salud: formación y toma de decisiones compartida. Informe SESPAS 2012 [Citizens' participation in health: education and shared decision-making. SESPAS Report 2012]. *Gac Sanit*. 2012;26 Suppl 1:158-161. doi:10.1016/j.gaceta.2011.10.005

11. Mora J et al, (2019) Cómo avanzar con el paciente. Modelo afectivo efectivo. The Institute for Health and Strategy S.L. (Si-Health). https://www.sihealth.es/es/publicaciones-eventos/publicaciones/

12. Bert F, Gualano MR, Brusaferro S, et al. Pregnancy e-health: a multicenter Italian cross-sectional study on Internet use and decision-making among pregnant women. *J Epidemiol Community Health*. 2013;67(12):1013-1018. doi:10.1136/jech-2013-202584

13. Hughson JP, Daly JO, Woodward-Kron R, Hajek J, Story D. The Rise of Pregnancy Apps and the Implications for Culturally and Linguistically Diverse Women: Narrative Review. *JMIR Mhealth Uhealth*. 2018;6(11):e189. Published 2018 Nov 16. doi:10.2196/mhealth.9119

14. O'Higgins A, Murphy OC, Egan A, Mullaney L, Sheehan S, Turner MJ. The use of digital media by women using the maternity services in a developed country. Ir Med J. 2014 Nov-Dec;107(10):313-5. PMID: 25556255.

15. Artieta-Pinedo I, Paz-Pascual C, Grandes G, Villanueva G; Ema Q Group. An evaluation of Spanish and English on-line information sources regarding pregnancy, birth and the postnatal period. *Midwifery*. 2018;58:19-26. doi:10.1016/j.midw.2017.12.002

For peer review only - http://bmjopen.bmj.com/site/about/guidelines.xhtml

16. Wallwiener S, Müller M, Doster A, et al. Pregnancy eHealth and mHealth: user proportions and characteristics of pregnant women using Web-based information sources-a cross-sectional study. *Arch Gynecol Obstet*. 2016;294(5):937-944. doi:10.1007/s00404-016-4093-y

17. Lupton D. The use and value of digital media for information about pregnancy and early motherhood: a focus group study. *BMC Pregnancy Childbirth*. 2016;16(1):171. Published 2016 Jul 19. doi:10.1186/s12884-016-0971-3

18. Rodger D, Skuse A, Wilmore M, et al. Pregnant women's use of information and communications technologies to access pregnancy-related health information in South Australia. *Aust J Prim Health*. 2013;19(4):308-312. doi:10.1071/PY13029

19. Brown HM, Bucher T, Collins CE, Rollo ME. A review of pregnancy iPhone apps assessing their quality, inclusion of behaviour change techniques, and nutrition information. *Matern Child Nutr*. 2019;15(3):e12768. doi:10.1111/mcn.12768

20. Kraschnewski JL, Chuang CH, Poole ES, et al. Paging "Dr. Google": does technology fill the gap created by the prenatal care visit structure? Qualitative focus group study with pregnant women. *J Med Internet Res*. 2014;16(6):e147. Published 2014 Jun 3. doi:10.2196/jmir.3385

21. Artieta-Pinedo I, Paz-Pascual C, Grandes G, Villanueva G; Ema Q Group. An evaluation of Spanish and English on-line information sources regarding pregnancy, birth and the postnatal period. *Midwifery*. 2018;58:19-26. doi:10.1016/j.midw.2017.12.002

22. Overdijkink SB, Velu AV, Rosman AN, van Beukering MD, Kok M, Steegers-Theunissen RP. The Usability and Effectiveness of Mobile Health Technology-Based Lifestyle and Medical Intervention Apps Supporting Health Care During Pregnancy: Systematic Review. *JMIR Mhealth Uhealth*. 2018;6(4):e109. Published 2018 Apr 24. doi:10.2196/mhealth.8834

23. Roch G, Borgès Da Silva R, de Montigny F, et al. Impacts of online and group perinatal education: a mixed methods study protocol for the optimization of perinatal health

For peer review only

services. *BMC Health Serv Res*. 2018;18(1):382. Published 2018 May 29. doi:10.1186/s12913-018-3204-9

24. Berland GK, Elliott MN, Morales LS, et al. Health information on the Internet: accessibility, quality, and readability in English and Spanish. *JAMA*. 2001;285(20):2612-2621. doi:10.1001/jama.285.20.2612
25. Risling T, Martinez J, Young J, Thorp-Froslie N. Evaluating Patient Empowerment in Association With eHealth Technology: Scoping Review. *J Med Internet Res*. 2017;19(9):e329. Published 2017 Sep 29. doi:10.2196/jmir.7809
26. Leonardsen AL, Hardeland C, Helgesen AK, Grøndahl VA. Patient experiences with technology enabled care across healthcare settings- a systematic review. BMC Health Serv Res. 2020;20(1):779. Published 2020 Aug 24. doi:10.1186/s12913-020-05633-4
27. Curran GM, Bauer M, Mittman B, Pyne JM, Stetler C. Effectiveness-implementation hybrid designs: combining elements of clinical effectiveness and implementation research to enhance public health impact. Med Care. 2012;50(3):217-226. doi:10.1097/MLR.0b013e3182408812
28. Damschroder LJ, Aron DC, Keith RE, Kirsh SR, Alexander JA, Lowery JC. Fostering implementation of health services research findings into practice: a consolidated framework for advancing implementation science. Implement Sci. 2009;4:50. Published 2009 Aug 7. doi:10.1186/1748-5908-4-50
29. Gaglio B, Shoup JA, Glasgow RE. The RE-AIM framework: a systematic review of use over time. *Am J Public Health*. 2013;103(6):e38-e46. doi:10.2105/AJPH.2013.301299
30. Vilagut G, Ferrer M, Rajmil L, et al. El Cuestionario de Salud SF-36 español: una década de experiencia y nuevos desarrollos [The Spanish version of the Short Form 36 Health Survey: a decade of experience and new developments]. *Gac Sanit*. 2005;19(2):135-150. doi:10.1157/13074369

For peer review only

31. Cunqueiro MJ, Comeche MI, Docampo D. Childbirth Self-Efficacy Inventory: psychometric testing of the Spanish version. *J Adv Nurs*. 2009;65(12):2710-2718. doi:10.1111/j.1365-2648.2009.05161.x

32. Oliver-Roig A, d'Anglade-González ML, García-García B, Silva-Tubio JR, Richart-Martínez M, Dennis CL. The Spanish version of the Breastfeeding Self-Efficacy Scale-Short Form: reliability and validity assessment. *Int J Nurs Stud*. 2012;49(2):169-173. doi:10.1016/j.ijnurstu.2011.08.005

33. Hennink MM, Kaiser BN, Weber MB. What Influences Saturation? Estimating Sample Sizes in Focus Group Research. *Qual Health Res*. 2019;29(10):1483-1496. doi:10.1177/1049732318821692

34. Martínez-Galiano JM, Hernández-Martínez A, Rodríguez-Almagro J, Delgado-Rodríguez M. Quality of Life of Women after Giving Birth: Associated Factors Related with the Birth Process. *J Clin Med*. 2019;8(3):324. Published 2019 Mar 7. doi:10.3390/jcm8030324

35. Schubert KO, Air T, Clark SR, et al. Trajectories of anxiety and health related quality of life during pregnancy. *PLoS One*. 2017;12(7):e0181149. Published 2017 Jul 24. doi:10.1371/journal.pone.0181149

36. Craig P, Dieppe P, Macintyre S, et al. Developing and evaluating complex interventions: the new Medical Research Council guidance. BMJ 2008;337:a1655

37. Artieta-Pinedo I, Paz-Pascual C, Bully P, Espinosa M, Group E. Design of the maternal website “EMAeHealth” supporting decision-making during pregnancy and postpartum: Collaborative Action Research Study. JMIR Formative Research. 23/05/2021:28855 (forthcoming/in press)

38. Moore, GF, Audrey S, Barker M , et al. Process evaluation of complex interventions: Medical Research Council guidance. BMJ 2015;350:h1258. doi: 10.1136/bmj.h1258 https://www.bmj.com/content/bmj/350/bmj.h1258.full.pdf

Footnotes

For peer review only - http://bmjopen.bmj.com/site/about/guidelines.xhtml

Author statement: IA, CP, PB and ME designed the EMAeHealth program, with EMA-Q Group´s collaboration; all of them have been involved in the design of the protocol and have collaborated in writing the manuscript; all authors read, contributed, and finally approved the manuscript. *Contributors*: Arturo García (statistics) and patients involved.

Funding: This study has been funded by the Carlos III National Health Institute, (grant number: PI20/00899.), (Co-funded by European Regional Development Fund "A way to make Europe ").

Disclaimer: The funding source has not been involved in study design, collection, analysis and interpretation of data, writing of the report or in the decision to submit the article for publication.

Conflicts of interests: The authors declare that they have no competing interests.

Patient involvement: See details in the methods and analysis section.

Patient consent for publication: Not required

Ethics approval: Clinical Research Ethics Committee of Euskadi (Spain), in November 2020 (Ref: PI2020044)

Provenance and peer review: Not commissioned; externally peer reviewed

Data sharing statement: Access to the data is feasible by requesting permission from the main researcher, Isabel Artieta. The protocol and the research results will be published. Access to data, ICF, etc. are available by requesting it from the main researcher, Isabel Artieta. (MARIAISABEL.ARTIETAPINEDO@osakidetza.eus)

Word count: 3665 (References and footnotes not included), 4953 (Total)

Figure legends

**Figure 1.** EMAeHealth design description

For peer review only - http://bmjopen.bmj.com/site/about/guidelines.xhtml

Figure 2. EMAeHealth effectiveness flow chart

For peer review only

For peer review only - http://bmjopen.bmj.com/site/about/guidelines.xhtml

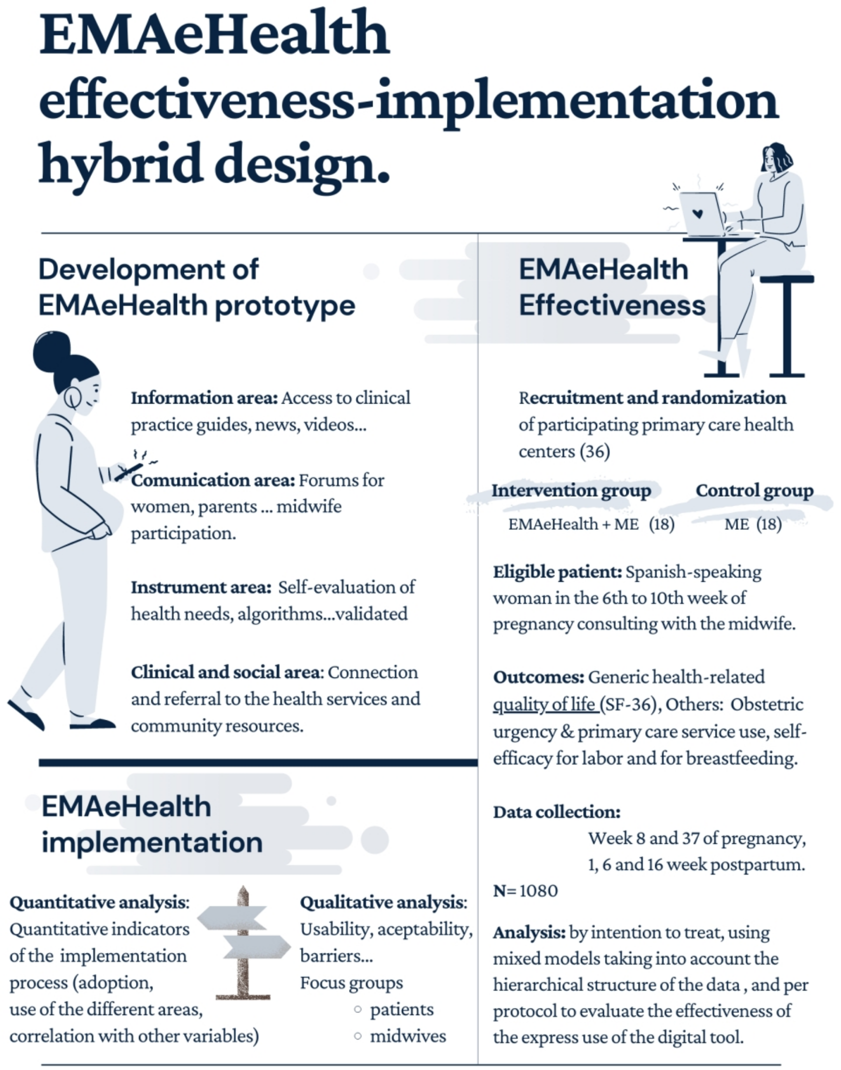

Figure 1. EMAeHealth design description

1060x1500mm (38 x 38 DPI)

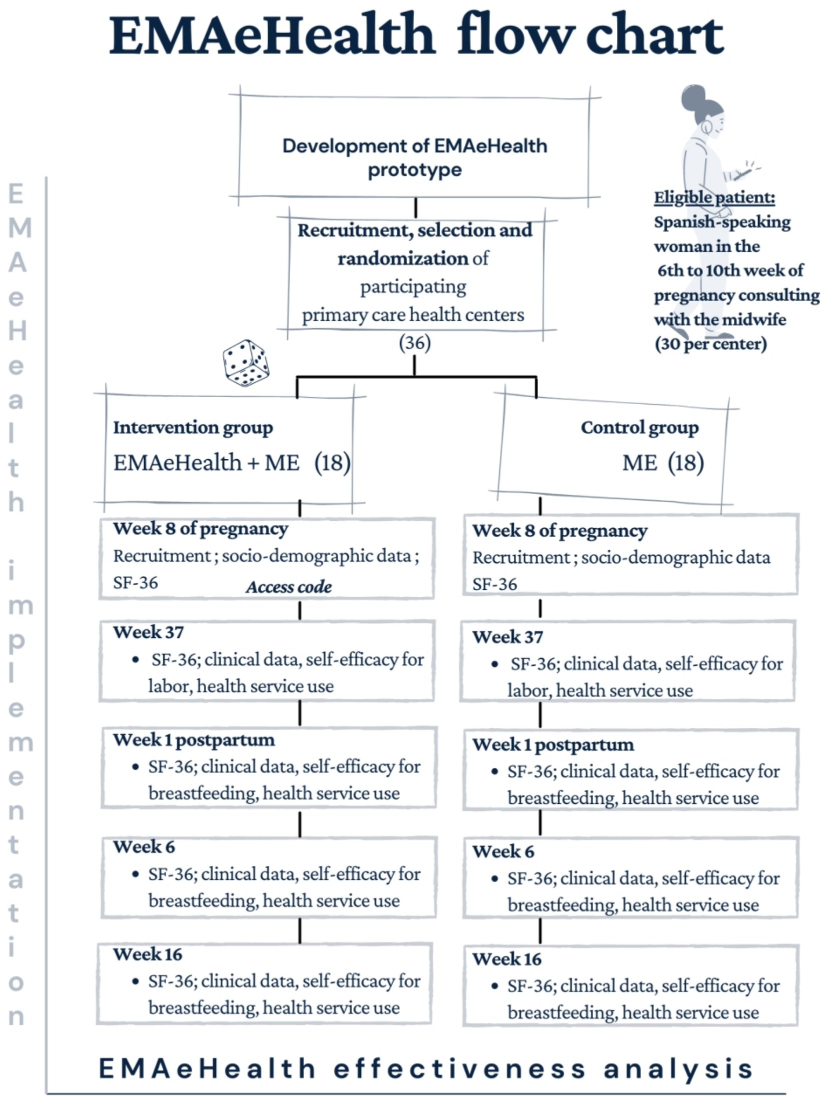

Figure 2. EMAeHealth effectiveness flow chart

1060x1500mm (38 x 38 DPI)

SPIRIT

STANDARD PROTOCOL ITEMS: RECOMMENDATIONS FOR INTERVENTIONAL TRIALS

SPIRIT 2013 Checklist: Recommended items to address in a clinical trial protocol and related documents*

Section/item	Item No	Description
**Administrative information**		
Title	1	**EMAeHealth, digital tool for the self-management of women's health needs during pregnancy, childbirth and the puerperium. Study protocol for an effectiveness-implementation hybrid design**. (Title page. Pag.1)
Trial registration	2a	ClinicalTrials.gov ID: NCT04937049
	2b	
Protocol version	3	Manuscript BMJ- 24-06-2021-v.1_EN.docx
Funding	4	Footnotes section. Funding section. Page 19.
Roles and responsibilities	5a	Footnotes section. Page 19 (Contributors)
	5b	Footnotes section. Page 19 (Funding)
	5c	Footnotes section. Page 19 (Disclaimer)
	5d	Footnotes section. Page 19
**Introduction**		
Background and rationale	6a	Introduction section. Page 3, 4 and 5.
	6b	Methods and analysis section. Page 9
Objectives	7	Aims section. Pages 5 and 6
Trial design	8	Methods and analysis section. Page 6,8,9
**Methods: Participants, interventions, and outcomes**		
Study setting	9	Methods section. Pages 7 and 8.
Eligibility criteria	10	Methods section. Pages 7 (eligible centers) and page 8 (eligible patients)

For peer review only

Interventions	11a	Background page 4 (last paragraph) Methods section, page 9 (participating centers)
	11b	N/A
	11c	N/A
	11d	N/A
Outcomes	12	Methods section. Outcomes (pages 9,10,11)
Participant timeline	13	Methods section. Outcomes (page10) and figure 2
Sample size	14	Methods section. Statistical methods: Study size page 12.
Recruitment	15	Methods section. Figure 1 and pages 7, 8, and 9.
**Methods: Assignment of interventions (for controlled trials)**		
Allocation:		
Sequence generation	16a	Methods section. *Random assignment of centers* (page 9)
Allocation concealment mechanism	16b	Methods section. *Random assignment of centers* (pages 9)
Implementation	16c	Methods section. Random assignment of centers (pages 9, 2nd paragraph), *Participating centers* (page 9 3rd paragraph)
Blinding (masking)	17a	Methods section. Statistical methods: EMAeHealth *Clinical Effectiveness Results* page 11 (paragraph 3)
	17b	N/A
**Methods: Data collection, management, and analysis**		
Data collection methods	18a	Methods section. Outcomes (page 10, paragraph 1,2 and 3)
	18b	N/A
Data management	19	Methods section. Outcomes (pages 10, paragraph 1 and 3)
Statistical methods	20a	Methods section. Statistical methods (pages 11)
	20b	Methods section. Statistical methods (pages 11)
	20c	Methods section. Statistical methods (pages 11)
**Methods: Monitoring**		

For peer review only - http://bmjopen.bmj.com/site/about/guidelines.xhtml

Data monitoring	21a	The research team will oversee and manage the project. A Trial Monitoring Committee has been established and contains a representative group of the researchers (Midwifes, Quality management expert, statistic, psychologist) who are responsible for the conduct of the trial and monitoring the study. There is not competing interest.
	21b	The trial Monitoring Committee will contact the midwives every fortnight to promote capturing the sample and gradually obtaining information on the intervention process. Bimonthly reports will be prepared on progress. Biannual meetings will be held for quality control and ongoing training.
Harms	22	N/A
Auditing	23	Annual reports will be prepared for the ethics committee, the Biocruces-Bizkaia clinical research institute and the Carlos III Health Institute
**Ethics and dissemination**		
Research ethics approval	24	See Ethics approval and consent to participate in Footnotes page 19.
Protocol amendments	25	No important protocol modifications are foreseen because because this is the final version of the protocol approved by the corresponding Ethics committee.
Consent or assent	26a	Methods section page 8 2nd paragraph and page 9, paragraph 3
	26b	N/A
Confidentiality	27	Methods/ Outcomes 1st paragraph.
Declaration of interests	28	Footnotes section: Competing interest page 19.
Access to data	29	The study is ongoing. The datasets that we will use and/or analysed during the study will be available from the corresponding author on reasonable request.
Ancillary and post-trial care	30	N/A
Dissemination policy	31a	The results of the study will be published in open access scientific journals and scientific and professional conferences and meetings. Patient associations will also be informed.

For peer review only

	31b	We will follow the recommendations of ICMJE Authorship eligibility guidelines.
	31c	N/A
**Appendices**		
Informed consent materials	32	Model consent form and other related documentation given to participants and authorised surrogates will be available from the corresponding author on reasonable request.
Biological specimens	33	N/A

*It is strongly recommended that this checklist be read in conjunction with the SPIRIT 2013 Explanation & Elaboration for important clarification on the items. Amendments to the protocol should be tracked and dated. The SPIRIT checklist is copyrighted by the SPIRIT Group under the Creative Commons "Attribution-NonCommercial-NoDerivs 3.0 Unported" license.

For peer review only

For peer review only - http://bmjopen.bmj.com/site/about/guidelines.xhtml

EMAeHealth Spirit timeline table.

	STUDY PERIOD							
	Enrolment	Allocation	Post-allocation					Close-out
TIMEPOINT**	*January 2022-june 2022*	0	*week 8 of pregnancy*	*week 37 of pregnancy*	*Week 1 postpartum*	*Week 6 postpartum*	*Week 16 postpartum*	*June 2023-Dec 2023*
ENROLMENT:	January 2022-june 2022. Enrollment of participants will be after allocation of the centers. It will be done, for both arms, consecutively, until complete the sample (Total N=1080)							
Eligibility screen	Spanish-speaking woman around the 8th week of pregnancy consulting with the midwife of eligible centers.							
Informed consent	First consultation with the midwife between week around 8th week of gestation							
Allocation		The eligible health centers will be randomized centrally, blindly, and electronically, in Biocruces-Bizkaia Research Institute, stratified by Integrated Healthcare Organization (IHO) and in a 1: 1 ratio. Therefore, the participating centers will have access to EMAeHealth,						

For peer review only

		while it will not be accessible to the control group centers.							
**INTERVENTIONS:**									
EMAeHealth +traditional ME (usual care)			←———	———	———	———	———→		
traditional ME (usual care)			←———	———	———	———	———→		
**ASSESSMENTS:**									
Baseline: Socio-demographic data and SF-36			X						
Outcomes: SF-36, clinical outcomes, self-efficacy for labor, self-efficacy for breastfeeding, health service use				X	X	X	X	X	
Predictive, modifying and confounding variables			X	X	X	X	X	X	

For peer review only

BMJ Open

EMAeHealth, a digital tool for the self-management of women's health needs during pregnancy, childbirth and the puerperium: protocol for a hybrid effectiveness-implementation study.

Journal:	*BMJ Open*
Manuscript ID	bmjopen-2021-055031.R1
Article Type:	Protocol
Date Submitted by the Author:	23-Mar-2022
Complete List of Authors:	Espinosa Cifuentes, Maite; Osakidetza-Basque Health Service, Primary Care Research Unit of Biscay. Healthcare Subdirectorate.; Biocruces Bizkaia Health Research Institute, Primary Health Care researcher<br>Artieta-Pinedo, Isabel; Osakidetza-Basque Health Service, Zuazo Health Centre, Barakaldo, (Bizkaia); Biocruces Bizkaia Health Research Institute, Associate Professor of the School of Nursing, University of the Basque Country, Leioa, Bizkaia, Spain.<br>Paz-pascual, Carmen; Osakidetza-Basque Health Service, Sestao Primary Care Health Centre, Bizkaia; Biocruces Bizkaia Health Research Institute, Lecturer in the Midwifery Training Unit of the Basque Country, Bilbao, Bizkaia, Spain.<br>Bully-Garay, Paola; Biocruces Bizkaia Health Research Institute, Member of the Research Group on Primary health care, prevention and chronic diseases. Statistics<br>García-Alvarez, Arturo; Osakidetza-Basque Health Service, Primary Care Research Unit of Biscay. Healthcare Subdirectorate.; Biocruces Bizkaia Health Research Institute<br>Group, emaQ ; Osakidetza-Basque Health Service; Biocruces Bizkaia Health Research Institute, Member of the Research Group on Primary health care, prevention and chronic diseases.
**Primary Subject Heading**:	Medical education and training
Secondary Subject Heading:	Health services research
Keywords:	Telemedicine < BIOTECHNOLOGY & BIOINFORMATICS, EDUCATION & TRAINING (see Medical Education & Training), Quality in health care < HEALTH SERVICES ADMINISTRATION & MANAGEMENT, Maternal medicine < OBSTETRICS, PRIMARY CARE

SCHOLARONE™
Manuscripts

Manuscript BMJ open

Title page

Title: **EMAeHealth, a digital tool for the self-management of women's health needs during pregnancy, childbirth and the puerperium: protocol for a hybrid effectiveness-implementation study.**

Full names and institutional addresses of all authors:

Maite Espinosa. (*1,2*) *

Isabel Artieta-Pinedo. (*1,2,4)*

Mª Carmen Paz-Pascual. (*1,2,5*)

Paola Bully Garay. (*2*)

Arturo García-Alvarez. (1,2)

ema-Q Group (*3*)

***Correspondence to** Maite Espinosa. maite.espinosacifuentes@osakidetza.eus

1. Osakidetza-Basque Health Service.
2. Members of the Research Group on Primary health care, prevention and chronic diseases of the Biocruces-Bizkaia Health Research Institute.
3. Ema-Q Group Affiliations: Osakidetza-Basque Health Service, Biocruces-Bizkaia Health Research Institute, Basque Country University, Labal University of Canada, Cochrane
4. Primary Care Midwife Zuazo Health Centre, Barakaldo, (Bizkaia); and Associate Professor of the School of Nursing, University of the Basque Country, Leioa, Bizkaia, Spain.
5. Primary Care Midwife in Sestao Health Centre, Bizkaia; and Lecturer in the Midwifery Training Unit of the Basque Country, Bilbao, Bizkaia, Spain.

Isabel Artieta-Pinedo: mariaisabel.artietapinedo@osakidetza.eus
Carmen Paz-Pascual: mariacarmen.pazpascual@osakidetza.eus
Paola Bully: paola.bully01@gmail.com
Arturo García-Alvarez: arturo.garciaalvarez@osakidetza.eus
Consortia: Ema-Q Group
Ema-Q group is a multidisciplinary group made up mainly of Basque Health service midwives, as well as other medical professionals, psychologists, quality technicians and research experts: Itziar Estalella (IE), Mª José Trincado (MJT), Inés Cabeza (IC), Mari Pierre Gagnon (MPG), Ana Fernández (AF), Gorane Lozano (GL), Gemma Villanueva (GV), Jesús Sánchez (JS), Amaia Maquibar (AM), David Moreno (DM), Catalina Legarra (CL), Maria Jesús Mulas (MJM), Mónica Blas (MB), Pilar Amorrortu (PA), Sonia Alvarez (SA) and Covadonga Pérez (CP).

For peer review only - http://bmjopen.bmj.com/site/about/guidelines.xhtml

For peer review only

Abstract

Introduction: The EHealth can help the user of health services to take a more active role in decision-making and serve the professional to guide the patient in this process. A digital tool has been designed to support Maternal Education (ME), organized into four areas: 1) an Information area, 2) Communication area, 3) Health self-management area; 4) a Clinical data area. The main objective of the study is to evaluate the effectiveness of the EMAeHealth digital tool, and its usability and acceptability under routine conditions.

Methods and analysis: Hybrid implementation-effectiveness design: 1) A cluster-randomized, prospective, longitudinal, multicenter clinical trial to evaluate the effectiveness of EMAeHealth in -a) improving health-related quality of life (primary outcome), b) improving self-efficacy for labor and childbirth and self-efficacy in breastfeeding, and c) reducing the number of visits to the obstetric emergency services and health center in situations of "non-pathological pregnancy", "false labor" and "non-pathological puerperium". The EMAeHealth intervention plus usual care will be compared to receiving only usual care, which includes traditional ME. N= 1080 participants, 540 for each study arm. Two measurements will be made throughout the pregnancy and three in the first 16 weeks postpartum. 2) A mixed method study to evaluate the usability and acceptability of the tool, barriers and facilitators for its use, and implementation in our health system: Focus groups (women, professionals and agents involved) and a quantitative analysis of implementation indicators. Analysis: By intention to treat, using mixed models taking into account the hierarchical structure of the data, and per protocol to evaluate the effectiveness of the express use of the digital tool.

Ethics and dissemination: Clinical Research Ethics Committee of Euskadi, Spain, (Ref: PI2020044) approved this study. The results from will be actively disseminated through manuscript publications and conference presentations.

Trial registration number: NCT04937049

Strengths and limitations of this study

- EMAeHealth is the prototype of a digital tool that supports decision making during pregnancy and postpartum. One strength is that it has been designed through Collaborative Action Research and is intended to be a complement to Maternal Education.
- A hybrid efficacy-implementation design will allow the effectiveness of EMAeHealth to be evaluated while collecting data on the implementation of the tool in "real-world" conditions.

For peer review only - http://bmjopen.bmj.com/site/about/guidelines.xhtml

- Both patients and professionals have participated in the previous Collaborative Action Research and will be involved in the evaluation of the tool and in its implementation.
- A major strength is that the tool will be effective, will be adapted to our implementation context, and will be widely used because of the way it has been developed and evaluated;
- A weakness is that the results might not be directly extrapolable to a different context and it would be necessary to adapt the tool to the context where it will be implemented.

Introduction

Need to adapt Maternal Education to the current context

Antenatal education classes, including ’childbirth education programs’, ’prenatal classes’, and antenatal groups are attended by an important percentage of pregnant women worldwide. (1,2,3). Both the high attendance of maternal education (ME) classes, and the fact that they take place at a time of transition when readiness for change is higher, suggest that ME can be seen as a valuable opportunity [1,2]. However, it needs to be improved by reorienting the objectives, format, duration and content, to the needs of women in the current context [3,4,5].

The research team proposed the re-design of the ME taking into account the evidence available in the literature regarding the needs of pregnant and postpartum women, theoretical models of health education and models of ME. The needs in relation to the ME, expressed through of focus groups of pregnant women and postpartum women in our environment, were also studied[6, 7].The purpose was to involve women in the process of designing the new EM. Subsequently, a study was conducted to establish priorities with respect to maternal education using the Delphi technique and the nominal group approach. The objective of this study was to identify and prioritize the most important issues in maternal education in order to set goals and evaluate their achievement, i.e., to "make EM evaluable". [8].

ME was redefined as a complex intervention, which should extend from the beginning of pregnancy to the end of the puerperium, focused on the needs of women and with the involvement of multiple

For peer review only - http://bmjopen.bmj.com/site/about/guidelines.xhtml

health professionals. It is an intervention whose general objective should be to enable women in making appropriate decisions to (a) improve their health level and that of their family, (b) choose and responsibly face their birth and parenting model, and (c) maintain family and social support networks. The new model of ME includes multiple interventions capable of responding to the health needs of each woman at different moments of the maternity process [8].

Design and relevance of an eHealth tool to support Maternal Education: EMAeHealth

EHealth interventions are becoming increasingly important and frequent [9]. EHealth can help the user of health services to take a more active role in decision-making and serve the professional to guide the patient in this process [10]. It can also contribute, at organizational level, in a change towards a model that includes the patient's voice to a greater extent [11].

In addition, young women from all social strata consistently use the Internet as a source of information about their health [12,13,14,15]. The information available on the websites they consult is positively correlated with the decisions they make about their health during pregnancy and postpartum [12,16]. Pregnant and postpartum women use the Internet because it offers immediate, detailed, entertaining, personalised or practical information [13,17].

However, the lack of participation of health professionals in integrating health care and new technologies is felt [12, 18], since the currently available range of information is not always reliable [19] or contextualized [20]. Since the origin of a website has a direct effect on reliability, the participation of health professionals in the use, advice and generation of new technologies is essential [21].

This team has designed a prototype of digital tool called EMAeHealth involving healthcare professionals and patients, using a collaborative research process [21]. The website, adapted to our context of implementation, is organized into 4 areas: 1) an Information area, which is open to the public, provide information based on evidence and is permanently updated by healthcare professionals; 2) a Communication area, which allows women to contact others in a similar situation,

For peer review only - http://bmjopen.bmj.com/site/about/guidelines.xhtml

through forums or conversations, or consult their own midwife 3) a Health Self-management area, which has valid and reliable self-assessment instruments for checking or reflecting on their own health needs, and decision support tools such as the detection of the onset of labour or the detection of warning signs and symptoms in the postpartum; 4) a Clinical data area, which allows women to have keep their clinical data, to add the most recent, and share data with other professionals if they wish, under conditions of security. The tool is conceived as a complement that can reinforce Maternal Education with resources that facilitate its accessibility and rapid remote response, which will also improve its results. Its incorporation into the Osakidetza-Basque Health Service corporate website will favor its adoption by patients and professionals, as well as its security of use.

The usual phases for the development of a digital tool are analysis, development, testing and production. The prototype is the result of the analysis, a proposal of the architecture and technical requirements that the digital tool must have in order to respond to the users´ needs. This is followed by development -or programming- and testing, which are carried out almost simultaneously, to detect and solve errors. Once the tool is ready, it is deployed in a production environment, making it available to all users of the system, in our case the Osakidetza-Basque Health Service corporate website.

Study of the effectiveness of the tool while we are collecting useful information for its implementation in routine conditions

The evaluation of the effectiveness of digital tools aimed at health education during pregnancy is scarce and has had controversial results [22,23]; consequently, its realization becomes absolutely essential. In addition, there are barriers and facilitators that modify both the use of digital technology and its effectiveness in health care, where further study is needed [13,16,17,24, 25, 26]. Fortunately, there are hybrid designs that allow testing of the clinical intervention and, at the same time, during the trial, gathering information on its application and/or its potential for application in real-world conditions [27]. There are also frameworks, such as RE-AIM and CFIR, which can help in the task of

For peer review only

For peer review only - http://bmjopen.bmj.com/site/about/guidelines.xhtml

studying the degree of adoption of an innovation and guide in the data collection, analysis and interpretation of barriers and facilitators to implementation. [28, 29]

Aims

The main objective of the study is to evaluate the effectiveness of the EMAeHealth digital tool and its components (Information Area, Communication Area, Health Self-Management Area and Clinical Data Area), as well as its usability and acceptability under routine conditions.

The following are proposed as *specific objectives*:

- To develop, test and deploy the EMAeHealth tool [21], with the structure described above, capable of offering: information, responses via algorithm, links to available resources, secure data archiving and monitoring, connection between various systems and the possibility of data analysis establishing continuous feedback and readjustment of the information presented. To provide it with content related to pregnancy, childbirth and postpartum.
- To evaluate its effectiveness in 1) improving health-related quality of life, 2) improving self-efficacy for labour and childbirth and self-efficacy in breastfeeding, and 3) reducing the number of visits to the obstetric emergency services and health center in situations of "non-pathological pregnancy", "false labour" and "non-pathological puerperium".
- To evaluate the usability and acceptability of the tool by women and professionals, and any barriers to its implementation.
- To estimate the differential impact that EMAeHealth could have, based on sociodemographic and/or population characteristics.

Methods and analysis

This is an *Effectiveness-implementation hybrid design type I* [26], which combines 1) a multicenter, longitudinal, prospective, cluster-randomized clinical trial to evaluate the effectiveness of EMAeHealth and 2) a mixed-method study to evaluate the usability and acceptability of the tool,

For peer review only - http://bmjopen.bmj.com/site/about/guidelines.xhtml

barriers and facilitators for its use and implementation in our health system. The mixed method study is made up of a qualitative study by focus groups (women, professionals and agents involved) and a quantitative analysis of implementation indicators. **(Fig. 1)**

For the effectiveness study, we have chosen a randomized cluster design to avoid, among other issues, contamination bias among women from the same center, since they are likely to coincide, for example, in Maternal Education classes. In addition, as the intervention has been designed for shared decision-making, it did not seem appropriate for the same midwife to have women belonging to control and intervention.

The study will be conducted between 2021 and 2023, with the first year dedicated to the development of the EMAeHealth digital tool. The general structure of the tool is described in the introduction. In the development of the prototype, the International Patient Decision Aid Standards (IPDAS) were applied, using a collaborative action research process, involving experts and patients, with qualitative research techniques, as well as focus groups, prioritization and consensus techniques [21]. At this time, we are finalizing content editing and the software development and testing. We expect to start recruitment in January 2022, until June 2022. The participants will be followed-up for a year, the period of time that the intervention lasts. Final months of 2023 will be dedicated to analysis and diffusion of results.

Regarding the *study population*, all OSAKIDETZA-Basque Health Service public primary care centers that have midwives interested in optimizing their clinical practice by adopting innovations will be eligible. In order to consider the *center eligible*, the requirement will be that at least 50% of the midwives belonging to the center have signed a collaboration agreement and the approval of the Head of the Primary Care Center (HPCC).

Osakidetza-Basque Health System is the public healthcare system of the Basque Country, a region located in the north of Spain. Osakidetza was created by the Health Department of the Basque Government in 1983. All the public hospitals and primary care centers of the Basque Region are

For peer review only - http://bmjopen.bmj.com/site/about/guidelines.xhtml

under this organization, structured into several Integrated Health Organizations (IHO) spread throughout the Basque country. More than 30,000 professionals work for Osakidetza, which could be considered the largest organization in the Basque Country.

For the study of clinical effectiveness, an active surveillance system will be established to identify *eligible patients*, who are Spanish-speaking pregnant women who attend their first consultation with the midwife between week 6 and 10 of gestation. Regarding the clinical profile of them, they are low-risk pregnant women. The midwives participating in the study will be in charge of recruiting them consecutively until they have the necessary sample from among the people exposed and not exposed to the EMAeHealth tool. **(Fig. 2)**

To evaluate the quantitative implementation indicators, all pregnant women who have seen their midwives at the start of gestation at the health centers will be included for one year after the implementation of EMAeHealth. The same goes for the *qualitative* evaluation, through focus groups of women and professionals from the health centers *(model consent form as a supplementary file)*.

Random assignment of centers:

The eligible centers will be randomized centrally, blindly, and electronically, in Biocruces-Bizkaia Research Institute, stratified by Integrated Healthcare Organization (IHO) and in a 1: 1 ratio. Therefore, the intervention allocated centres will have access to EMAeHealth, while it will not be accessible to the control group centers. Integrated Health Organizations are groups of health centers within the same geographic area, with similar characteristics and population, under the direction of the same hospital. Therefore, we assume that there are no pre-existing differences in the quality of care provided between centers that belong to the same integrated health organization.

Intervention centers

All the patients in these centers, in the first consultation with the midwife, will be informed that EMAeHealth is available to them, and the content of the tool will be told to them. In addition, they

For peer review only - http://bmjopen.bmj.com/site/about/guidelines.xhtml

will be invited to participate in the study of its clinical effectiveness. If the answer is affirmative, they must sign the informed consent and will be provided with a link to the forms (baseline measurements and questionnaires for each measurement) to fill in electronically. Participants sign an informed consent approved by the Ethics Committee and can leave the study whenever they want *(model consent form as a supplementary file)*.

Control centers

Patients in these centers will not have EMAehealth. They will receive, like the intervention group, the usual care and the possibility of attending ME sessions, but not EMAeHealth. After being informed of the clinical effectiveness study, they will be asked for consent to participate *(see supplementary file).* Women who sign the informed consent for participation in the study will be provided with the link to the forms for each measurement.

Outcomes

Access to EMAeHealth by women and professionals during the study period will be through access codes, which will allow the research team to track the use made by users of the tool while ensuring the confidentiality of the data. It also makes it possible, under safe conditions, to associate this use with clinical variables from medical records and with the record of the forms completed electronically at each measurement.

Results of clinical effectiveness: The primary outcome is change in Health-related quality of life, measured with the Spanish version of the SF-36 questionnaire [30]. The secondary variables are the number and reason for visits to the obstetric emergency service and to the health center for non-pathological pregnancy situations, and the labor stage at the time of going to the hospital for suspected labor onset (measurement is carried out using the Bishop test/dilation in cm, effacement and breaking of waters (YES/NO)) . As indirect indicators of autonomy in managing their own health and well-being, self-efficacy for labor and childbirth and self-efficacy in breastfeeding will be

For peer review only - http://bmjopen.bmj.com/site/about/guidelines.xhtml

measured using the Spanish version of Lowe's CBSEI scales [31] and the Breastfeeding Self-Efficacy Scale - Short Form. [32]

Measurements of these variables will be performed at weeks 8 and 37 of pregnancy, and at weeks 1, 6, and 16 postpartum. The contact with the woman and the response to the questionnaires will be carried out electronically (via mobile phone).

Quality of life (QoL) is a parameter that has been defined and recently taken into account as a health indicator by WHO [33]. The concept of QV could be defined as an individual's assessment of his or her own state in relation to his or her needs [33], so it seems appropriate that it should be the primary outcome measure. The Spanish version of the SF-36 questionnaire has been used to assess the health-related quality of life perceived by pregnant women in several studies [33; 34], showing good psychometric characteristics. The SF-36 questionnaire is made up of 8 domains, of which 4 are oriented towards physical aspects and another 4 towards mental aspects of health-related quality of life. In addition, two summaries are made of this physical and mental component, which explain between 80 and 85% of the variance of the 8 domains [30]. The scores of the questionnaire are transformed on a scale from 0 to 100 points; the higher the score, the better the perception of health-related quality of life. A difference of 5 points in the domain score, or between 2 and 3 points in the summary score is considered clinically relevant. [30]

Indicators of the implementation process: Qualitative indicators: Usability and acceptability of the tool by patients and professionals, as well as barriers and facilitators for its use and adoption in the health system. The qualitative evaluation will be carried out through focus groups of women and professionals, between 6 and 8 per group and as many as necessary until the discourse is saturated [35]. A semi-structured script will be developed for the sessions, based on the theoretical constructs of the CFIR [28], which will also serve as the basis for the analysis. Sessions will be audio-recorded and moderated by an expert in qualitative techniques while an assistant will take notes. Later the sessions will be transcribed and analysed. Quantitative indicators: adoption and frequency of use of EMAeHealth and its areas according to stratification profiles and correlates with other variables

For peer review only - http://bmjopen.bmj.com/site/about/guidelines.xhtml

(indicators: patients exposed to the digital tool, by center and group; women with access to EMAeHealth who access the tool once; frequency of use of the tool, frequency of use of the different functions of the tool, etc.).

Predictive, modifying and confounding variables: *Patients*: Age, Parity, Nationality, Race, Social class, Educational level, Marital status, Work situation, Distance to hospital, Spontaneous pregnancy vs assisted reproductive technique, Obstetric history, and other clinical characteristics. *Professionals*: Sex, age, length of time in the position, type of contract, profile of adoption of innovations by professionals, measured by means of a questionnaire by Borracci et al., 2013. *Health centers*: reference IHOs, number of midwives and participation rate, rural or urban environment, and population served. **(Fig. 3)**

Statistical methods

EMAeHealth *Clinical Effectiveness Results*: quantitative comparisons will be made between intervention and control centers, with an intention-to-treat approach. Mixed models (linear or generalized as appropriate to the variable under study), that take into account the hierarchical structure of the data (patients grouped by midwives, midwives grouped by center), will be used and 95% confidence intervals will be calculated. Likewise, per-protocol analysis will be carried out, in which the effectiveness of the express use of both the tool in general and in particular of some of its components will be evaluated, adjusting for possible confounding factors. Statistical analysis will be carried out by a professional who is not part of the research team.

Implementation process: For the *qualitative* evaluation of the tool and its implementation, the analysis of the focus groups, facilitated by the ATLAS.ti software program, will be structured in three phases: a deductive analysis to determine which CFIR constructs influence its use in real life, followed by an inductive analysis to identify others not included in the CFIR framework that could influence its use, and a third validation phase using triangulation. The analysis results report will be provided

For peer review only - http://bmjopen.bmj.com/site/about/guidelines.xhtml

to each patient and professional, who will have the opportunity to provide feedback to the team of analysts with their comments ("response validity").

For the *quantitative* evaluation, the distribution of the indicators associated with the use and scope of the tool as well as its components will be described. Measures of central tendency, dispersion and proportions will be calculated, with 95% confidence intervals, and the relationship between the indicators and the predictor/confounding variables will be analyzed using mixed models.

Study size

For the clinical effectiveness study, 18 centers will need to recruit 30 patients each, in each arm of the study (N=1080). This will make it possible to detect a difference of 2 points as significant in the mental component of the SF-36 test, with an alpha error of less than 5% and power greater than 80%. A 10-point standard deviation is assumed for the mental SF-36 and an intra-cluster correlation of 0.01.

The calculation took into account the cluster design of the study and it was carried out using the National Institute of Health GRT sample size calculator* which assumes that the analysis will employ mixed-model regression methods.

* Research Methods Resources: National Institutes of Health. [Accessed 02/01/2020]. Available from: https://researchmethodsresources.nih.gov/grt-calculator

Patient involvement

In previous studies, multidisciplinary groups of midwives, nurses, paediatricians, epidemiologists, psychologists and pregnant and postpartum women were involved in the description and prioritisation of ME needs, as well as in the redesign of the ME and the design of an EMAeHealth tool to complement it [3, 6, 7, 8, 8, 15, 21]. In the present study, patients will be involved in testing the tool under construction, assessing its usability, acceptability and evaluating barriers and facilitators

For peer review only - http://bmjopen.bmj.com/site/about/guidelines.xhtml

for its implementation in "real-world conditions". In addition, women will participate as study subjects in the evaluation of the effectiveness of the digital tool.

Ethics and dissemination

The study protocol has been reviewed and ethics approval obtained from the by the Clinical Research Ethics Committee of Euskadi, Spain in November 2020 (Ref: PI2020044).-This approval covers all centers included in the study. Participation in this study will be voluntary; individual written (or online) consent will be sought from all participants. Each participant will receive a study information sheet that outlines the project and expectations in plain language

Manuscripts will be published in open access journals and findings will be further disseminated through local and international Conferences.

Acknowledgements

We would like to acknowledge the collaboration of those interested in the design of the EMAeHealth tool, the grant received by the Carlos III Health Institute in collaboration with the ISCII- Subdirectorate General for Evaluation and Promotion of Research (European Regional Development Fund-FEDER) and the support of Biocruces Bizkaia Health Research Institute.

References

1. Gagnon AJ, Sandall J. Individual or group antenatal education for childbirth or parenthood, or both. Cochrane Database of Systematic Reviews 2007, Issue 3. Art. No.: CD002869. DOI: 10.1002/14651858.CD002869.pub2.
2. https://www.nice.org.uk › Default › ***Antenatal*** Education Evidence Review Final.docx
3. Artieta-Pinedo I, Paz-Pascual C, Grandes G, et al. The benefits of antenatal education for the childbirth process in Spain. *Nurs Res.* 2010;59(3):194-202. doi:10.1097/NNR.0b013e3181dbbb4e.

For peer review only - http://bmjopen.bmj.com/site/about/guidelines.xhtml

4. Billingham K. Preparing for parenthood: the role of antenatal education. Community Practitioner : the Journal of the Community Practitioners' & Health Visitors' Association. 2011 May;84(5):36-38.

5. McMillan, Anita & Barlow, Jane & Redshaw, Margaret & Britain, Great. (2009). Birth and beyond: a review of the evidence about antenatal education. https://www.researchgate.net/publication/258369289_Birth_and_beyond_a_review_of_the_evidence_about_antenatal_education

6. Paz Pascual C, Artieta Pinedo I, Grandes G, Espinosa M, Gaminde Inda I, Payo Gordon J. [Perceived needs of women regarding maternity. Qualitative study to redesign maternal education]. Aten Primaria. 2016;48(10):657-664. doi:10.1016/j.aprim.2015.12.004

7. Artieta-Pinedo I, Paz-Pascual C, Grandes G, Espinosa M. Framework for the establishment of a feasible, tailored and effective perinatal education programme. *BMC Pregnancy Childbirth*. 2017;17(1):58. Published 2017 Feb 8. doi:10.1186/s12884-017-1234-7

8. Paz-Pascual C, Artieta-Pinedo I, Grandes G; ema.Q Group. Consensus on priorities in maternal education: results of Delphi and nominal group technique approaches. *BMC Pregnancy Childbirth*. 2019;19(1):264. Published 2019 Jul 24. doi:10.1186/s12884-019-2382-8

9. World Health Organization. Digital Health. https://www.who.int/ehealth/en/

10. Ruiz-Azarola A, Perestelo-Pérez L. Participación ciudadana en salud: formación y toma de decisiones compartida. Informe SESPAS 2012 [Citizens' participation in health: education and shared decision-making. SESPAS Report 2012]. *Gac Sanit*. 2012;26 Suppl 1:158-161. doi:10.1016/j.gaceta.2011.10.005

11. Mora J et al, (2019) Cómo avanzar con el paciente. Modelo afectivo efectivo. The Institute for Health and Strategy S.L. (Si-Health). https://www.sihealth.es/es/publicaciones-eventos/publicaciones/

For peer review only

12. Bert F, Gualano MR, Brusaferro S, et al. Pregnancy e-health: a multicenter Italian cross-sectional study on Internet use and decision-making among pregnant women. *J Epidemiol Community Health*. 2013;67(12):1013-1018. doi:10.1136/jech-2013-202584
13. Hughson JP, Daly JO, Woodward-Kron R, Hajek J, Story D. The Rise of Pregnancy Apps and the Implications for Culturally and Linguistically Diverse Women: Narrative Review. *JMIR Mhealth Uhealth*. 2018;6(11):e189. Published 2018 Nov 16. doi:10.2196/mhealth.9119
14. O'Higgins A, Murphy OC, Egan A, Mullaney L, Sheehan S, Turner MJ. The use of digital media by women using the maternity services in a developed country. Ir Med J. 2014 Nov-Dec;107(10):313-5. PMID: 25556255.
15. Artieta-Pinedo I, Paz-Pascual C, Grandes G, Villanueva G; Ema Q Group. An evaluation of Spanish and English on-line information sources regarding pregnancy, birth and the postnatal period. *Midwifery*. 2018;58:19-26. doi:10.1016/j.midw.2017.12.002
16. Wallwiener S, Müller M, Doster A, et al. Pregnancy eHealth and mHealth: user proportions and characteristics of pregnant women using Web-based information sources-a cross-sectional study. *Arch Gynecol Obstet*. 2016;294(5):937-944. doi:10.1007/s00404-016-4093-y
17. Lupton D. The use and value of digital media for information about pregnancy and early motherhood: a focus group study. *BMC Pregnancy Childbirth*. 2016;16(1):171. Published 2016 Jul 19. doi:10.1186/s12884-016-0971-3
18. Rodger D, Skuse A, Wilmore M, et al. Pregnant women's use of information and communications technologies to access pregnancy-related health information in South Australia. *Aust J Prim Health*. 2013;19(4):308-312. doi:10.1071/PY13029
19. Brown HM, Bucher T, Collins CE, Rollo ME. A review of pregnancy iPhone apps assessing their quality, inclusion of behaviour change techniques, and nutrition information. *Matern Child Nutr*. 2019;15(3):e12768. doi:10.1111/mcn.12768

For peer review only - http://bmjopen.bmj.com/site/about/guidelines.xhtml

20. Kraschnewski JL, Chuang CH, Poole ES, et al. Paging "Dr. Google": does technology fill the gap created by the prenatal care visit structure? Qualitative focus group study with pregnant women. *J Med Internet Res*. 2014;16(6):e147. Published 2014 Jun 3. doi:10.2196/jmir.3385

21. Artieta-Pinedo I, Paz-Pascual C, Bully P, Espinosa M, EmaQ Group. Design of the Maternal Website EMAeHealth That Supports Decision-Making During Pregnancy and in the Postpartum Period: Collaborative Action Research Study.JMIR Form Res 2021; 5(8):e28855.URL: https://formative.jmir.org/2021/8/e28855. DOI: 10.2196/28855

22. Overdijkink SB, Velu AV, Rosman AN, van Beukering MD, Kok M, Steegers-Theunissen RP. The Usability and Effectiveness of Mobile Health Technology-Based Lifestyle and Medical Intervention Apps Supporting Health Care During Pregnancy: Systematic Review. *JMIR Mhealth Uhealth*. 2018;6(4):e109. Published 2018 Apr 24. doi:10.2196/mhealth.8834

23. Roch G, Borgès Da Silva R, de Montigny F, et al. Impacts of online and group perinatal education: a mixed methods study protocol for the optimization of perinatal health services. *BMC Health Serv Res*. 2018;18(1):382. Published 2018 May 29. doi:10.1186/s12913-018-3204-9

24. Berland GK, Elliott MN, Morales LS, et al. Health information on the Internet: accessibility, quality, and readability in English and Spanish. *JAMA*. 2001;285(20):2612-2621. doi:10.1001/jama.285.20.2612

25. Risling T, Martinez J, Young J, Thorp-Froslie N. Evaluating Patient Empowerment in Association With eHealth Technology: Scoping Review. *J Med Internet Res*. 2017;19(9):e329. Published 2017 Sep 29. doi:10.2196/jmir.7809

26. Leonardsen AL, Hardeland C, Helgesen AK, Grøndahl VA. Patient experiences with technology enabled care across healthcare settings- a systematic review. BMC Health Serv Res. 2020;20(1):779. Published 2020 Aug 24. doi:10.1186/s12913-020-05633-4

27. Curran GM, Bauer M, Mittman B, Pyne JM, Stetler C. Effectiveness-implementation hybrid designs: combining elements of clinical effectiveness and implementation research to

For peer review only - http://bmjopen.bmj.com/site/about/guidelines.xhtml

enhance public health impact. Med Care. 2012;50(3):217-226. doi:10.1097/MLR.0b013e3182408812

28. Damschroder LJ, Aron DC, Keith RE, Kirsh SR, Alexander JA, Lowery JC. Fostering implementation of health services research findings into practice: a consolidated framework for advancing implementation science. Implement Sci. 2009;4:50. Published 2009 Aug 7. doi:10.1186/1748-5908-4-50

29. Gaglio B, Shoup JA, Glasgow RE. The RE-AIM framework: a systematic review of use over time. *Am J Public Health*. 2013;103(6):e38-e46. doi:10.2105/AJPH.2013.301299

30. Vilagut G, Ferrer M, Rajmil L, et al. El Cuestionario de Salud SF-36 español: una década de experiencia y nuevos desarrollos [The Spanish version of the Short Form 36 Health Survey: a decade of experience and new developments]. *Gac Sanit*. 2005;19(2):135-150. doi:10.1157/13074369

31. Cunqueiro MJ, Comeche MI, Docampo D. Childbirth Self-Efficacy Inventory: psychometric testing of the Spanish version. *J Adv Nurs*. 2009;65(12):2710-2718. doi:10.1111/j.1365-2648.2009.05161.x

32. Oliver-Roig A, d'Anglade-González ML, García-García B, Silva-Tubio JR, Richart-Martínez M, Dennis CL. The Spanish version of the Breastfeeding Self-Efficacy Scale-Short Form: reliability and validity assessment. *Int J Nurs Stud*. 2012;49(2):169-173. doi:10.1016/j.ijnurstu.2011.08.005

33. Martínez-Galiano JM, Hernández-Martínez A, Rodríguez-Almagro J, Delgado-Rodríguez M. Quality of Life of Women after Giving Birth: Associated Factors Related with the Birth Process. *J Clin Med*. 2019;8(3):324. Published 2019 Mar 7. doi:10.3390/jcm8030324

34. Schubert KO, Air T, Clark SR, et al. Trajectories of anxiety and health related quality of life during pregnancy. *PLoS One*. 2017;12(7):e0181149. Published 2017 Jul 24. doi:10.1371/journal.pone.0181149

For peer review only

35. Hennink MM, Kaiser BN, Weber MB. What Influences Saturation? Estimating Sample Sizes in Focus Group Research. *Qual Health Res*. 2019;29(10):1483-1496. doi:10.1177/1049732318821692

Footnotes

Author statement: IA, CP, PB and ME designed the EMAeHealth program, with EMA-Q Group´s collaboration, AG has collaborated on the statistics and on the revision of the article. ; All of them have been involved in the design of the protocol and have collaborated in writing the manuscript; all authors read, contributed, and finally approved the manuscript. *Contributors*: Arturo García (statistics) and patients involved.

Funding: This study has been funded by the Carlos III National Health Institute, (grant number: PI20/00899.), (Co-funded by European Regional Development Fund "A way to make Europe ").

Disclaimer: The funding source has not been involved in study design, collection, analysis and interpretation of data, writing of the report or in the decision to submit the article for publication.

Conflicts of interests: The authors declare that they have no competing interests.

Patient involvement: See details in the methods and analysis section.

Patient consent for publication: Not required

Ethics approval: Clinical Research Ethics Committee of Euskadi (Spain), in November 2020 (Ref: PI2020044)

Provenance and peer review: Not commissioned; externally peer reviewed

Data sharing statement: Access to the data is feasible by requesting permission from the main researcher, Isabel Artieta. The protocol and the research results will be published. Access to data, ICF, etc. are available by requesting it from the main researcher, Isabel Artieta. (MARIAISABEL.ARTIETAPINEDO@osakidetza.eus)

For peer review only

Word count: 3789 (References and footnotes not included), 5163 (Total)

Figure legends

Figure 1. EMAeHealth design description

Figure 2. EMAeHealth effectiveness flow chart

Figure 3. SPIRIT-Timetable

For peer review only

For peer review only - http://bmjopen.bmj.com/site/about/guidelines.xhtml

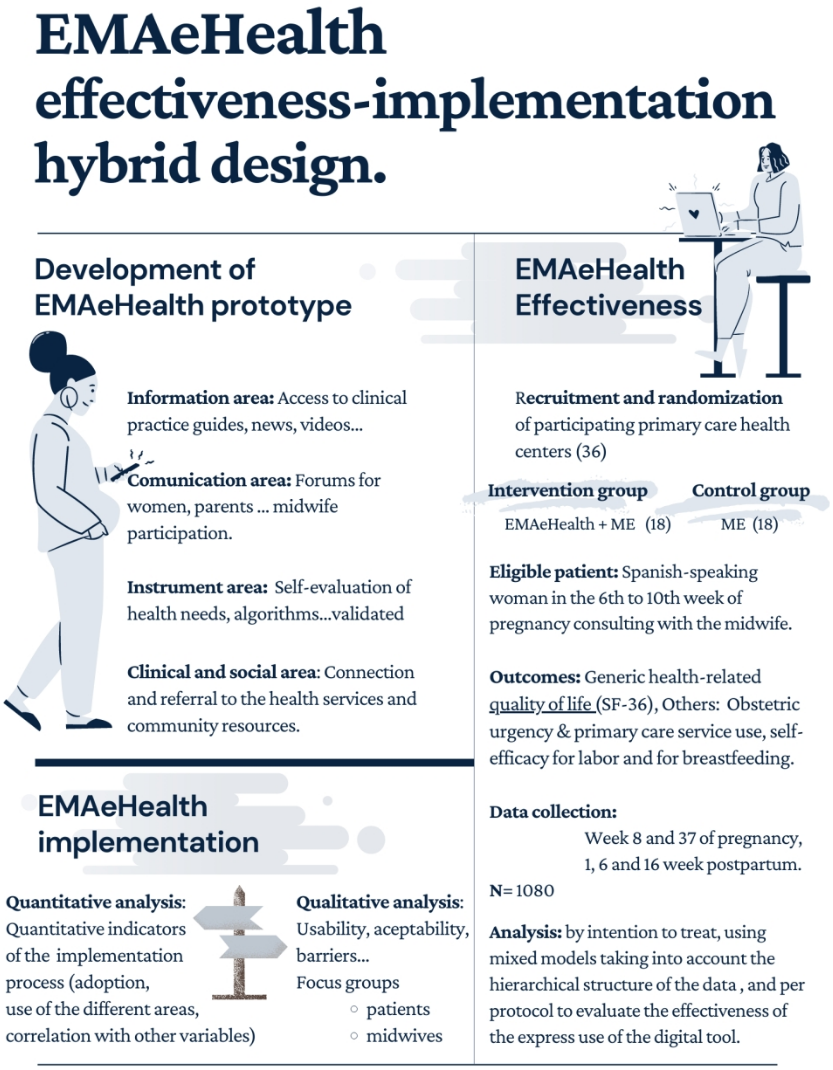

Figure 1. EMAeHealth design description

1060x1500mm (38 x 38 DPI)

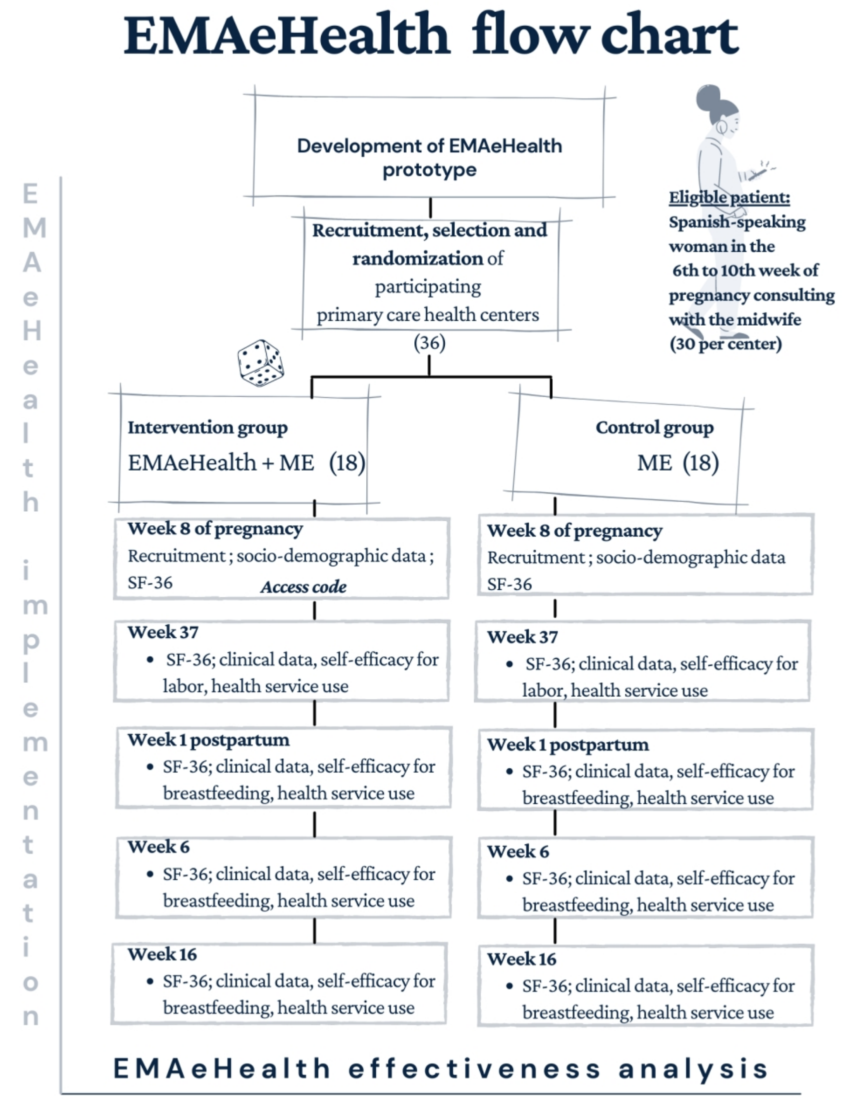

Figure 2. EMAeHealth effectiveness flow chart

1060x1500mm (38 x 38 DPI)

EMAeHealth Spirit timeline table.

	STUDY PERIOD							
	**Enrolment**	**Allocation**	**Post-allocation**					**Close-out**
**TIMEPOINT****	***January 2022-june 2022***	**0**	***week 8 of pregnancy***	***week 37 of pregnancy***	***Week 1 postpartum***	***Week 6 postpartum***	***Week 16 postpartum***	***June 2023-Dec 2023***
**ENROLMENT:**	January 2022-june 2022. Enrollment of participants will be after allocation of the centers. It will be done, for both arms, consecutively, until complete the sample (Total N=1080)							
**Eligibility screen**	Spanish-speaking woman around the 8th week of pregnancy consulting with the midwife of eligible centers.							
**Informed consent**	First consultation with the midwife between week around 8th week of gestation							
**Allocation**		The eligible health centers will be randomized centrally, blindly, and electronically, in Biocruces-Bizkaia Research Institute, stratified by Integrated Healthcare Organization (IHO) and in a 1: 1 ratio. Therefore, the participating centers will have access to EMAeHealth, while it will not be accessible to the control group centers.						
**INTERVENTIONS:**								
EMAeHealth +traditional ME (usual care)			←	—	—	—	→	
traditional ME (usual care)			←	—	—	—	→	
**ASSESSMENTS:**								
Baseline: Socio-demographic data and SF-36			**X**					
Outcomes: SF-36, clinical outcomes, self-efficacy for labor, self-efficacy for breastfeeding, health service use				**X**	**X**	**X**	**X**	**X**
Predictive, modifying and confounding variables			**X**	**X**	**X**	**X**	**X**	**X**

For peer review only

Patient information sheet for clinical trial and consent

Patient Information Sheet

For years we have been trying to improve the initiatives we carry out at Osakidetza to improve care for women during pregnancy and postpartum. We are therefore contacting you to ask for your consent to participate in a study your health center and midwife are collaborating in.

Invitation to participate and description of the procedure

Your health center and particularly your midwife are actively collaborating with the Bizkaia Primary Care Research Unit, part of the Biocruces-Bizkaia institute, to promote maternal education that is better adapted to the needs of women today. As a result, our research team is developing a website to support Maternal Education and we want to know how effective it is, so we would like to ask for your participation.

This website will offer rigorous information, selected by health professionals, on topics of interest throughout the period of pregnancy, childbirth and postpartum. It will enable women to access their clinical data securely, and will contain and offer resources at key moments in the maternity/paternity process. It will inform and support women in leading a healthy lifestyle, helping in the development of a birth plan that is adapted to each woman, advising on the appropriate choice at the onset of childbirth and offering care in the first days postpartum.

The website will be available to some randomly selected health centers, while others will not have this resource. We are doing this to compare the results on the health of women who use this resource (which you access with a password provided by your midwife) with those of women who do not have access to it.

Your participation in the study, regardless of your reference health center (with or without the website) will consist of filling in a series of sociodemographic questionnaires (e.g. age, level of studies, profession) and clinical questions (such as quality of life related to health or perception of self-efficacy during the birth process and breastfeeding), and it will also involve giving us permission to collect data from your medical records related to your pregnancy, childbirth and postpartum history.

The questionnaires must be completed at the beginning of pregnancy (approximately week 8), at weeks 24 and 34-36, and at weeks 1 and 6 postpartum. You fill it in online; your midwife will ask you for your mobile phone number and/or email address, and you will be sent a link to access the questionnaires. You will be able to complete them on a mobile phone, tablet or computer.

If you decide that you wish to participate, we will ask you to sign the attached consent form, which will be filed with the rest of the study documents.

Data processing and protection

The data that we collect via your mobile phone will be processed in an anonymized form, since it will be accessed with a numerical code, and confidentiality will be guaranteed at all times. The data collected for the study will be identified by this code, so that it does not include information that can identify you, and only your study doctor/researchers will be able to relate this data to you and your medical records. Therefore, your identity will not be disclosed to any person,

For peer review only

except in cases of medical emergency or legal requirement. The processing, communication and transfer of personal data of all participants will comply with the provisions of the law.

This study complies with the provisions of REGULATION (EU) 2016/679 OF THE EUROPEAN PARLIAMENT AND OF THE COUNCIL of 27 April 2016 on the protection of natural persons with regard to the processing of personal data and the free movement of such data, and of Organic Law 3/2018, of 5 December 2018 on the Protection of Personal Data and guarantee of digital rights that repeals Organic Law 15/1999 of 5 December 1999, on the protection of personal data. Personal data will be processed by the Basque Health Service, Osakidetza. No data will be transferred to third parties, except under legal obligation. The patient will be informed that they have the right of access, rectification, deletion of their data, and limitation or opposition to its use. In addition, further information on data protection can be found at the following website: http://www.osakidetza.euskadi.eus/protecciondatos

Benefits and risks

Participation in the study is entirely altruistic and carries no risk to you. The only cost that can be incurred is the time dedicated to answering the questions in the questionnaires. You are not required to answer questions that you consider to be private.

In return, you will be collaborating with research dedicated to the evaluation and improvement of the health service.

Voluntary participation

Your participation in the study is entirely voluntary.

Right to withdraw from the study

You have the right to withdraw from the study at any time without consequence to you.

Who to contact about the study

You can contact the Lead Researcher, Isabel Artieta, on tel. 946007711 if you need any clarification or want to resolve any doubts regarding the study.

The Coordinating Center for the study is the Bizkaia Primary Care Research Unit, which is part of Biocruces-Bizkaia and is located in Building 3, C/Plaza de Cruces, s/n. 48903 Barakaldo (Bizkaia). Tel. 946006637.

This study has been supervised and approved by the Basque Clinical Research Ethics Committee. (CEIm)

DECLARATION OF CONSENT

Study Title: “**Effectiveness and usability of a website for the self-management of women's health needs during pregnancy, childbirth and the postpartum period: EMAeHealth**”

Centre: **Bizkaia Primary Care Research Unit (Biocruces-Bizkaia Institute)**

For peer review only

I (name and surname) declare that

....

I have read the information sheet given to me.

I was able to ask questions about the study.

I have received enough information about the study.

I understand that in case of doubt I can go to the lead researcher:

Isabel Artieta. Zuazo Health Center (Barakaldo). Tel. 946007711

I understand that my participation is voluntary and that at any time, without explanation and without any impact on my medical care, I can withdraw from the study.

I freely give my consent to participate in the study.

DATE: PARTICIPANT'S SIGNATURE

The data collected will be added to a computer database in anonymized form, and be used to evaluate the research. This study complies with the provisions of REGULATION (EU) 2016/679 OF THE EUROPEAN PARLIAMENT AND OF THE COUNCIL of 27 April 2016 on the protection of natural persons with regard to the processing of personal data and the free movement of such data, and of Organic Law 3/2018, of 5 December 2018 on the Protection of Personal Data and guarantee of digital rights that repeals Organic Law 15/1999 of 5 December 1999, on the protection of personal data. Personal data will be processed by the Basque Health Service, Osakidetza. No data will be transferred to third parties, except under legal obligation. The patient will be informed that they have the right of access, rectification, deletion of their data, and limitation or opposition to its use. In addition, further information on data protection can be found at the following website: http://www.osakidetza.euskadi.eus/protecciondatos

THIS STUDY HAS BEEN EVALUATED AND APPROVED BY THE BASQUE CLINICAL RESEARCH ETHICS COMMITTEE (CEIm)

For peer review only

Focus groups of patients and focus groups of professionals and consent

Patient Information Sheet

In Osakidetza, we are trying to improve care for women during pregnancy and postpartum, and we would like your participation in a focus group in order to **get your opinion about the EMAeHealth website** that we have developed.

Invitation to participate in a focus group and description of the procedure

Your health center and particularly your midwife, together with researchers from the Bizkaia Primary Care Research Unit (Biocruces-Bizkaia), are looking into how to promote care that is better adapted to the needs of women today. To achieve this goal, as you know, a website called EMAeHealth has been designed.

As we would like to get a better idea of the opinions of the users about this new resource for Maternal Education - in terms of its ease of use, appropriateness and effectiveness - we would like to invite you to attend a focus group to explore these issues in depth. This participation is voluntary and only means attending a focus group meeting once. This meeting will be held on xxxxx (date), at xxxx (address) and will last approximately 90 minutes. The discussion will be moderated by two researchers from the Research Unit (xx and xx) and will be attended by other web users (between 6 and 8 of them) from different health centers, to talk about the topics under discussion.

Data processing and protection

The conversation generated by the group will be recorded entirely in audio to facilitate its subsequent transcription and analysis, with the collaboration of the qualitative studies research company xxx, which complies with the relevant legislation on data protection and is based in the Basque Country. We assure you that your opinions will be anonymized and that the confidentiality of the data collected will be guaranteed at all times. The audio files of the recordings and their corresponding transcripts (with names previously removed) will be stored in the computers of the Research Unit, access to which is restricted to researchers and

For peer review only

password-protected. Before the start of the recording, each participant will sign a consent form to authorize their participation in the study.

This study complies with the provisions of REGULATION (EU) 2016/679 OF THE EUROPEAN PARLIAMENT AND OF THE COUNCIL of 27 April 2016 on the protection of natural persons with regard to the processing of personal data and the free movement of such data, and of Organic Law 3/2018, of 5 December 2018 on the Protection of Personal Data and guarantee of digital rights that repeals Organic Law 15/1999 of 5 December 1999, on the protection of personal data. Personal data will be processed by the Basque Health Service, Osakidetza. No data will be transferred to third parties, except under legal obligation. The patient will be informed that they have the right of access, rectification, deletion of their data, and limitation or opposition to its use. In addition, further information on data protection can be found at the following website: http://www.osakidetza.euskadi.eus/protecciondatos

Benefits and risks

During the focus group discussion, the moderators will explain in detail what the technique involves, and you will be able to ask any questions or raise doubts throughout the entire session to make sure that you feel comfortable in that situation. You will also be given all kinds of opportunities to express your opinions freely and voluntarily, guaranteeing you an atmosphere of respect and confidentiality at all times. The main benefit of this participation is sharing your experience about the website, reflecting on ways to improve it and adapt it to the objective of promoting Maternal Education to meet your needs, thus contributing to the improvement of our health system.

Moreover, the moderators will take measures to minimize the impact of any potential risk that you or other participants may perceive or feel during the discussion in the group (such as fatigue, discomfort with any questions or conflict between participants). For example, you can take breaks, remain silent, state that you wish not to continue in the study, request more information about the objectives and procedures of the study and so on.

We remain at your entire disposal to offer any clarifications and answer your questions. If you need more details, you can contact the lead researcher of the study or the Bizkaia Primary Care Research Unit:

- **Isabel Artieta. Zuazo Health Center (Barakaldo). Tel. 946007711**
- **Bizkaia Primary Care Research Unit (Biocruces-Bizkaia). Tel. 946006637.** (Maite Espinosa)

For peer review only

Healthcare Professional Information Sheet

For years we have been involved in improving the initiatives we carry out in Osakidetza to improve care for women during pregnancy and postpartum. Therefore, we are contacting you to request your participation in a focus group, in order to **get your opinion about the EMAeHealth website** that we are developing.

Invitation to participate in a focus group and description of the procedure

We work with the Bizkaia Primary Care Research Unit (UIAPB) - Biocruces Bizkaia, in the development of strategies to promote Maternal Education that is better adapted to the needs of women today. To achieve this goal, as you know a website, EMAeHealth, has been designed, which we intend to subject to a constant process of improvement and updating.

As we would like to hear the opinions of the professionals about this new resource for supporting maternal education - in terms of its ease of use, suitability and effectiveness - we would like to invite you to attend a group conversation, or focus group, to analyze these issues in depth. Participation is voluntary and only requires you to attend once. This meeting will be held on xxxxx (date), at xxxx (address) and will last approximately 90 minutes. The conversation will be moderated by two researchers from the Research Unit (xx and xx) and will be attended by other professional users of the web (between 6 and 8 people), from different health centers, to talk about the topics under discussion.

Data processing and protection

Following the methodology of a focus group, the conversation generated by the group will be recorded entirely in audio to facilitate its subsequent transcription and analysis. In this task, the qualitative studies company xxx will collaborate with the UIAPB researchers. The company XXX (description) meets all the applicable requirements in terms of quality and data protection, and is based in the Basque Country, at (address xxx). The opinions of the participants will be processed in anonymized form and the confidentiality of the data collected will be guaranteed at all times. The audio files of the recordings and their corresponding transcripts (previously anonymized) will be stored on the computers of the Research Unit,

For peer review only

access to which is restricted to researchers and password-protected. Before the start of the recording, each participant will sign a consent form to authorize their participation in the study.

This study complies with the provisions of REGULATION (EU) 2016/679 OF THE EUROPEAN PARLIAMENT AND OF THE COUNCIL of 27 April 2016 on the protection of natural persons with regard to the processing of personal data and the free movement of such data, and of Organic Law 3/2018, of 5 December 2018 on the Protection of Personal Data and guarantee of digital rights that repeals Organic Law 15/1999 of 5 December 1999, on the protection of personal data. Personal data will be processed by the Basque Health Service, Osakidetza. No data will be transferred to third parties, except under legal obligation. The patient will be informed that they have the right of access, rectification, deletion of their data, and limitation or opposition to its use. In addition, further information on data protection can be found at the following website: http://www.osakidetza.euskadi.eus/protecciondatos

Benefits and risks

At the beginning of the session, the moderators will explain in detail what the technique involves and you will be able to clarify any doubts about the interview. Likewise, the participants will be able to express their opinions freely, in a climate of respect and confidentiality. Participation in the group is entirely altruistic, the main benefit being the possibility of sharing your experience about the website with other colleagues, reflecting on ways to improve it and adapt it to the objective of promoting Maternal Education that is more appropriate to the needs of women today.

Moreover, the moderators will take measures to minimize the impact of any potential risk that you may perceive during the development of the focus group (such as fatigue, discomfort with any questions or conflict between participants). You can withdraw your participation if you wish at any time, even during the interview itself. We remain at your entire disposal to offer any clarification or to answer your questions. If you need more information, you can contact the lead researcher of the study or the Bizkaia Primary Care Research Unit:

- **Isabel Artieta. Zuazo Health Center (Barakaldo). Tel. 946007711**
- **Bizkaia Primary Care Research Unit (Biocruces-Bizkaia). Tel. 946006637.** (Maite Espinosa)

Thank you very much.

For peer review only

DECLARATION OF CONSENT

Study Title: “**Effectiveness and usability of a website for the self-management of women's health needs during pregnancy, childbirth and the postpartum period: EMAeHealth**”

Centre: **Bizkaia Primary Care Research Unit (Biocruces-Bizkaia Institute)**

I (name and surname) declare that

....

I have read the information sheet given to me.
I was able to ask questions about the study.
I have received enough information about the study.
I know that in case of doubt I can go to the lead researcher:

Isabel Artieta. Zuazo Health Center (Barakaldo). Phone 946007711

I understand that my participation in the focus group is voluntary and that I can leave the meeting at any time, without explanation and without repercussion.
I freely give my consent for the meeting to be audio recorded and for the content of the meeting, including my contributions, to be transcribed in anonymized form for later analysis and research purposes.

DATE: PARTICIPANT’S SIGNATURE.

The data collected will be added to a computer database in anonymized form, and be used to evaluate the research. This study complies with the provisions of REGULATION (EU) 2016/679 OF THE EUROPEAN PARLIAMENT AND OF THE COUNCIL of 27 April 2016 on the protection of natural persons with regard to the processing of personal data and the free movement of such data, and of Organic Law 3/2018, of 5 December 2018 on the Protection of Personal Data and guarantee of digital rights that repeals Organic Law 15/1999 of 5 December 1999, on the protection of personal data. Personal data will be processed by the Basque Health Service, Osakidetza. No data will be transferred to third parties, except under legal obligation. The patient will be informed that they have the right of access, rectification, deletion of their data, and limitation or opposition to its use. In addition, further information on data protection can be found at the following website: http://www.osakidetza.euskadi.eus/protecciondatos

THIS STUDY HAS BEEN EVALUATED AND APPROVED BY THE BASQUE CLINICAL RESEARCH ETHICS COMMITTEE

For peer review only

SPIRIT 2013 Checklist: Recommended items to address in a clinical trial protocol and related documents*

Section/item	Item No	Description
**Administrative information**		
Title	1	**EMAeHealth, digital tool for the self-management of women's health needs during pregnancy, childbirth and the puerperium. Study protocol for an effectiveness-implementation hybrid design**. (Title page. Pag.1)
Trial registration	2a	ClinicalTrials.gov ID: NCT04937049
	2b	
Protocol version	3	Manuscript BMJ17-03-2022-v.2_EN.docx
Funding	4	Footnotes section. Funding section. Page 18.
Roles and responsibilities	5a	Footnotes section. Page 18 (Contributors)
	5b	Footnotes section. Page 18 (Funding)
	5c	Footnotes section. Page 18 (Disclaimer)
	5d	Footnotes section. Page 18
**Introduction**		
Background and rationale	6a	Introduction section. Page 3, 4 and 5.
	6b	Methods and analysis section. Page 9
Objectives	7	Aims section. Page 6
Trial design	8	Methods and analysis section. Page 6,7
**Methods: Participants, interventions, and outcomes**		
Study setting	9	Methods section. Pages 7 and 8.
Eligibility criteria	10	Methods section. Pages 7 (eligible centers) and page 8 (eligible patients)

For peer review only - http://bmjopen.bmj.com/site/about/guidelines.xhtml

Interventions	11a	Background page 4 (last paragraph), page 5 (first paragraph) Methods section, page 9 (Intervention centers, Control centers)
	11b	N/A
	11c	N/A
	11d	N/A
Outcomes	12	Methods section. Outcomes (pages 9,10,11)
Participant timeline	13	Methods section (Page 7, paragraph 3),Outcomes (page10, paragraph 2) and figure 2
Sample size	14	Methods section. Statistical methods: Study size page 12.
Recruitment	15	Methods section. Figure 1 and pages 7, 8, and 9.
**Methods: Assignment of interventions (for controlled trials)**		
Allocation:		
Sequence generation	16a	Methods section. *Random assignment of centers* (page 8)
Allocation concealment mechanism	16b	Methods section. *Random assignment of centers* (pages 8)
Implementation	16c	Methods section. Random assignment of centers (pages 8, 2nd paragraph, 4th), *Intervention and control centers* (page 8 ,9)
Blinding (masking)	17a	Methods section. Statistical methods: EMAeHealth *Clinical Effectiveness Results* page 11 (paragraph 3)
	17b	N/A
**Methods: Data collection, management, and analysis**		
Data collection methods	18a	Methods section. Outcomes (page 9, paragraph 3, page 10 2nd paragraph3)
	18b	N/A
Data management	19	Methods section. Outcomes (pages 10, paragraph 1 and 3)
Statistical methods	20a	Methods section. Statistical methods (pages 11)
	20b	Methods section. Statistical methods (pages 11)
	20c	Methods section. Statistical methods (pages 11)
**Methods: Monitoring**		

For peer review only - http://bmjopen.bmj.com/site/about/guidelines.xhtml

Data monitoring	21a	The research team will oversee and manage the project. A Trial Monitoring Committee has been established and contains a representative group of the researchers (Midwifes, Quality management expert, statistic, psychologist) who are responsible for the conduct of the trial and monitoring the study. There is not competing interest.
	21b	The trial Monitoring Committee will contact the midwives every fortnight to promote capturing the sample and gradually obtaining information on the intervention process. Bimonthly reports will be prepared on progress. Biannual meetings will be held for quality control and ongoing training.
Harms	22	N/A
Auditing	23	Annual reports will be prepared for the ethics committee, the Biocruces-Bizkaia clinical research institute and the Carlos III Health Institute
**Ethics and dissemination**		
Research ethics approval	24	See Ethics and dissemination, page 13, Ethics approval page 19(in Footnotes).
Protocol amendments	25	No important protocol modifications are foreseen because this is the final version of the protocol approved by the corresponding Ethics committee.
Consent or assent	26a	Methods section page 9, paragraph 1,2
	26b	N/A
Confidentiality	27	Methods: Outcomes 1st paragraph.
Declaration of interests	28	Footnotes section: Conflicts of interest page 18.
Access to data	29	The study is ongoing. The datasets that we will use and/or analysed during the study will be available from the corresponding author on reasonable request.
Ancillary and post-trial care	30	N/A
Dissemination policy	31a	The results of the study will be published in open access scientific journals and scientific and professional conferences and meetings. Patient associations will also be informed.

For peer review only

	31b	We will follow the recommendations of ICMJE Authorship eligibility guidelines.
	31c	N/A
**Appendices**		
Informed consent materials	32	Model consent form and other related documentation given to participants and authorised surrogates are available as a supplementary file
Biological specimens	33	N/A

*It is strongly recommended that this checklist be read in conjunction with the SPIRIT 2013 Explanation & Elaboration for important clarification on the items. Amendments to the protocol should be tracked and dated. The SPIRIT checklist is copyrighted by the SPIRIT Group under the Creative Commons "Attribution-NonCommercial-NoDerivs 3.0 Unported" license.

For peer review only

BMJ Open

EMAeHealth, a digital tool for the self-management of women's health needs during pregnancy, childbirth and the puerperium: protocol for a hybrid effectiveness-implementation study.

Journal:	*BMJ Open*
Manuscript ID	bmjopen-2021-055031.R2
Article Type:	Protocol
Date Submitted by the Author:	09-May-2022
Complete List of Authors:	Espinosa Cifuentes, Maite; Osakidetza-Basque Health Service, Primary Care Research Unit of Biscay. Healthcare Subdirectorate.; Biocruces Bizkaia Health Research Institute, Primary Health Care researcher<br>Artieta-Pinedo, Isabel; Osakidetza-Basque Health Service, Zuazo Health Centre, Barakaldo, (Bizkaia); Biocruces Bizkaia Health Research Institute, Associate Professor of the School of Nursing, University of the Basque Country, Leioa, Bizkaia, Spain.<br>Paz-pascual, Carmen; Osakidetza-Basque Health Service, Sestao Primary Care Health Centre, Bizkaia; Biocruces Bizkaia Health Research Institute, Lecturer in the Midwifery Training Unit of the Basque Country, Bilbao, Bizkaia, Spain.<br>Bully-Garay, Paola; Biocruces Bizkaia Health Research Institute, Member of the Research Group on Primary health care, prevention and chronic diseases. Statistics<br>García-Alvarez, Arturo; Osakidetza-Basque Health Service, Primary Care Research Unit of Biscay. Healthcare Subdirectorate.; Biocruces Bizkaia Health Research Institute<br>Group, emaQ ; Osakidetza-Basque Health Service; Biocruces Bizkaia Health Research Institute, Member of the Research Group on Primary health care, prevention and chronic diseases.
**Primary Subject Heading**:	Medical education and training
Secondary Subject Heading:	Health services research
Keywords:	Telemedicine < BIOTECHNOLOGY & BIOINFORMATICS, EDUCATION & TRAINING (see Medical Education & Training), Quality in health care < HEALTH SERVICES ADMINISTRATION & MANAGEMENT, Maternal medicine < OBSTETRICS, PRIMARY CARE

SCHOLARONE™
Manuscripts

Manuscript BMJ open

Title page

Title: **EMAeHealth, a digital tool for the self-management of women's health needs during pregnancy, childbirth and the puerperium: protocol for a hybrid effectiveness-implementation study.**

Full names and institutional addresses of all authors:

Maite Espinosa. (*1,2*) *

Isabel Artieta-Pinedo. (*1,2,4)*

Carmen Paz-Pascual. (*1,2,5*)

Paola Bully Garay. (*2*)

Arturo García-Alvarez. (1,2)

ema-Q Group (*3*)

***Correspondence to** Maite Espinosa. maite.espinosacifuentes@osakidetza.eus

1. Osakidetza-Basque Health Service.
2. BiocrucesBizkaia Health Research Institute (Members of the Research Group on Primary health care, prevention and chronic diseases)
3. Ema-Q Group Affiliations: Osakidetza-Basque Health Service, Biocruces-Bizkaia Health Research Institute, Basque Country University, Labal University of Canada, Cochrane
4. Primary Care Midwife Zuazo Health Centre, Barakaldo, (Bizkaia); and Associate Professor of the School of Nursing, University of the Basque Country, Leioa, Bizkaia, Spain.
5. Primary Care Midwife in Sestao Health Centre, Bizkaia; and Lecturer in the Midwifery Training Unit of the Basque Country, Bilbao, Bizkaia, Spain.

Isabel Artieta-Pinedo: mariaisabel.artietapinedo@osakidetza.eus
Carmen Paz-Pascual: mariacarmen.pazpascual@osakidetza.eus
Paola Bully: paola.bully01@gmail.com
Arturo García-Alvarez: arturo.garciaalvarez@osakidetza.eus
Consortia: Ema-Q Group
Ema-Q group is a multidisciplinary group made up mainly of Basque Health service midwives, as well as other medical professionals, psychologists, quality technicians and research experts: Itziar Estalella (IE), Mª José Trincado (MJT), Inés Cabeza (IC), Mari Pierre Gagnon (MPG), Ana Fernández (AF), Gorane Lozano (GL), Gemma Villanueva (GV), Jesús Sánchez (JS), Amaia Maquibar (AM), David Moreno (DM), Catalina Legarra (CL), Maria Jesús Mulas (MJM), Mónica Blas (MB), Pilar Amorrortu (PA), Sonia Alvarez (SA) and Covadonga Pérez (CP).

For peer review only - http://bmjopen.bmj.com/site/about/guidelines.xhtml

Abstract

Introduction: The EHealth can help the user of health services to take a more active role in decision-making and serve the professional to guide the patient in this process. A digital tool has been designed to support Maternal Education (ME), organized into four areas: 1) an Information area, 2) Communication area, 3) Health self-management area; 4) a Clinical data area. The main objective of the study is to evaluate the effectiveness of the EMAeHealth digital tool, and its usability and acceptability under routine conditions.

Methods and analysis: Hybrid implementation-effectiveness design: 1) A cluster-randomized, prospective, longitudinal, multicenter clinical trial to evaluate the effectiveness of EMAeHealth in -a) improving health-related quality of life (primary outcome), b) improving self-efficacy for labor and childbirth and self-efficacy in breastfeeding, and c) reducing the number of visits to the obstetric emergency services and health center in situations of "non-pathological pregnancy", "false labor" and "non-pathological puerperium". The EMAeHealth intervention plus usual care will be compared to receiving only usual care, which includes traditional ME. N= 1080 participants, 540 for each study arm. Two measurements will be made throughout the pregnancy and three in the first 16 weeks postpartum. 2) A mixed method study to evaluate the usability and acceptability of the tool, barriers and facilitators for its use, and implementation in our health system: Focus groups (women, professionals and agents involved) and a quantitative analysis of implementation indicators. Analysis: By intention to treat, using mixed models taking into account the hierarchical structure of the data, and per protocol to evaluate the effectiveness of the express use of the digital tool.

Ethics and dissemination: Clinical Research Ethics Committee of Euskadi, Spain, (Ref: PI2020044) approved this study. The results from will be actively disseminated through manuscript publications and conference presentations.

Trial registration number: NCT04937049

For peer review only

Strengths and limitations of this study

- EMAeHealth is the prototype of a digital tool that supports decision making during pregnancy and postpartum. One strength is that it has been designed through Collaborative Action Research and is intended to be a complement to Maternal Education.
- A hybrid efficacy-implementation design will allow the effectiveness of EMAeHealth to be evaluated while collecting data on the implementation of the tool in "real-world" conditions.

For peer review only - http://bmjopen.bmj.com/site/about/guidelines.xhtml

- Both patients and professionals have participated in the previous Collaborative Action Research and will be involved in the evaluation of the tool and in its implementation.
- One of the main strengths of the efficacy-implementation study is that it will result in an effective tool that will be perfectly adapted to the needs of the target population.
- A weakness is that the results might not be directly extrapolable to a different context and it would be necessary to adapt the tool to the context where it will be implemented.

Introduction

Need to adapt Maternal Education to the current context

Antenatal education classes, including ’childbirth education programs’, ’prenatal classes’, and antenatal groups, are attended by an important percentage of pregnant women worldwide. (1,2,3). Both the high attendance of maternal education (ME) classes, and the fact that they take place at a time of transition, when readiness for change is higher, suggest that ME can be seen as a valuable opportunity [1,2]. However, the benefits of these programs to participants and their newborn infants remain unclear, and classes typically have not based on the expressed needs of attendees, but rather on the messages that the educators themselves believe they should impart [1]. It may be that studies aimed at evaluating the effectiveness of ME have tended to have methodological weaknesses, such as small sample sizes, lack of control for variables that could affect the delivery process, and consideration of only part of the delivery process [1]. Nevertheless, it has also been found in our setting that EM classes have not been associated with an improvement in most birth variables, either physically or psychologically, and have even increased birth anxiety in foreign women [3]; in this study it was concluded that a new approach to them would be necessary. Other authors have come to the same conclusion, in light of their results [4,5].

In order to establish a feasible and effective perinatal education programme, tailored to our setting, the team - following guidance published by the UK Medical Research Council for the development and evaluation of complex interventions in primary care - undertook: (a) a qualitative study with 30 women exploring their needs during pregnancy and postpartum; (b) two literature reviews on

For peer review only - http://bmjopen.bmj.com/site/about/guidelines.xhtml

women's needs at these times and theoretical models of health education; and (c) seven discussion and consensus sessions with an expert panel of midwives, gynaecologists, paediatricians, paediatric and postpartum nurses [6, 7]. The aim was to involve women and professionals in the process of designing the new ME. Subsequently, a study was conducted to establish priorities with respect to maternal education using the Delphi technique and the nominal group approach. The objective of this study was to identify and prioritise the most important issues in maternal education in order to set goals and evaluate their achievement, i.e., to "make ME evaluable". [8].

The needs expressed by women in our population changed throughout pregnancy: Initially they needed information to know that "everything was going well"; next, they needed more emotional support, to feel confident and self-reliant in dealing with their fears about childbirth and childcare; they needed more postpartum support and less pressure regarding breastfeeding. They also preferred a comprehensive perinatal education programme - covering pregnancy, postpartum, breastfeeding and parenting - rather than just prenatal; and one that was more participatory and flexible and allowed for greater partner involvement. These were the results of the study with focus groups of women[6].

Accordingly, ME was redefined as a complex intervention, which should extend from the beginning of pregnancy to the end of the puerperium, focusing on the needs of women and with the involvement of multiple health professionals. It would be an intervention whose general objective should be to enable women in making appropriate decisions to: (a) improve their health level and that of their family, (b) choose and responsibly face their birth and parenting model, and (c) maintain family and social support networks. The new model of ME includes multiple interventions capable of responding to the health needs of each woman at different moments of the childbearing process [7, 8].

Design and relevance of an eHealth tool to support Maternal Education: EMAeHealth

For peer review only - http://bmjopen.bmj.com/site/about/guidelines.xhtml

EHealth interventions are becoming increasingly important and frequent [9]. EHealth can help the user of health services to take a more active role in decision-making and serve the professional to guide the patient in this process [10]. It can also contribute, at organizational level, in a change towards a model that includes the patient's voice to a greater extent [11].

In addition, young women from all social strata consistently use the Internet as a source of information about their health [12,13,14,15]. The information available on the websites they consult is positively correlated with the decisions they make about their health during pregnancy and postpartum [12,16]. Pregnant and postpartum women use the Internet because it offers immediate, detailed, entertaining, personalised or practical information [13,17].

However, the lack of participation of health professionals in integrating health care and new technologies is felt [12, 18], since the currently available range of information is not always reliable [19] or contextualized [20]. Since the origin of a website has a direct effect on reliability, the participation of health professionals in the use, advice and generation of new technologies is essential [15, 21].

This team has designed a prototype of digital tool called EMAeHealth involving healthcare professionals and patients, using a collaborative research process [21]. The website, adapted to our context of implementation, is organized into 4 areas: 1) an Information area, which is open to the public, provide information based on evidence and is permanently updated by healthcare professionals; 2) a Communication area, which allows women to contact others in a similar situation, through forums or conversations, or consult their own midwife 3) a Health Self-management area, which has valid and reliable self-assessment instruments for checking or reflecting on their own health needs, and decision support tools such as the detection of the onset of labour or the detection of warning signs and symptoms in the postpartum; 4) a Clinical data area, which allows women to have keep their clinical data, to add the most recent, and share data with other professionals if they wish, under conditions of security. The tool is conceived as a complement that can reinforce Maternal Education; with resources that facilitate its accessibility, immediacy and continuity, as well

For peer review only - http://bmjopen.bmj.com/site/about/guidelines.xhtml

For peer review only

as improving its results. Its incorporation into the Osakidetza-Basque Health Service corporate website will encourage its adoption by patients and professionals, as well as its safe use. The usual phases for the development of a digital tool are analysis, development, testing and production. The prototype is the result of the analysis, a proposal of the architecture and technical requirements that the digital tool must have in order to respond to the users´ needs. This is followed by development -or programming- and testing, which are carried out almost simultaneously, to detect and solve errors. Once the tool is ready, it is deployed in a production environment, making it available to all users of the system, in our case the Osakidetza-Basque Health Service corporate website.

Study of the effectiveness of the tool while we are collecting useful information for its implementation in routine conditions

The evaluation of the effectiveness of digital tools aimed at health education during pregnancy is scarce and has had controversial results [22,23]: the quality, reliability and effectiveness of current pregnancy apps is yet to be determined [15, 22,23], consequently, such studies to evaluate effectiveness are absolutely essential, even more so if the digital tool comes from the healthcare system. In addition, there are barriers and facilitators that modify both the use of digital technology and its effectiveness in health care, where further study is needed [13,16,17,24, 25, 26]. Fortunately, there are hybrid designs that allow testing of the clinical intervention and, at the same time, during the trial, gathering information on its application and/or its potential for application in real-world conditions [27]. There are also frameworks, such as RE-AIM and CFIR, which can help in the task of studying the degree of adoption of an innovation and guide in the data collection, analysis and interpretation of barriers and facilitators to implementation. [28, 29]

Aims

The main objective of the study is twofold, on the one hand to evaluate the effectiveness of the EMAeHealth digital tool and its components (Information Area, Communication Area, Health Self-

For peer review only - http://bmjopen.bmj.com/site/about/guidelines.xhtml

Management Area and Clinical Data Area), and on the other to assess the usability and acceptability of the tool under routine conditions.

The following are proposed as *specific objectives*:

- To develop, test and deploy the EMAeHealth tool [21], with the structure described above, capable of offering: information, responses via algorithm, links to available resources, secure data archiving and monitoring, connection between various systems and the possibility of data analysis establishing continuous feedback and readjustment of the information presented. To provide it with content related to pregnancy, childbirth and postpartum.
- To evaluate the effectiveness of the tool in 1) improving health-related quality of life, 2) improving self-efficacy for labour and childbirth and self-efficacy in breastfeeding, and 3) reducing the number of visits to the obstetric emergency services and health center in situations of “non-pathological pregnancy”, “false labour” and “non-pathological puerperium”.
- To evaluate the usability and acceptability of the tool by women and professionals, and any barriers to its implementation.
- To estimate the differential impact that EMAeHealth could have, based on sociodemographic and/or population characteristics.

Methods and analysis

This is an *Effectiveness-implementation hybrid design type I* [26], which combines 1) a multicenter, longitudinal, prospective, cluster-randomized clinical trial to evaluate the effectiveness of EMAeHealth and 2) a mixed-method study to evaluate the usability and acceptability of the tool, barriers and facilitators for its use and implementation in our health system. The mixed method study is made up of a qualitative study by focus groups (women, professionals and agents involved) and a quantitative analysis of implementation indicators. **(Fig. 1)**

For peer review only - http://bmjopen.bmj.com/site/about/guidelines.xhtml

For the effectiveness study, we have chosen a randomized cluster design to avoid, among other issues, contamination bias among women from the same center, since they are likely to coincide, for example, in Maternal Education classes. In addition, as the intervention has been designed for shared decision-making, it did not seem appropriate for the same midwife to have women belonging to control and intervention.

The study will be conducted between 2021 and 2023, with the first year dedicated to the development of the EMAeHealth digital tool. The general structure of the tool is described in the introduction. In the development of the prototype, the International Patient Decision Aid Standards (IPDAS) were applied, using a collaborative action research process, involving experts and patients, with qualitative research techniques, as well as focus groups, prioritization and consensus techniques [21]. At this time, we are finalizing content editing and the software development and testing. We expect to start recruitment in January 2022, until June 2022. The participants will be followed-up for a year, the period of time that the intervention lasts. Final months of 2023 will be dedicated to analysis and diffusion of results.

Regarding the *study population*, all OSAKIDETZA-Basque Health Service public primary care centers that have midwives interested in optimizing their clinical practice by adopting innovations will be eligible. In order to consider the *center eligible*, the requirement will be that at least 50% of the midwives belonging to the center have signed a collaboration agreement and the approval of the Head of the Primary Care Center (HPCC).

Osakidetza-Basque Health System is the public healthcare system of the Basque Country, a region located in the north of Spain. Osakidetza was created by the Health Department of the Basque Government in 1983. All the public hospitals and primary care centers of the Basque Region are under this organization, structured into several Integrated Health Organizations (IHO) spread throughout the Basque country. More than 30,000 professionals work for Osakidetza, which could be considered the largest organization in the Basque Country.

For peer review only - http://bmjopen.bmj.com/site/about/guidelines.xhtml

For the study of clinical effectiveness, an active surveillance system will be established to identify *eligible patients*, who are Spanish-speaking pregnant women who attend their first consultation with the midwife between week 6 and 10 of gestation. Regarding the clinical profile of them, they are low-risk pregnant women. The midwives participating in the study will be in charge of recruiting them consecutively until they have the necessary sample from among the people exposed and not exposed to the EMAeHealth tool. **(Fig. 2)**

Regarding the mixed-method study, to evaluate the quantitative implementation indicators, all pregnant women who have seen their midwives at the start of gestation at the health centers will be included for one year after the implementation of EMAeHealth. The same goes for the *qualitative* evaluation, through focus groups of women and professionals from the health centers *(model consent form as a supplementary file)*.

Random assignment of centers:

The eligible centers will be randomized centrally, blindly, and electronically, in Biocruces-Bizkaia Research Institute, stratified by Integrated Healthcare Organization (IHO) and in a 1: 1 ratio. Therefore, the intervention allocated centres will have access to EMAeHealth, while it will not be accessible to the control group centers. Integrated Health Organizations are groups of health centers within the same geographic area, with similar characteristics and population, under the direction of the same hospital. Therefore, we assume that there are no pre-existing differences in the quality of care provided between centers that belong to the same integrated health organization.

Intervention centers

All the patients in these centers, in the first consultation with the midwife, will be informed that EMAeHealth is available to them, and the content of the tool will be told to them. In addition, they will be invited to participate in the study of its clinical effectiveness. If the answer is affirmative, they must sign the informed consent and will be provided with a link to the forms (baseline measurements and questionnaires for each measurement) to fill in electronically. Participants sign an informed

For peer review only - http://bmjopen.bmj.com/site/about/guidelines.xhtml

consent approved by the Ethics Committee and can leave the study whenever they want *(model consent form as a supplementary file)*.

Control centers

Patients in these centers will not have EMAehealth. They will receive, like the intervention group, the usual care and the possibility of attending ME sessions, but not EMAeHealth. After being informed of the clinical effectiveness study, they will be asked for consent to participate *(see supplementary file).* Women who sign the informed consent for participation in the study will be provided with the link to the forms for each measurement.

Outcomes

Access to EMAeHealth by women and professionals during the study period will be through access codes, which will allow the research team to track the use made by users of the tool while ensuring the confidentiality of the data. It also makes it possible, under safe conditions, to associate this use with clinical variables from medical records and with the record of the forms completed electronically at each measurement.

Results of clinical effectiveness: The primary outcome is change in Health-related quality of life, measured with the Spanish version of the SF-36 questionnaire [30]. The secondary variables are the number and reason for visits to the obstetric emergency service and to the health center for non-pathological pregnancy situations, and the labor stage at the time of going to the hospital for suspected labor onset (measurement is carried out using the Bishop test /dilation in cm, effacement and breaking of waters (YES/NO)) . As indirect indicators of autonomy in managing their own health and well-being, self-efficacy for labor and childbirth and self-efficacy in breastfeeding will be measured using the Spanish version of Lowe's CBSEI scales [31] and the Breastfeeding Self-Efficacy Scale - Short Form[32], respectively.

For peer review only - http://bmjopen.bmj.com/site/about/guidelines.xhtml

Measurements of these variables will be performed at weeks 8 and 37 of pregnancy, and at weeks 1, 6, and 16 postpartum. The contact with the woman and the response to the questionnaires will be carried out electronically (via mobile phone).

Quality of life (QoL) is a parameter that has been defined and recently taken into account as a health indicator by WHO [33]. The concept of QoL could be defined as an individual's assessment of his or her own state in relation to his or her needs [33], so it seems appropriate that it should be the primary outcome measure. The Spanish version of the SF-36 questionnaire has been used to assess the health-related quality of life perceived by pregnant women in several studies [33; 34], showing good psychometric characteristics. The SF-36 questionnaire is made up of 8 domains, of which 4 are oriented towards physical aspects and another 4 towards mental aspects of health-related quality of life. In addition, two summaries are made of this physical and mental component, which explain between 80 and 85% of the variance of the 8 domains [30]. The scores of the questionnaire are transformed on a scale from 0 to 100 points; the higher the score, the better the perception of health-related quality of life. A difference of 5 points in the domain score, or between 2 and 3 points in the summary score is considered clinically relevant. [30]

Self-efficacy is one of the key factors influencing women's confidence and ability to cope with childbirth and the Childbirth Self-Efficacy Inventory has been shown to be a valid and reliable instrument to measure maternal confidence in childbirth. The Spanish version of the Childbirth Self-Efficacy Inventory shows adequate psychometric properties (i.e. internal consistency and validity). Moreover, self-efficacy is a variable sensitive to external modifications, so we believe it may be suitable as one of the secondary measures to assess the effectiveness of using EMAeHealth.

Similarly, breastfeeding self-efficacy is a mother's confidence in her ability to breastfeed and is highly predictive of breastfeeding behaviors. The Spanish version of the BSES-SF can be considered a valid and reliable measure of maternal breastfeeding self-efficacy.

For peer review only - http://bmjopen.bmj.com/site/about/guidelines.xhtml

Indicators of the implementation process: Qualitative indicators: Usability and acceptability of the tool by patients and professionals, as well as barriers and facilitators for its use and adoption in the health system. The qualitative evaluation will be carried out through focus groups of women and professionals, between 6 and 8 per group and as many as necessary until the discourse is saturated [35]. A semi-structured script will be developed for the sessions, based on the theoretical constructs of the CFIR [28], which will also serve as the basis for the analysis. The CFIR includes 37 constructs within 5 main domains: intervention characteristics, outer setting, inner setting, individual characteristics and implementation process. Overall, the CFIR aims to help identify potential factors (i.e. barriers and facilitators) that are believed to influence implementation. Sessions will be audio-recorded and moderated by an expert in qualitative techniques while an assistant will take notes. Later the sessions will be transcribed and analysed. Quantitative indicators: adoption and frequency of use of EMAeHealth and its areas according to stratification profiles and correlates with other variables (indicators: patients exposed to the digital tool, by center and group; women with access to EMAeHealth who access the tool once; frequency of use of the tool, frequency of use of the different functions of the tool, etc.).

Predictive, modifying and confounding variables: *Patients*: Age, Parity, Nationality, Race, Social class, Educational level, Marital status, Work situation, Distance to hospital, Spontaneous pregnancy vs assisted reproductive technique, Obstetric history, and other clinical characteristics. *Professionals*: Sex, age, length of time in the position, type of contract, profile of adoption of innovations by professionals, measured by means of a questionnaire by Borracci et al., 2013. *Health centers*: reference IHOs, number of midwives and participation rate, rural or urban environment, and population served. **(Fig. 3)**

Statistical methods

EMAeHealth *Clinical Effectiveness Results*: quantitative comparisons will be made between intervention and control centers, with an intention-to-treat approach. Mixed models (linear or generalized as appropriate to the variable under study), that take into account the hierarchical

For peer review only - http://bmjopen.bmj.com/site/about/guidelines.xhtml

structure of the data (patients grouped by midwives, midwives grouped by center), will be used and 95% confidence intervals will be calculated. Likewise, per-protocol analysis will be carried out, in which the effectiveness of the express use of both the tool in general and in particular of some of its components will be evaluated, adjusting for possible confounding factors. Statistical analysis will be carried out by a professional who is not part of the research team.

Implementation process: For the *qualitative* evaluation of the tool and its implementation, the analysis of the focus groups, facilitated by the ATLAS.ti software program, will be structured in three phases: a deductive analysis to determine which CFIR constructs influence its use in real life, followed by an inductive analysis to identify others not included in the CFIR framework that could influence its use, and a third validation phase using triangulation. The analysis results report will be provided to each patient and professional, who will have the opportunity to provide feedback to the team of analysts with their comments ("response validity").

For the *quantitative* evaluation, the distribution of the indicators associated with the use and scope of the tool as well as its components will be described. Measures of central tendency, dispersion and proportions will be calculated, with 95% confidence intervals, and the relationship between the indicators and the predictor/confounding variables will be analyzed using mixed models.

Study size

For the clinical effectiveness study, 18 centers will need to recruit 30 patients each, in each arm of the study (N=1080). This will make it possible to detect a difference of 2 points as significant in the mental component of the SF-36 test, with an alpha error of less than 5% and power greater than 80%. A 10-point standard deviation is assumed for the mental SF-36 and an intra-cluster correlation of 0.01.

The calculation took into account the cluster design of the study and it was carried out using the National Institute of Health GRT sample size calculator* which assumes that the analysis will employ mixed-model regression methods.

For peer review only - http://bmjopen.bmj.com/site/about/guidelines.xhtml

* Research Methods Resources: National Institutes of Health. [Accessed 02/01/2020]. Available from: https://researchmethodsresources.nih.gov/grt-calculator

Patient involvement

In previous studies, multidisciplinary groups of midwives, nurses, paediatricians, epidemiologists, psychologists and pregnant and postpartum women were involved in the description and prioritisation of ME needs, as well as in the redesign of the ME and the design of an EMAeHealth tool to complement it [3, 6, 7, 8, 8, 15, 21]. In the present study, patients will be involved in testing the tool under construction, assessing its usability, acceptability and evaluating barriers and facilitators for its implementation in "real-world conditions". In addition, women will participate as study subjects in the evaluation of the effectiveness of the digital tool.

Ethics and dissemination

The study protocol has been reviewed and ethics approval obtained from the by the Clinical Research Ethics Committee of Euskadi, Spain in November 2020 (Ref: PI2020044).-This approval covers all centers included in the study. Participation in this study will be voluntary; individual written (or online) consent will be sought from all participants. Each participant will receive a study information sheet that outlines the project and expectations in plain language

Manuscripts will be published in open access journals and findings will be further disseminated through local and international Conferences.

Acknowledgements

We would like to acknowledge the collaboration of those interested in the design of the EMAeHealth tool, the grant received by the Carlos III Health Institute in collaboration with the ISCII- Subdirectorate General for Evaluation and Promotion of Research (European Regional Development Fund-FEDER) and the support of Biocruces Bizkaia Health Research Institute. We also want to thank the invaluable work of Katy Ryan, who has been responsible for translating the manuscript into English.

For peer review only - http://bmjopen.bmj.com/site/about/guidelines.xhtml

For peer review only

References

1. Gagnon AJ, Sandall J. Individual or group antenatal education for childbirth or parenthood, or both. Cochrane Database of Systematic Reviews 2007, Issue 3. Art. No.: CD002869. DOI: 10.1002/14651858.CD002869.pub2.
2. https://www.nice.org.uk › Default › ***Antenatal*** Education Evidence Review Final.docx
3. Artieta-Pinedo I, Paz-Pascual C, Grandes G, et al. The benefits of antenatal education for the childbirth process in Spain. *Nurs Res*. 2010;59(3):194-202. doi:10.1097/NNR.0b013e3181dbbb4e.
4. Billingham K. Preparing for parenthood: the role of antenatal education. Community Practitioner : the Journal of the Community Practitioners' & Health Visitors' Association. 2011 May;84(5):36-38.
5. McMillan, Anita & Barlow, Jane & Redshaw, Margaret & Britain, Great. (2009). Birth and beyond: a review of the evidence about antenatal education. https://www.researchgate.net/publication/258369289_Birth_and_beyond_a_review_of_the_evidence_about_antenatal_education
6. Paz Pascual C, Artieta Pinedo I, Grandes G, Espinosa M, Gaminde Inda I, Payo Gordon J. [Perceived needs of women regarding maternity. Qualitative study to redesign maternal education]. Aten Primaria. 2016;48(10):657-664. doi:10.1016/j.aprim.2015.12.004
7. Artieta-Pinedo I, Paz-Pascual C, Grandes G, Espinosa M. Framework for the establishment of a feasible, tailored and effective perinatal education programme. *BMC Pregnancy Childbirth*. 2017;17(1):58. Published 2017 Feb 8. doi:10.1186/s12884-017-1234-7
8. Paz-Pascual C, Artieta-Pinedo I, Grandes G; ema.Q Group. Consensus on priorities in maternal education: results of Delphi and nominal group technique approaches. *BMC Pregnancy Childbirth*. 2019;19(1):264. Published 2019 Jul 24. doi:10.1186/s12884-019-2382-8
9. World Health Organization. Digital Health. https://www.who.int/ehealth/en/

For peer review only - http://bmjopen.bmj.com/site/about/guidelines.xhtml

10. Ruiz-Azarola A, Perestelo-Pérez L. Participación ciudadana en salud: formación y toma de decisiones compartida. Informe SESPAS 2012 [Citizens' participation in health: education and shared decision-making. SESPAS Report 2012]. *Gac Sanit*. 2012;26 Suppl 1:158-161. doi:10.1016/j.gaceta.2011.10.005

11. Mora J et al, (2019) Cómo avanzar con el paciente. Modelo afectivo efectivo. The Institute for Health and Strategy S.L. (Si-Health). https://www.sihealth.es/es/publicaciones-eventos/publicaciones/

12. Bert F, Gualano MR, Brusaferro S, et al. Pregnancy e-health: a multicenter Italian cross-sectional study on Internet use and decision-making among pregnant women. *J Epidemiol Community Health*. 2013;67(12):1013-1018. doi:10.1136/jech-2013-202584

13. Hughson JP, Daly JO, Woodward-Kron R, Hajek J, Story D. The Rise of Pregnancy Apps and the Implications for Culturally and Linguistically Diverse Women: Narrative Review. *JMIR Mhealth Uhealth*. 2018;6(11):e189. Published 2018 Nov 16. doi:10.2196/mhealth.9119

14. O'Higgins A, Murphy OC, Egan A, Mullaney L, Sheehan S, Turner MJ. The use of digital media by women using the maternity services in a developed country. Ir Med J. 2014 Nov-Dec;107(10):313-5. PMID: 25556255.

15. Artieta-Pinedo I, Paz-Pascual C, Grandes G, Villanueva G; Ema Q Group. An evaluation of Spanish and English on-line information sources regarding pregnancy, birth and the postnatal period. *Midwifery*. 2018;58:19-26. doi:10.1016/j.midw.2017.12.002

16. Wallwiener S, Müller M, Doster A, et al. Pregnancy eHealth and mHealth: user proportions and characteristics of pregnant women using Web-based information sources-a cross-sectional study. *Arch Gynecol Obstet*. 2016;294(5):937-944. doi:10.1007/s00404-016-4093-y

17. Lupton D. The use and value of digital media for information about pregnancy and early motherhood: a focus group study. *BMC Pregnancy Childbirth*. 2016;16(1):171. Published 2016 Jul 19. doi:10.1186/s12884-016-0971-3

For peer review only - http://bmjopen.bmj.com/site/about/guidelines.xhtml

18. Rodger D, Skuse A, Wilmore M, et al. Pregnant women's use of information and communications technologies to access pregnancy-related health information in South Australia. *Aust J Prim Health*. 2013;19(4):308-312. doi:10.1071/PY13029

19. Brown HM, Bucher T, Collins CE, Rollo ME. A review of pregnancy iPhone apps assessing their quality, inclusion of behaviour change techniques, and nutrition information. *Matern Child Nutr*. 2019;15(3):e12768. doi:10.1111/mcn.12768

20. Kraschnewski JL, Chuang CH, Poole ES, et al. Paging "Dr. Google": does technology fill the gap created by the prenatal care visit structure? Qualitative focus group study with pregnant women. *J Med Internet Res*. 2014;16(6):e147. Published 2014 Jun 3. doi:10.2196/jmir.3385

21. Artieta-Pinedo I, Paz-Pascual C, Bully P, Espinosa M, EmaQ Group. Design of the Maternal Website EMAeHealth That Supports Decision-Making During Pregnancy and in the Postpartum Period: Collaborative Action Research Study.JMIR Form Res 2021; 5(8):e28855.URL: https://formative.jmir.org/2021/8/e28855. DOI: 10.2196/28855

22. Overdijkink SB, Velu AV, Rosman AN, van Beukering MD, Kok M, Steegers-Theunissen RP. The Usability and Effectiveness of Mobile Health Technology-Based Lifestyle and Medical Intervention Apps Supporting Health Care During Pregnancy: Systematic Review. *JMIR Mhealth Uhealth*. 2018;6(4):e109. Published 2018 Apr 24. doi:10.2196/mhealth.8834

23. Roch G, Borgès Da Silva R, de Montigny F, et al. Impacts of online and group perinatal education: a mixed methods study protocol for the optimization of perinatal health services. *BMC Health Serv Res*. 2018;18(1):382. Published 2018 May 29. doi:10.1186/s12913-018-3204-9

24. Berland GK, Elliott MN, Morales LS, et al. Health information on the Internet: accessibility, quality, and readability in English and Spanish. *JAMA*. 2001;285(20):2612-2621. doi:10.1001/jama.285.20.2612

25. Risling T, Martinez J, Young J, Thorp-Froslie N. Evaluating Patient Empowerment in Association With eHealth Technology: Scoping Review. *J Med Internet Res*. 2017;19(9):e329. Published 2017 Sep 29. doi:10.2196/jmir.7809

For peer review only - http://bmjopen.bmj.com/site/about/guidelines.xhtml

26. Leonardsen AL, Hardeland C, Helgesen AK, Grøndahl VA. Patient experiences with technology enabled care across healthcare settings- a systematic review. BMC Health Serv Res. 2020;20(1):779. Published 2020 Aug 24. doi:10.1186/s12913-020-05633-4

27. Curran GM, Bauer M, Mittman B, Pyne JM, Stetler C. Effectiveness-implementation hybrid designs: combining elements of clinical effectiveness and implementation research to enhance public health impact. Med Care. 2012;50(3):217-226. doi:10.1097/MLR.0b013e3182408812

28. Damschroder LJ, Aron DC, Keith RE, Kirsh SR, Alexander JA, Lowery JC. Fostering implementation of health services research findings into practice: a consolidated framework for advancing implementation science. Implement Sci. 2009;4:50. Published 2009 Aug 7. doi:10.1186/1748-5908-4-50

29. Gaglio B, Shoup JA, Glasgow RE. The RE-AIM framework: a systematic review of use over time. *Am J Public Health*. 2013;103(6):e38-e46. doi:10.2105/AJPH.2013.301299

30. Vilagut G, Ferrer M, Rajmil L, et al. El Cuestionario de Salud SF-36 español: una década de experiencia y nuevos desarrollos [The Spanish version of the Short Form 36 Health Survey: a decade of experience and new developments]. *Gac Sanit*. 2005;19(2):135-150. doi:10.1157/13074369

31. Cunqueiro MJ, Comeche MI, Docampo D. Childbirth Self-Efficacy Inventory: psychometric testing of the Spanish version. *J Adv Nurs*. 2009;65(12):2710-2718. doi:10.1111/j.1365-2648.2009.05161.x

32. Oliver-Roig A, d'Anglade-González ML, García-García B, Silva-Tubio JR, Richart-Martínez M, Dennis CL. The Spanish version of the Breastfeeding Self-Efficacy Scale-Short Form: reliability and validity assessment. *Int J Nurs Stud*. 2012;49(2):169-173. doi:10.1016/j.ijnurstu.2011.08.005

33. Martínez-Galiano JM, Hernández-Martínez A, Rodríguez-Almagro J, Delgado-Rodríguez M. Quality of Life of Women after Giving Birth: Associated Factors Related with the Birth Process. *J Clin Med*. 2019;8(3):324. Published 2019 Mar 7. doi:10.3390/jcm8030324

For peer review only - http://bmjopen.bmj.com/site/about/guidelines.xhtml

34. Schubert KO, Air T, Clark SR, et al. Trajectories of anxiety and health related quality of life during pregnancy. *PLoS One*. 2017;12(7):e0181149. Published 2017 Jul 24. doi:10.1371/journal.pone.0181149

35. Hennink MM, Kaiser BN, Weber MB. What Influences Saturation? Estimating Sample Sizes in Focus Group Research. *Qual Health Res*. 2019;29(10):1483-1496. doi:10.1177/1049732318821692

Footnotes

Author statement: IA, CP, PB and ME designed the EMAeHealth program, with EMA-Q Group´s collaboration, AG has collaborated on the statistics and on the revision of the article. ; All of them have been involved in the design of the protocol and have collaborated in writing the manuscript; all authors read, contributed, and finally approved the manuscript. *Contributors*: Arturo García (statistics) and patients involved.

Funding: This study has been funded by the Carlos III National Health Institute, (grant number: PI20/00899.), (Co-funded by European Regional Development Fund "A way to make Europe ").

Disclaimer: The funding source has not been involved in study design, collection, analysis and interpretation of data, writing of the report or in the decision to submit the article for publication.

Conflicts of interests: The authors declare that they have no competing interests.

Patient involvement: See details in the methods and analysis section.

Patient consent for publication: Not required

Ethics approval: Clinical Research Ethics Committee of Euskadi (Spain), in November 2020 (Ref: PI2020044)

Provenance and peer review: Not commissioned; externally peer reviewed

For peer review only - http://bmjopen.bmj.com/site/about/guidelines.xhtml

Data sharing statement: Access to the data is feasible by requesting permission from the main researcher, Isabel Artieta. The protocol and the research results will be published. Access to data, ICF, etc. are available by requesting it from the main researcher, Isabel Artieta. (MARIAISABEL.ARTIETAPINEDO@osakidetza.eus)

Word count: 4263 (References and footnotes not included), 5800 (Total)

Figure legends

Figure 1. EMAeHealth design description

Figure 2. EMAeHealth effectiveness flow chart

Figure 3. SPIRIT-Timetable

For peer review only

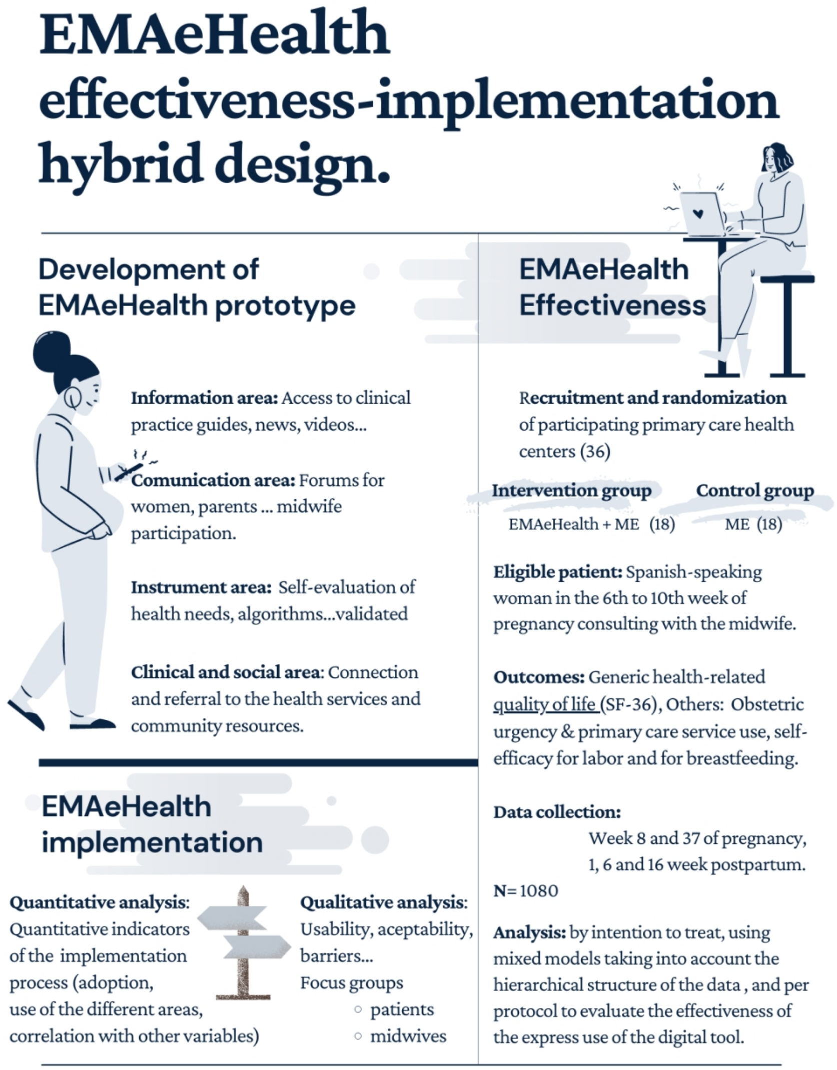

Figure 1. EMAeHealth design description

1060x1500mm (38 x 38 DPI)

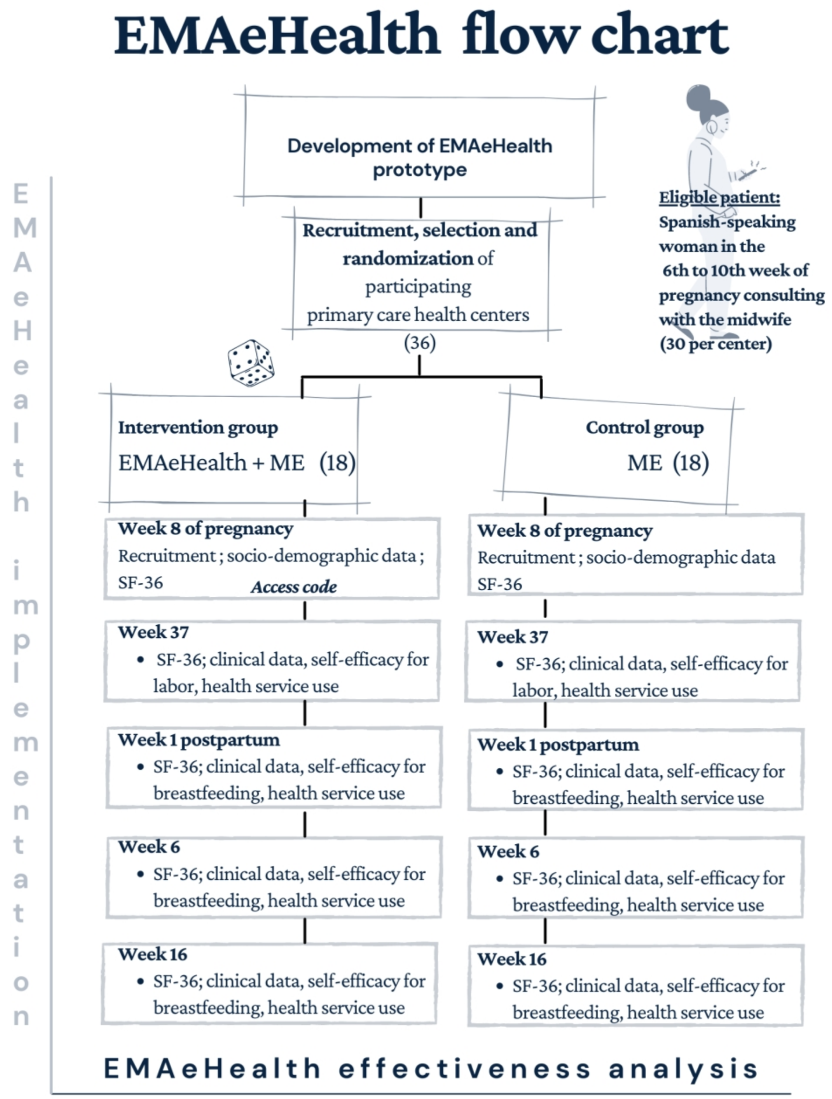

Figure 2. EMAeHealth effectiveness flow chart

1060x1500mm (38 x 38 DPI)

EMAeHealth Spirit timeline table.

	STUDY PERIOD							
	Enrolment	Allocation	Post-allocation					Close-out
**TIMEPOINT****	***January 2022-june 2022***	**0**	***week 8 of pregnancy***	***week 37 of pregnancy***	***Week 1 postpartum***	***Week 6 postpartum***	***Week 16 postpartum***	***June 2023-Dec 2023***
**ENROLMENT:**	January 2022-june 2022. Enrollment of participants will be after allocation of the centers. It will be done, for both arms, consecutively, until complete the sample (Total N=1080)							
**Eligibility screen**	Spanish-speaking woman around the 8th week of pregnancy consulting with the midwife of eligible centers.							
**Informed consent**	First consultation with the midwife between week around 8th week of gestation							
**Allocation**		The eligible health centers will be randomized centrally, blindly, and electronically, in Biocruces-Bizkaia Research Institute, stratified by Integrated Healthcare Organization (IHO) and in a 1: 1 ratio. Therefore, the participating centers will have access to EMAeHealth, while it will not be accessible to the control group centers.						
**INTERVENTIONS:**								
EMAeHealth +traditional ME (usual care)			←	—	—	—	→	
traditional ME (usual care)			←	—	—	—	→	
**ASSESSMENTS:**								
Baseline: Socio-demographic data and SF-36			**X**					
Outcomes: SF-36, clinical outcomes, self-efficacy for labor, self-efficacy for breastfeeding, health service use				**X**	**X**	**X**	**X**	**X**
Predictive, modifying and confounding variables			**X**	**X**	**X**	**X**	**X**	**X**

Patient information sheet for clinical trial and consent

Patient Information Sheet

For years we have been trying to improve the initiatives we carry out at Osakidetza to improve care for women during pregnancy and postpartum. We are therefore contacting you to ask for your consent to participate in a study your health center and midwife are collaborating in.

Invitation to participate and description of the procedure

Your health center and particularly your midwife are actively collaborating with the Bizkaia Primary Care Research Unit, part of the Biocruces-Bizkaia institute, to promote maternal education that is better adapted to the needs of women today. As a result, our research team is developing a website to support Maternal Education and we want to know how effective it is, so we would like to ask for your participation.

This website will offer rigorous information, selected by health professionals, on topics of interest throughout the period of pregnancy, childbirth and postpartum. It will enable women to access their clinical data securely, and will contain and offer resources at key moments in the maternity/paternity process. It will inform and support women in leading a healthy lifestyle, helping in the development of a birth plan that is adapted to each woman, advising on the appropriate choice at the onset of childbirth and offering care in the first days postpartum.

The website will be available to some randomly selected health centers, while others will not have this resource. We are doing this to compare the results on the health of women who use this resource (which you access with a password provided by your midwife) with those of women who do not have access to it.

Your participation in the study, regardless of your reference health center (with or without the website) will consist of filling in a series of sociodemographic questionnaires (e.g. age, level of studies, profession) and clinical questions (such as quality of life related to health or perception of self-efficacy during the birth process and breastfeeding), and it will also involve giving us permission to collect data from your medical records related to your pregnancy, childbirth and postpartum history.

The questionnaires must be completed at the beginning of pregnancy (approximately week 8), at weeks 24 and 34-36, and at weeks 1 and 6 postpartum. You fill it in online; your midwife will ask you for your mobile phone number and/or email address, and you will be sent a link to access the questionnaires. You will be able to complete them on a mobile phone, tablet or computer.

If you decide that you wish to participate, we will ask you to sign the attached consent form, which will be filed with the rest of the study documents.

Data processing and protection

The data that we collect via your mobile phone will be processed in an anonymized form, since it will be accessed with a numerical code, and confidentiality will be guaranteed at all times. The data collected for the study will be identified by this code, so that it does not include information that can identify you, and only your study doctor/researchers will be able to relate this data to you and your medical records. Therefore, your identity will not be disclosed to any person,

For peer review only

except in cases of medical emergency or legal requirement. The processing, communication and transfer of personal data of all participants will comply with the provisions of the law.

This study complies with the provisions of REGULATION (EU) 2016/679 OF THE EUROPEAN PARLIAMENT AND OF THE COUNCIL of 27 April 2016 on the protection of natural persons with regard to the processing of personal data and the free movement of such data, and of Organic Law 3/2018, of 5 December 2018 on the Protection of Personal Data and guarantee of digital rights that repeals Organic Law 15/1999 of 5 December 1999, on the protection of personal data. Personal data will be processed by the Basque Health Service, Osakidetza. No data will be transferred to third parties, except under legal obligation. The patient will be informed that they have the right of access, rectification, deletion of their data, and limitation or opposition to its use. In addition, further information on data protection can be found at the following website: http://www.osakidetza.euskadi.eus/protecciondatos

Benefits and risks

Participation in the study is entirely altruistic and carries no risk to you. The only cost that can be incurred is the time dedicated to answering the questions in the questionnaires. You are not required to answer questions that you consider to be private.

In return, you will be collaborating with research dedicated to the evaluation and improvement of the health service.

Voluntary participation

Your participation in the study is entirely voluntary.

Right to withdraw from the study

You have the right to withdraw from the study at any time without consequence to you.

Who to contact about the study

You can contact the Lead Researcher, Isabel Artieta, on tel. 946007711 if you need any clarification or want to resolve any doubts regarding the study.

The Coordinating Center for the study is the Bizkaia Primary Care Research Unit, which is part of Biocruces-Bizkaia and is located in Building 3, C/Plaza de Cruces, s/n. 48903 Barakaldo (Bizkaia). Tel. 946006637.

This study has been supervised and approved by the Basque Clinical Research Ethics Committee. (CEIm)

DECLARATION OF CONSENT

Study Title: “**Effectiveness and usability of a website for the self-management of women's health needs during pregnancy, childbirth and the postpartum period: EMAeHealth**”

Centre: **Bizkaia Primary Care Research Unit (Biocruces-Bizkaia Institute)**

For peer review only

I (name and surname) declare that

....

I have read the information sheet given to me.

I was able to ask questions about the study.

I have received enough information about the study.

I understand that in case of doubt I can go to the lead researcher:

Isabel Artieta. Zuazo Health Center (Barakaldo). Tel. 946007711

I understand that my participation is voluntary and that at any time, without explanation and without any impact on my medical care, I can withdraw from the study.

I freely give my consent to participate in the study.

DATE: PARTICIPANT'S SIGNATURE

The data collected will be added to a computer database in anonymized form, and be used to evaluate the research. This study complies with the provisions of REGULATION (EU) 2016/679 OF THE EUROPEAN PARLIAMENT AND OF THE COUNCIL of 27 April 2016 on the protection of natural persons with regard to the processing of personal data and the free movement of such data, and of Organic Law 3/2018, of 5 December 2018 on the Protection of Personal Data and guarantee of digital rights that repeals Organic Law 15/1999 of 5 December 1999, on the protection of personal data. Personal data will be processed by the Basque Health Service, Osakidetza. No data will be transferred to third parties, except under legal obligation. The patient will be informed that they have the right of access, rectification, deletion of their data, and limitation or opposition to its use. In addition, further information on data protection can be found at the following website: http://www.osakidetza.euskadi.eus/protecciondatos

THIS STUDY HAS BEEN EVALUATED AND APPROVED BY THE BASQUE CLINICAL RESEARCH ETHICS COMMITTEE (CEIm)

For peer review only

Focus groups of patients and focus groups of professionals and consent

Patient Information Sheet

In Osakidetza, we are trying to improve care for women during pregnancy and postpartum, and we would like your participation in a focus group in order to **get your opinion about the EMAeHealth website** that we have developed.

Invitation to participate in a focus group and description of the procedure

Your health center and particularly your midwife, together with researchers from the Bizkaia Primary Care Research Unit (Biocruces-Bizkaia), are looking into how to promote care that is better adapted to the needs of women today. To achieve this goal, as you know, a website called EMAeHealth has been designed.

As we would like to get a better idea of the opinions of the users about this new resource for Maternal Education - in terms of its ease of use, appropriateness and effectiveness - we would like to invite you to attend a focus group to explore these issues in depth. This participation is voluntary and only means attending a focus group meeting once. This meeting will be held on xxxxx (date), at xxxx (address) and will last approximately 90 minutes. The discussion will be moderated by two researchers from the Research Unit (xx and xx) and will be attended by other web users (between 6 and 8 of them) from different health centers, to talk about the topics under discussion.

Data processing and protection

The conversation generated by the group will be recorded entirely in audio to facilitate its subsequent transcription and analysis, with the collaboration of the qualitative studies research company xxx, which complies with the relevant legislation on data protection and is based in the Basque Country. We assure you that your opinions will be anonymized and that the confidentiality of the data collected will be guaranteed at all times. The audio files of the recordings and their corresponding transcripts (with names previously removed) will be stored in the computers of the Research Unit, access to which is restricted to researchers and

For peer review only

password-protected. Before the start of the recording, each participant will sign a consent form to authorize their participation in the study.

This study complies with the provisions of REGULATION (EU) 2016/679 OF THE EUROPEAN PARLIAMENT AND OF THE COUNCIL of 27 April 2016 on the protection of natural persons with regard to the processing of personal data and the free movement of such data, and of Organic Law 3/2018, of 5 December 2018 on the Protection of Personal Data and guarantee of digital rights that repeals Organic Law 15/1999 of 5 December 1999, on the protection of personal data. Personal data will be processed by the Basque Health Service, Osakidetza. No data will be transferred to third parties, except under legal obligation. The patient will be informed that they have the right of access, rectification, deletion of their data, and limitation or opposition to its use. In addition, further information on data protection can be found at the following website: http://www.osakidetza.euskadi.eus/protecciondatos

Benefits and risks

During the focus group discussion, the moderators will explain in detail what the technique involves, and you will be able to ask any questions or raise doubts throughout the entire session to make sure that you feel comfortable in that situation. You will also be given all kinds of opportunities to express your opinions freely and voluntarily, guaranteeing you an atmosphere of respect and confidentiality at all times. The main benefit of this participation is sharing your experience about the website, reflecting on ways to improve it and adapt it to the objective of promoting Maternal Education to meet your needs, thus contributing to the improvement of our health system.

Moreover, the moderators will take measures to minimize the impact of any potential risk that you or other participants may perceive or feel during the discussion in the group (such as fatigue, discomfort with any questions or conflict between participants). For example, you can take breaks, remain silent, state that you wish not to continue in the study, request more information about the objectives and procedures of the study and so on.

We remain at your entire disposal to offer any clarifications and answer your questions. If you need more details, you can contact the lead researcher of the study or the Bizkaia Primary Care Research Unit:

- **Isabel Artieta. Zuazo Health Center (Barakaldo). Tel. 946007711**
- **Bizkaia Primary Care Research Unit (Biocruces-Bizkaia). Tel. 946006637.** (Maite Espinosa)

For peer review only

Healthcare Professional Information Sheet

For years we have been involved in improving the initiatives we carry out in Osakidetza to improve care for women during pregnancy and postpartum. Therefore, we are contacting you to request your participation in a focus group, in order to **get your opinion about the EMAeHealth website** that we are developing.

Invitation to participate in a focus group and description of the procedure

We work with the Bizkaia Primary Care Research Unit (UIAPB) - Biocruces Bizkaia, in the development of strategies to promote Maternal Education that is better adapted to the needs of women today. To achieve this goal, as you know a website, EMAeHealth, has been designed, which we intend to subject to a constant process of improvement and updating.

As we would like to hear the opinions of the professionals about this new resource for supporting maternal education - in terms of its ease of use, suitability and effectiveness - we would like to invite you to attend a group conversation, or focus group, to analyze these issues in depth. Participation is voluntary and only requires you to attend once. This meeting will be held on xxxxx (date), at xxxx (address) and will last approximately 90 minutes. The conversation will be moderated by two researchers from the Research Unit (xx and xx) and will be attended by other professional users of the web (between 6 and 8 people), from different health centers, to talk about the topics under discussion.

Data processing and protection

Following the methodology of a focus group, the conversation generated by the group will be recorded entirely in audio to facilitate its subsequent transcription and analysis. In this task, the qualitative studies company xxx will collaborate with the UIAPB researchers. The company XXX (description) meets all the applicable requirements in terms of quality and data protection, and is based in the Basque Country, at (address xxx). The opinions of the participants will be processed in anonymized form and the confidentiality of the data collected will be guaranteed at all times. The audio files of the recordings and their corresponding transcripts (previously anonymized) will be stored on the computers of the Research Unit,

For peer review only

access to which is restricted to researchers and password-protected. Before the start of the recording, each participant will sign a consent form to authorize their participation in the study.

This study complies with the provisions of REGULATION (EU) 2016/679 OF THE EUROPEAN PARLIAMENT AND OF THE COUNCIL of 27 April 2016 on the protection of natural persons with regard to the processing of personal data and the free movement of such data, and of Organic Law 3/2018, of 5 December 2018 on the Protection of Personal Data and guarantee of digital rights that repeals Organic Law 15/1999 of 5 December 1999, on the protection of personal data. Personal data will be processed by the Basque Health Service, Osakidetza. No data will be transferred to third parties, except under legal obligation. The patient will be informed that they have the right of access, rectification, deletion of their data, and limitation or opposition to its use. In addition, further information on data protection can be found at the following website: http://www.osakidetza.euskadi.eus/protecciondatos

Benefits and risks

At the beginning of the session, the moderators will explain in detail what the technique involves and you will be able to clarify any doubts about the interview. Likewise, the participants will be able to express their opinions freely, in a climate of respect and confidentiality. Participation in the group is entirely altruistic, the main benefit being the possibility of sharing your experience about the website with other colleagues, reflecting on ways to improve it and adapt it to the objective of promoting Maternal Education that is more appropriate to the needs of women today.

Moreover, the moderators will take measures to minimize the impact of any potential risk that you may perceive during the development of the focus group (such as fatigue, discomfort with any questions or conflict between participants). You can withdraw your participation if you wish at any time, even during the interview itself. We remain at your entire disposal to offer any clarification or to answer your questions. If you need more information, you can contact the lead researcher of the study or the Bizkaia Primary Care Research Unit:

- **Isabel Artieta. Zuazo Health Center (Barakaldo). Tel. 946007711**
- **Bizkaia Primary Care Research Unit (Biocruces-Bizkaia). Tel. 946006637.** (Maite Espinosa)

Thank you very much.

For peer review only

DECLARATION OF CONSENT

Study Title: "**Effectiveness and usability of a website for the self-management of women's health needs during pregnancy, childbirth and the postpartum period: EMAeHealth**"

Centre: **Bizkaia Primary Care Research Unit (Biocruces-Bizkaia Institute)**

I (name and surname) declare that

....

I have read the information sheet given to me.
I was able to ask questions about the study.
I have received enough information about the study.
I know that in case of doubt I can go to the lead researcher:

Isabel Artieta. Zuazo Health Center (Barakaldo). Phone 946007711

I understand that my participation in the focus group is voluntary and that I can leave the meeting at any time, without explanation and without repercussion.
I freely give my consent for the meeting to be audio recorded and for the content of the meeting, including my contributions, to be transcribed in anonymized form for later analysis and research purposes.

DATE: PARTICIPANT'S SIGNATURE.

The data collected will be added to a computer database in anonymized form, and be used to evaluate the research. This study complies with the provisions of REGULATION (EU) 2016/679 OF THE EUROPEAN PARLIAMENT AND OF THE COUNCIL of 27 April 2016 on the protection of natural persons with regard to the processing of personal data and the free movement of such data, and of Organic Law 3/2018, of 5 December 2018 on the Protection of Personal Data and guarantee of digital rights that repeals Organic Law 15/1999 of 5 December 1999, on the protection of personal data. Personal data will be processed by the Basque Health Service, Osakidetza. No data will be transferred to third parties, except under legal obligation. The patient will be informed that they have the right of access, rectification, deletion of their data, and limitation or opposition to its use. In addition, further information on data protection can be found at the following website: http://www.osakidetza.euskadi.eus/protecciondatos

THIS STUDY HAS BEEN EVALUATED AND APPROVED BY THE BASQUE CLINICAL RESEARCH ETHICS COMMITTEE

For peer review only

STANDARD PROTOCOL ITEMS: RECOMMENDATIONS FOR INTERVENTIONAL TRIALS

SPIRIT 2013 Checklist: Recommended items to address in a clinical trial protocol and related documents*

Section/item	Item No	Description
**Administrative information**		
Title	1	**EMAeHealth, digital tool for the self-management of women's health needs during pregnancy, childbirth and the puerperium. Study protocol for an effectiveness-implementation hybrid design**. (Title page. Pag.1)
Trial registration	2a	ClinicalTrials.gov ID: NCT04937049
	2b	
Protocol version	3	Manuscript BMJ- 24-06-2021-v.1_EN.docx
Funding	4	Footnotes section. Funding section. Page 19.
Roles and responsibilities	5a	Footnotes section. Page 19 (Contributors)
	5b	Footnotes section. Page 19 (Funding)
	5c	Footnotes section. Page 19 (Disclaimer)
	5d	Footnotes section. Page 19
**Introduction**		
Background and rationale	6a	Introduction section. Page 3, 4, 5 and 6.
	6b	Methods and analysis section. Page 9 (Random assignment of the centers)
Objectives	7	Aims section. Pages 6 and 7
Trial design	8	Methods and analysis section. Page 7,8,9
**Methods: Participants, interventions, and outcomes**		
Study setting	9	Methods section. Pages 8 and 9.
Eligibility criteria	10	Methods section. Pages 8 (eligible centers) and page 9 (eligible patients)

For peer review only

Interventions	11a	Background page 5 (last paragraph) Methods section, page 9 (Intervention centers)
	11b	N/A
	11c	N/A
	11d	N/A
Outcomes	12	Methods section. Outcomes (pages 10,11,12)
Participant timeline	13	Methods section. Outcomes (page11) and figure 2
Sample size	14	Methods section. Statistical methods: Study size page 13.
Recruitment	15	Methods section. Figure 1 and pages 8, and 9.
**Methods: Assignment of interventions (for controlled trials)**		
Allocation:		
Sequence generation	16a	Methods section. *Random assignment of centers* (page 9)
Allocation concealment mechanism	16b	Methods section. *Random assignment of centers* (pages 9)
Implementation	16c	Methods section. Random assignment of centers (pages 9), *Participating centers*)
Blinding (masking)	17a	Methods section. Statistical methods: EMAeHealth *Clinical Effectiveness Results* page 12 (paragraph 1)
	17b	N/A
**Methods: Data collection, management, and analysis**		
Data collection methods	18a	Methods section. Outcomes (page 10, paragraph 1,2 and 3)
	18b	N/A
Data management	19	Methods section. Outcomes (pages 10)
Statistical methods	20a	Methods section. Statistical methods (pages 12)
	20b	Methods section. Statistical methods (pages 12)
	20c	Methods section. Statistical methods (pages 12)
**Methods: Monitoring**		

Data monitoring	21a	The research team will oversee and manage the project. A Trial Monitoring Committee has been established and contains a representative group of the researchers (Midwifes, Quality management expert, statistic, psychologist) who are responsible for the conduct of the trial and monitoring the study. There is not competing interest.
	21b	The trial Monitoring Committee will contact the midwives every fortnight to promote capturing the sample and gradually obtaining information on the intervention process. Bimonthly reports will be prepared on progress. Biannual meetings will be held for quality control and ongoing training.
Harms	22	N/A
Auditing	23	Annual reports will be prepared for the ethics committee, the Biocruces-Bizkaia clinical research institute and the Carlos III Health Institute
**Ethics and dissemination**		
Research ethics approval	24	See Ethics approval and consent to participate in Footnotes page 19.
Protocol amendments	25	No important protocol modifications are foreseen because because this is the final version of the protocol approved by the corresponding Ethics committee.
Consent or assent	26a	Methods section page 9 and 10
	26b	N/A
Confidentiality	27	Methods/ Outcomes 1st paragraph.
Declaration of interests	28	Footnotes section: Competing interest page 19.
Access to data	29	The study is ongoing. The datasets that we will use and/or analysed during the study will be available from the corresponding author on reasonable request.
Ancillary and post-trial care	30	N/A
Dissemination policy	31a	The results of the study will be published in open access scientific journals and scientific and professional conferences and meetings. Patient associations will also be informed.

For peer review only

	31b	We will follow the recommendations of ICMJE Authorship eligibility guidelines.
	31c	N/A
**Appendices**		
Informed consent materials	32	Model consent form and other related documentation given to participants and authorised surrogates are available as a supplementary file
Biological specimens	33	N/A

*It is strongly recommended that this checklist be read in conjunction with the SPIRIT 2013 Explanation & Elaboration for important clarification on the items. Amendments to the protocol should be tracked and dated. The SPIRIT checklist is copyrighted by the SPIRIT Group under the Creative Commons "Attribution-NonCommercial-NoDerivs 3.0 Unported" license.

For peer review only

BMJ Open

EMAeHealth, a digital tool for the self-management of women's health needs during pregnancy, childbirth and the puerperium: protocol for a hybrid effectiveness-implementation study.

Journal:	*BMJ Open*
Manuscript ID	bmjopen-2021-055031.R3
Article Type:	Protocol
Date Submitted by the Author:	27-Jun-2022
Complete List of Authors:	Espinosa Cifuentes, Maite; Osakidetza-Basque Health Service, Primary Care Research Unit of Biscay. Healthcare Subdirectorate.; Biocruces Bizkaia Health Research Institute, Primary Health Care researcher<br>Artieta-Pinedo, Isabel; Osakidetza-Basque Health Service, Zuazo Health Centre, Barakaldo, (Bizkaia); Biocruces Bizkaia Health Research Institute, Associate Professor of the School of Nursing, University of the Basque Country, Leioa, Bizkaia, Spain.<br>Paz-pascual, Carmen; Osakidetza-Basque Health Service, Sestao Primary Care Health Centre, Bizkaia; Biocruces Bizkaia Health Research Institute, Lecturer in the Midwifery Training Unit of the Basque Country, Bilbao, Bizkaia, Spain.<br>Bully-Garay, Paola; Biocruces Bizkaia Health Research Institute, Member of the Research Group on Primary health care, prevention and chronic diseases. Statistics<br>García-Alvarez, Arturo; Osakidetza-Basque Health Service, Primary Care Research Unit of Biscay. Healthcare Subdirectorate.; Biocruces Bizkaia Health Research Institute<br>Group, emaQ ; Osakidetza-Basque Health Service; Biocruces Bizkaia Health Research Institute, Member of the Research Group on Primary health care, prevention and chronic diseases.
**Primary Subject Heading**:	Medical education and training
Secondary Subject Heading:	Health services research
Keywords:	Telemedicine < BIOTECHNOLOGY & BIOINFORMATICS, EDUCATION & TRAINING (see Medical Education & Training), Quality in health care < HEALTH SERVICES ADMINISTRATION & MANAGEMENT, Maternal medicine < OBSTETRICS, PRIMARY CARE

SCHOLARONE™
Manuscripts

Manuscript BMJ open

Title page

Title: **EMAeHealth, a digital tool for the self-management of women's health needs during pregnancy, childbirth and the puerperium: protocol for a hybrid effectiveness-implementation study.**

Full names and institutional addresses of all authors:

Maite Espinosa. (*1,2*) *

Isabel Artieta-Pinedo. (*1,2,4)*

Carmen Paz-Pascual. (*1,2,5*)

Paola Bully Garay. (*2*)

Arturo García-Alvarez. (1,2)

ema-Q Group (*3*)

***Correspondence to** Maite Espinosa: maite.espinosacifuentes@osakidetza.eus

1. Osakidetza-Basque Health Service.
2. BiocrucesBizkaia Health Research Institute (Members of the Research Group on Primary health care, prevention and chronic diseases)
3. Ema-Q Group Affiliations: Osakidetza-Basque Health Service, Biocruces-Bizkaia Health Research Institute, Basque Country University, Labal University of Canada, Cochrane
4. Primary Care Midwife Zuazo Health Centre, Barakaldo, (Bizkaia); and Associate Professor of the School of Nursing, University of the Basque Country, Leioa, Bizkaia, Spain.
5. Primary Care Midwife in Sestao Health Centre, Bizkaia; and Lecturer in the Midwifery Training Unit of the Basque Country, Bilbao, Bizkaia, Spain.

Isabel Artieta-Pinedo: mariaisabel.artietapinedo@osakidetza.eus
Carmen Paz-Pascual: mariacarmen.pazpascual@osakidetza.eus
Paola Bully: paola.bully01@gmail.com
Arturo García-Alvarez: arturo.garciaalvarez@osakidetza.eus
Consortia: Ema-Q Group
Ema-Q group is a multidisciplinary group made up mainly of Basque Health service midwives, as well as other medical professionals, psychologists, quality technicians and research experts: Itziar Estalella (IE), Mª José Trincado (MJT), Inés Cabeza (IC), Mari Pierre Gagnon (MPG), Ana Fernández (AF), Gorane Lozano (GL), Gemma Villanueva (GV), Jesús Sánchez (JS), Amaia Maquibar (AM), David Moreno (DM), Catalina Legarra (CL), Maria Jesús Mulas (MJM), Mónica Blas (MB), Pilar Amorrortu (PA), Sonia Alvarez (SA) and Covadonga Pérez (CP).

For peer review only - http://bmjopen.bmj.com/site/about/guidelines.xhtml

Abstract

Introduction: EHealth can help health service users take a more active role in decision-making and help health professionals guide the patient in this process. A digital tool has been designed to support Maternal Education (ME), and it is organised into four areas: 1) Information, 2) Communication, 3) Health Self-Management, 4) Clinical Data. The main objective of the study is to evaluate the effectiveness of the EMAeHealth digital tool, and assess its usability and acceptability under routine conditions.

Methods and analysis: Hybrid implementation-effectiveness design: 1) A cluster randomised, prospective, longitudinal, multicentre clinical trial to evaluate the effectiveness of EMAeHealth in a) improving health-related quality of life (primary outcome), b) improving self-efficacy for labour and childbirth and self-efficacy in breastfeeding, and c) reducing the number of visits to the obstetric emergency services and health centre in situations of "non-pathological pregnancy", "false labour pains" and "non-pathological puerperium". The EMAeHealth intervention plus usual care will be compared to receiving only usual care, which includes traditional ME. N= 1080 participants, 540 for each study arm. Two measurements will be made throughout the pregnancy and three in the first 16 weeks postpartum. 2) A mixed method study to evaluate the usability and acceptability of the tool, barriers and facilitators for its use, and implementation in our health system: focus groups (women, professionals and agents involved) and a quantitative analysis of implementation indicators. Analysis: by intention to treat, using mixed models taking into account the hierarchical structure of the data, and per protocol to evaluate the effectiveness of the express use of the digital tool.

Ethics and dissemination: Clinical Research Ethics Committee of Euskadi, Spain, (Ref: PI2020044) approved this study. The results will be actively disseminated through manuscript publications and conference presentations.

Trial registration number: NCT04937049

Strengths and limitations of this study

- EMAeHealth is the prototype of a digital tool that supports decision-making during pregnancy and postpartum. One strength is that it has been designed through Collaborative Action Research and is intended to be a complement to Maternal Education.
- A hybrid efficacy-implementation design will allow the effectiveness of EMAeHealth to be evaluated while collecting data on the implementation of the tool in "real-world" conditions.

For peer review only - http://bmjopen.bmj.com/site/about/guidelines.xhtml

- Both patients and professionals have participated in the previous Collaborative Action Research and will be involved in the evaluation of the tool and in its implementation.
- One of the main strengths of the efficacy-implementation study is that it will result in an effective tool that will be perfectly adapted to the needs of the target population.
- A weakness is that it is possible the results cannot be directly extrapolated to a different context and it would be necessary to adapt the tool to the context where it will be implemented.

Introduction

Need to adapt Maternal Education to the current context

Antenatal education classes, including ’childbirth education programmes’, ’prenatal classes’, and antenatal groups, are attended by a large percentage of pregnant women worldwide. (1,2,3). Both the high attendance of maternal education (ME) classes, and the fact that they take place at a time of transition, when readiness for change is higher, suggest that ME can be a valuable window of opportunity [1,2]. However, the benefits of these programmes for participants and their newborn infants remain unclear, and classes are not normally based on the expressed needs of attendees, but rather on the messages that the educators themselves believe they should impart [1]. It may be that studies aimed at evaluating the effectiveness of ME have tended to have methodological weaknesses, such as small sample sizes, lack of control for variables that could affect the delivery process, and consideration of only part of the delivery process [1]. Whatever the case, it has also been found in our setting that ME classes have not been associated with an improvement in most birth variables, whether physical or psychological, and have even increased birth anxiety in foreign women [3]; in this study, it was concluded that a new approach to these classes would be necessary. Other authors have come to the same conclusion, in light of their results [4,5]. There is therefore a need to reorient ME by focusing on women's needs and to evaluate the effectiveness of any initiatives to improve ME.

In order to establish a feasible and effective perinatal education programme, tailored to our setting, the team – following guidance published by the UK Medical Research Council for the development

For peer review only - http://bmjopen.bmj.com/site/about/guidelines.xhtml

and evaluation of complex interventions in primary care – undertook: (a) a qualitative study with 30 women, exploring their needs during pregnancy and postpartum; (b) two literature reviews on women's needs at these times and theoretical models of health education; and (c) seven discussion and consensus sessions with an expert panel of midwives, gynaecologists, paediatricians, paediatric and postpartum nurses [6, 7]. The aim was to involve women and professionals in the process of designing the new ME. Subsequently, a study was conducted to establish priorities with respect to maternal education using the Delphi technique and the nominal group approach. The objective of this study was to identify and prioritise the most important issues in maternal education in order to set goals and evaluate their achievement, i.e., to "make ME evaluable". [8].

The needs expressed by women in our population changed throughout pregnancy: initially they needed information to know that "everything was going well"; next, they needed more emotional support, to feel confident and self-reliant in dealing with their fears about childbirth and childcare; and finally, they needed more postpartum support and less pressure regarding breastfeeding. They also preferred a comprehensive perinatal education programme – covering pregnancy, postpartum, breastfeeding and parenting – rather than just prenatal; and one that was more participatory and flexible and allowed for greater partner involvement. These were the results of the study with focus groups of women [6].

Accordingly, ME was redefined as a complex intervention, which should extend from the beginning of pregnancy to the end of the puerperium, focusing on the needs of women and with the involvement of multiple health professionals. It would be an intervention whose general objective should be to enable women to make appropriate decisions to: (a) improve their health level and that of their family, (b) choose and responsibly face their birth and parenting model, and (c) maintain family and social support networks. The new model of ME includes multiple interventions capable of responding to the health needs of each woman at different moments of the childbearing process [7, 8].

Design and relevance of an eHealth tool to support Maternal Education: EMAeHealth

For peer review only - http://bmjopen.bmj.com/site/about/guidelines.xhtml

EHealth interventions are becoming increasingly important and frequent [9]. EHealth can help the health service user to take a more active role in decision-making and enable the health professional to guide the patient in this process [10]. It can also contribute, at organisational level, to a more patient-centred model of healthcare [11].

In addition, young women from all social strata consistently use the Internet as a source of information about their health [12,13,14,15]. The information available on the websites they consult is positively correlated with the decisions they make about their health during pregnancy and postpartum [12,16]. Pregnant and postpartum women use the Internet because it offers immediate, detailed, entertaining, personalised or practical information [13,17].

However, the lack of participation of health professionals in integrating health care and new technologies is felt [12, 18], since the currently available range of information is not always reliable [19] or contextualised [20]. As the origin of a website has a direct effect on reliability, the participation of health professionals in the use, advice and generation of new technologies is essential [15, 21].

This team has designed a prototype digital tool called EMAeHealth involving healthcare professionals and patients, using a collaborative research process [21]. The website, adapted to our context of implementation, is organised into 4 areas: 1) an Information area, which is open to the public, provides information based on evidence and is permanently updated by healthcare professionals; 2) a Communication area, which allows women to contact others in a similar situation, through forums or conversations, or consult their own midwife 3) a Health Self-Management area, which has valid and reliable self-assessment instruments for checking or reflecting on their own health needs, and decision support tools such as the detection of the onset of labour or the detection of warning signs and symptoms in the postpartum; and 4) a Clinical Data area, which allows women to keep their clinical data, to add the most recent, and share data with other professionals if they wish, under secure conditions. The tool is conceived as a complement that can reinforce Maternal Education; with resources that facilitate its accessibility, immediacy and continuity, as well as improving its

For peer review only - http://bmjopen.bmj.com/site/about/guidelines.xhtml

results. Its incorporation into the Osakidetza-Basque Health Service corporate website will encourage its adoption by patients and professionals, as well as its safe use. The usual phases for the development of a digital tool are analysis, development, testing and production. The prototype is the result of the analysis, a proposal of the architecture and technical requirements that the digital tool must have in order to respond to the users' needs. This is followed by development – or programming – and testing, which are carried out almost simultaneously, to detect and solve errors. Once the tool is ready, it is deployed in a production environment, making it available to all users of the system, in our case the Osakidetza-Basque Health Service corporate website.

Study of the effectiveness of the tool while we are collecting useful information for its implementation in routine conditions

The evaluation of the effectiveness of digital tools aimed at health education during pregnancy is scarce and has had controversial results [22,23]: the quality, reliability and effectiveness of current pregnancy apps is yet to be determined [15, 22,23], and consequently such studies to evaluate effectiveness are absolutely essential, especially if the digital tool comes from the healthcare system. In addition, there are barriers and facilitators that modify both the use of digital technology and its effectiveness in health care, where further study is needed [13,16,17,24, 25, 26]. Fortunately, there are hybrid designs that enable testing of the clinical intervention and, at the same time, during the trial, gathering information on its application and/or its potential for application in real-world conditions [27]. There are also frameworks, such as the RE-AIM Planning and Evaluation Framework and Consolidated Framework for Implementation Research (CFIR), which can help in the task of studying the degree of adoption of an innovation, and guide in the data collection, analysis and interpretation of barriers and facilitators to implementation. [28, 29]. Therefore, we conducted this study to find out if complementing traditional perinatal care with a tool - designed through a collaborative action-research process - translates into better health outcomes for women than those obtained with traditional care alone. In addition, we want to know if this type of tool is useful and acceptable to its users in real-life conditions.

For peer review only - http://bmjopen.bmj.com/site/about/guidelines.xhtml

Objectives

The main objective of the study is twofold; on the one hand to evaluate the effectiveness of the EMAeHealth digital tool and its components (Information Area, Communication Area, Health Self-Management Area and Clinical Data Area), and on the other to assess the usability and acceptability of the tool under routine conditions.

The following are proposed as *specific objectives*:

- To develop, test and deploy the EMAeHealth tool [21], with the structure described above, capable of offering information, responses via algorithm, links to available resources, secure data archiving and monitoring, connection between various systems and the possibility of data analysis establishing continuous feedback and readjustment of the information presented. To provide it with content related to pregnancy, childbirth and postpartum.
- To evaluate the effectiveness of the tool in 1) improving health-related quality of life, 2) improving self-efficacy for labour and childbirth and self-efficacy in breastfeeding, and 3) reducing the number of visits to the obstetric emergency services and health centre in situations of "non-pathological pregnancy", "false labour pains" and "non-pathological puerperium".
- To evaluate the usability and acceptability of the tool by women and professionals, and any barriers to its implementation.
- To estimate the differential impact that EMAeHealth could have, based on sociodemographic and/or population characteristics.

Methods and analysis

This is an *Effectiveness-implementation hybrid design type I* [26], which combines 1) a multicentre, longitudinal, prospective, cluster randomised clinical trial to evaluate the effectiveness of EMAeHealth and 2) a mixed-method study to evaluate the usability and acceptability of the tool,

For peer review only - http://bmjopen.bmj.com/site/about/guidelines.xhtml

barriers and facilitators for its use and implementation in our health system. The mixed method study is made up of a qualitative study by focus groups (women, professionals and agents involved) and a quantitative analysis of implementation indicators. **(Fig. 1)**

For the effectiveness study, we have chosen a cluster randomised design to avoid, among other issues, contamination bias among women from the same centre, since they are likely to coincide, for example, in Maternal Education classes. In addition, as the intervention has been designed for shared decision-making, it did not seem appropriate for the same midwife to have women belonging to both control and intervention.

The study is being conducted between 2021 and 2023, with the first year dedicated to the development of the EMAeHealth digital tool. The general structure of the tool is described in the introduction. In the development of the prototype, the International Patient Decision Aid Standards (IPDAS) were applied, using a collaborative action research process, involving experts and patients, with qualitative research techniques, as well as focus groups, prioritisation and consensus techniques [21]. At this time, we are finalising content editing and software development and testing. We expect to start recruitment in July 2022. The participants will be followed-up for a year, the period of time that the intervention lasts. The final months of 2023 - first months of 2024 will be dedicated to the analysis and diffusion of results.

Regarding the *study population*, all Osakidetza-Basque Health Service public primary care centres that have midwives who are interested in optimising their clinical practice by adopting innovations will be eligible. In order to consider the *centre eligible*, the requirement will be that at least 50% of the midwives belonging to the centre have signed a collaboration agreement, as well as the approval of the Head of the Primary Care Centre (HPCC).

Osakidetza-Basque Health System is the public healthcare system of the Basque Country, a region located in the north of Spain. Osakidetza was created by the Health Department of the Basque Government in 1983. All the public hospitals and primary care centres of the Basque Region are

For peer review only - http://bmjopen.bmj.com/site/about/guidelines.xhtml

under this organisation, structured into several Integrated Health Organisations (IHO) spread throughout the Basque country. More than 30,000 professionals work for Osakidetza, and it could be considered the largest organisation in the Basque Country.

For the study of clinical effectiveness, an active surveillance system will be established to identify *eligible patients*, who are Spanish-speaking pregnant women attending their first appointment with the midwife between week 6 and 10 of gestation. Regarding their clinical profile, they are low-risk pregnant women. The midwives participating in the study will be in charge of recruiting them consecutively until they have the necessary sample from among the people using and not using the EMAeHealth tool. **(Fig. 2)**

Regarding the mixed-method study, to evaluate the quantitative implementation indicators, all pregnant women who have seen their midwives at the start of gestation at the health centres will be included for one year after the implementation of EMAeHealth. The same goes for the *qualitative* evaluation, through focus groups of women and professionals from the health centres *(model consent form as a supplementary file)*.

Random assignment of centres:

The eligible centres will be randomised centrally, blindly, and electronically, in the Biocruces-Bizkaia Research Institute, stratified by Integrated Healthcare Organisation (IHO) and in a 1:1 ratio. Therefore, the intervention allocated centres will have access to EMAeHealth, while it will not be accessible for the control group centres. Integrated Health Organisations are groups of health centres within the same geographical area, with similar characteristics and population, under the direction of the same hospital. Therefore, we assume that there are no pre-existing differences in the quality of care provided between centres that belong to the same integrated health organisation.

Intervention centres

For peer review only - http://bmjopen.bmj.com/site/about/guidelines.xhtml

All the patients in these centres, in the first consultation with the midwife, will be informed that EMAeHealth is available to them, and they will be told about the content of the tool. In addition, they will be invited to participate in the study of its clinical effectiveness. If the answer is affirmative, they must sign the consent form and will be provided with a link to the forms (baseline measurements and questionnaires for each measurement) to fill in electronically. Participants sign a consent form approved by the Ethics Committee and can leave the study whenever they want *(model consent form as a supplementary file).*

Control centres

Patients in these centres will not have EMAehealth. They will receive, like the intervention group, the usual care and the possibility of attending ME sessions, but not EMAeHealth. After being informed of the clinical effectiveness study, they will be asked for consent to participate *(see supplementary file).* Women who sign the consent form for participation in the study will be provided with the link to the forms for each measurement.

Outcomes

Access to EMAeHealth by women and professionals during the study period will be through access codes, which will allow the research team to track the use made by users of the tool while ensuring the confidentiality of the data. It also makes it possible, under safe conditions, to link this use with clinical variables from medical records and with the record of the forms filled in electronically at each measurement.

Results of clinical effectiveness: The primary outcome is change in Health-related quality of life, measured with the Spanish version of the SF-36 questionnaire [30]. The secondary variables are the number and reasons for visits to the obstetric emergency service and to the health centre for non-pathological pregnancy situations, and the labour stage at the time of going to the hospital for suspected labour onset (measurement is carried out using the Bishop test /dilation in cm, effacement and breaking of waters (YES/NO)) . As indirect indicators of autonomy in managing their own health

For peer review only - http://bmjopen.bmj.com/site/about/guidelines.xhtml

For peer review only

and well-being, self-efficacy for labour and childbirth and self-efficacy in breastfeeding will be measured using the Spanish version of Lowe's CBSEI scales [31] and the Breastfeeding Self-Efficacy Scale - Short Form [32], respectively.

Measurements of these variables will be performed at weeks 8 and 37 of pregnancy, and at weeks 1, 6, and 16 postpartum. Contact with the woman and the response to the questionnaires will be carried out electronically (via mobile phone).

Quality of life (QoL) is a parameter that has been defined and recently taken into account as a health indicator by WHO [33]. The concept of QoL could be defined as an individual's assessment of their own state in relation to their needs [33], so it seems appropriate that it should be the primary outcome measure. The Spanish version of the SF-36 questionnaire has been used to assess the health-related quality of life perceived by pregnant women in several studies [33; 34], showing good psychometric characteristics. The SF-36 questionnaire is made up of 8 domains, of which 4 are oriented towards physical aspects and another 4 towards mental aspects of health-related quality of life. In addition, two summaries are made of this physical and mental component, which explain between 80 and 85% of the variance of the 8 domains [30]. The scores of the questionnaire are transformed on a scale from 0 to 100 points; the higher the score, the better the perception of health-related quality of life. A difference of 5 points in the domain score, or between 2 and 3 points in the summary score is considered clinically relevant. [30]

Self-efficacy is one of the key factors influencing women's confidence and ability to cope with childbirth, and the Childbirth Self-Efficacy Inventory has been shown to be a valid and reliable instrument to measure maternal confidence in childbirth. The Spanish version of the Childbirth Self-Efficacy Inventory shows adequate psychometric properties (i.e. internal consistency and validity). Moreover, self-efficacy is a variable which is sensitive to external modifications, so we believe it may be suitable as one of the secondary measures to assess the effectiveness of using EMAeHealth.

For peer review only - http://bmjopen.bmj.com/site/about/guidelines.xhtml

Similarly, breastfeeding self-efficacy is a mother's confidence in her ability to breastfeed and is highly predictive of breastfeeding behaviours. The Spanish version of the BSES-SF can be considered a valid and reliable measure of maternal breastfeeding self-efficacy.

Indicators of the implementation process: Qualitative indicators: Usability and acceptability of the tool by patients and professionals, as well as barriers and facilitators for its use and adoption in the health system. The qualitative evaluation will be carried out through focus groups of women and professionals, between 6 and 8 per group and as many as necessary until the discourse is saturated [35]. A semi-structured script will be developed for the sessions, based on the theoretical constructs of the CFIR [28], which will also serve as the basis for the analysis. The CFIR includes 37 constructs within 5 main domains: intervention characteristics, outer setting, inner setting, individual characteristics and implementation process. Overall, the CFIR aims to help identify potential factors (i.e. barriers and facilitators) that are believed to influence implementation. Sessions will be audio-recorded and moderated by an expert in qualitative techniques while an assistant will take notes. Later the sessions will be transcribed and analysed. Quantitative indicators: adoption and frequency of the use of EMAeHealth and its areas according to stratification profiles and correlates with other variables (indicators: patients exposed to the digital tool, by centre and group; women with access to EMAeHealth who access the tool once; frequency of use of the tool, frequency of use of the different functions of the tool, etc.).

Predictive, modifying and confounding variables: *Patients*: Age, Parity, Nationality, Race, Social class, Educational level, Marital status, Work situation, Distance to hospital, Spontaneous pregnancy vs Assisted Reproductive technique, Obstetric history, and other clinical characteristics. *Professionals*: Sex, age, length of time in the position, type of contract, profile of adoption of innovations by professionals, measured by means of a questionnaire by Borracci et al., 2013. *Health centres*: reference IHOs, number of midwives and participation rate, rural or urban environment, and population served. **(Fig. 3)**

Statistical methods

For peer review only - http://bmjopen.bmj.com/site/about/guidelines.xhtml

EMAeHealth *Clinical Effectiveness Results*: quantitative comparisons will be made between intervention and control centres, with an intention-to-treat approach. Mixed models (linear or generalised as appropriate to the variable under study), which take into account the hierarchical structure of the data (patients grouped by midwives, midwives grouped by centre), will be used and 95% confidence intervals will be calculated. Likewise, per-protocol analysis will be carried out, in which the effectiveness of the express use of both the tool in general and in particular of some of its components will be evaluated, adjusting for possible confounding factors. Statistical analysis will be carried out by a professional who is not part of the research team.

Implementation process: For the *qualitative* evaluation of the tool and its implementation, the analysis of the focus groups, facilitated by the ATLAS.ti software programme, will be structured in three phases: a deductive analysis to determine which CFIR constructs influence its use in real life, followed by an inductive analysis to identify others not included in the CFIR framework that could influence its use, and a third validation phase using triangulation. The analysis results report will be provided to each patient and professional, who will have the opportunity to provide feedback to the team of analysts with their comments (“response validity”).

For the *quantitative* evaluation, the distribution of the indicators associated with the use and scope of the tool as well as its components will be described. Measures of central tendency, dispersion and proportions will be calculated, with 95% confidence intervals, and the relationship between the indicators and the predictor/confounding variables will be analyzed using mixed models.

Study size

For the clinical effectiveness study, 18 centres will need to recruit 30 patients each, in each arm of the study (N=1080). This will make it possible to detect a difference of 2 points as significant in the mental component of the SF-36 test, with an alpha error of less than 5% and power greater than 80%. A 10-point standard deviation is assumed for the mental SF-36 and an intra-cluster correlation of 0.01.

For peer review only - http://bmjopen.bmj.com/site/about/guidelines.xhtml

The calculation took into account the cluster design of the study and it was carried out using the National Institute of Health GRT sample size calculator* which assumes that the analysis will employ mixed-model regression methods.

* Research Methods Resources: National Institutes of Health. [Accessed 02/01/2020]. Available from: https://researchmethodsresources.nih.gov/grt-calculator

Patient involvement

In previous studies, multidisciplinary groups of midwives, nurses, paediatricians, epidemiologists, psychologists and pregnant and postpartum women were involved in the description and prioritisation of ME needs, as well as in the redesign of the ME and the design of an EMAeHealth tool to complement it [3, 6, 7, 8, 8, 15, 21]. In the present study, patients will be involved in testing the tool under construction, assessing its usability, acceptability and evaluating barriers and facilitators for its implementation in "real-world conditions". In addition, women will participate as study subjects in the evaluation of the effectiveness of the digital tool.

Ethics and dissemination

The study protocol has been reviewed and ethics approval obtained from the by the Clinical Research Ethics Committee of Euskadi, Spain in November 2020 (Ref: PI2020044).-This approval covers all centres included in the study. Participation in this study will be voluntary; individual written (or online) consent will be sought from all participants. Each participant will receive a study information sheet that outlines the project and expectations in plain language

Manuscripts will be published in open access journals and findings will be further disseminated through local and international conferences.

Acknowledgements

For peer review only - http://bmjopen.bmj.com/site/about/guidelines.xhtml

We would like to acknowledge the collaboration of those interested in the design of the EMAeHealth tool, the grant received by the Carlos III Health Institute in collaboration with the ISCII-Subdirectorate General for Evaluation and Promotion of Research (European Regional Development Fund-FEDER) and the support of Biocruces Bizkaia Health Research Institute. We also want to thank the invaluable work of Katy Ryan, who has been responsible for translating the manuscript into English.

References

1. Gagnon AJ, Sandall J. Individual or group antenatal education for childbirth or parenthood, or both. Cochrane Database of Systematic Reviews 2007, Issue 3. Art. No.: CD002869. DOI: 10.1002/14651858.CD002869.pub2.
2. https://www.nice.org.uk › Default › ***Antenatal*** Education Evidence Review Final.docx
3. Artieta-Pinedo I, Paz-Pascual C, Grandes G, et al. The benefits of antenatal education for the childbirth process in Spain. *Nurs Res*. 2010;59(3):194-202. doi:10.1097/NNR.0b013e3181dbbb4e.
4. Billingham K. Preparing for parenthood: the role of antenatal education. Community Practitioner : the Journal of the Community Practitioners' & Health Visitors' Association. 2011 May;84(5):36-38.
5. McMillan, Anita & Barlow, Jane & Redshaw, Margaret & Britain, Great. (2009). Birth and beyond: a review of the evidence about antenatal education. https://www.researchgate.net/publication/258369289_Birth_and_beyond_a_review_of_the_evidence_about_antenatal_education
6. Paz Pascual C, Artieta Pinedo I, Grandes G, Espinosa M, Gaminde Inda I, Payo Gordon J. [Perceived needs of women regarding maternity. Qualitative study to redesign maternal education]. Aten Primaria. 2016;48(10):657-664. doi:10.1016/j.aprim.2015.12.004
7. Artieta-Pinedo I, Paz-Pascual C, Grandes G, Espinosa M. Framework for the establishment of a feasible, tailored and effective perinatal education programme. *BMC Pregnancy Childbirth*. 2017;17(1):58. Published 2017 Feb 8. doi:10.1186/s12884-017-1234-7

For peer review only - http://bmjopen.bmj.com/site/about/guidelines.xhtml

8. Paz-Pascual C, Artieta-Pinedo I, Grandes G; ema.Q Group. Consensus on priorities in maternal education: results of Delphi and nominal group technique approaches. *BMC Pregnancy Childbirth*. 2019;19(1):264. Published 2019 Jul 24. doi:10.1186/s12884-019-2382-8

9. World Health Organization. Digital Health. https://www.who.int/ehealth/en/

10. Ruiz-Azarola A, Perestelo-Pérez L. Participación ciudadana en salud: formación y toma de decisiones compartida. Informe SESPAS 2012 [Citizens' participation in health: education and shared decision-making. SESPAS Report 2012]. *Gac Sanit*. 2012;26 Suppl 1:158-161. doi:10.1016/j.gaceta.2011.10.005

11. Mora J et al, (2019) Cómo avanzar con el paciente. Modelo afectivo efectivo. The Institute for Health and Strategy S.L. (Si-Health). https://www.sihealth.es/es/publicaciones-eventos/publicaciones/

12. Bert F, Gualano MR, Brusaferro S, et al. Pregnancy e-health: a multicenter Italian cross-sectional study on Internet use and decision-making among pregnant women. *J Epidemiol Community Health*. 2013;67(12):1013-1018. doi:10.1136/jech-2013-202584

13. Hughson JP, Daly JO, Woodward-Kron R, Hajek J, Story D. The Rise of Pregnancy Apps and the Implications for Culturally and Linguistically Diverse Women: Narrative Review. *JMIR Mhealth Uhealth*. 2018;6(11):e189. Published 2018 Nov 16. doi:10.2196/mhealth.9119

14. O'Higgins A, Murphy OC, Egan A, Mullaney L, Sheehan S, Turner MJ. The use of digital media by women using the maternity services in a developed country. Ir Med J. 2014 Nov-Dec;107(10):313-5. PMID: 25556255.

15. Artieta-Pinedo I, Paz-Pascual C, Grandes G, Villanueva G; Ema Q Group. An evaluation of Spanish and English on-line information sources regarding pregnancy, birth and the postnatal period. *Midwifery*. 2018;58:19-26. doi:10.1016/j.midw.2017.12.002

16. Wallwiener S, Müller M, Doster A, et al. Pregnancy eHealth and mHealth: user proportions and characteristics of pregnant women using Web-based information sources-a cross-

For peer review only - http://bmjopen.bmj.com/site/about/guidelines.xhtml

sectional study. *Arch Gynecol Obstet*. 2016;294(5):937-944. doi:10.1007/s00404-016-4093-y

17. Lupton D. The use and value of digital media for information about pregnancy and early motherhood: a focus group study. *BMC Pregnancy Childbirth*. 2016;16(1):171. Published 2016 Jul 19. doi:10.1186/s12884-016-0971-3
18. Rodger D, Skuse A, Wilmore M, et al. Pregnant women's use of information and communications technologies to access pregnancy-related health information in South Australia. *Aust J Prim Health*. 2013;19(4):308-312. doi:10.1071/PY13029
19. Brown HM, Bucher T, Collins CE, Rollo ME. A review of pregnancy iPhone apps assessing their quality, inclusion of behaviour change techniques, and nutrition information. *Matern Child Nutr*. 2019;15(3):e12768. doi:10.1111/mcn.12768
20. Kraschnewski JL, Chuang CH, Poole ES, et al. Paging "Dr. Google": does technology fill the gap created by the prenatal care visit structure? Qualitative focus group study with pregnant women. *J Med Internet Res*. 2014;16(6):e147. Published 2014 Jun 3. doi:10.2196/jmir.3385
21. Artieta-Pinedo I, Paz-Pascual C, Bully P, Espinosa M, EmaQ Group. Design of the Maternal Website EMAeHealth That Supports Decision-Making During Pregnancy and in the Postpartum Period: Collaborative Action Research Study.JMIR Form Res 2021; 5(8):e28855.URL: https://formative.jmir.org/2021/8/e28855. DOI: 10.2196/28855
22. Overdijkink SB, Velu AV, Rosman AN, van Beukering MD, Kok M, Steegers-Theunissen RP. The Usability and Effectiveness of Mobile Health Technology-Based Lifestyle and Medical Intervention Apps Supporting Health Care During Pregnancy: Systematic Review. *JMIR Mhealth Uhealth*. 2018;6(4):e109. Published 2018 Apr 24. doi:10.2196/mhealth.8834
23. Roch G, Borgès Da Silva R, de Montigny F, et al. Impacts of online and group perinatal education: a mixed methods study protocol for the optimization of perinatal health services. *BMC Health Serv Res*. 2018;18(1):382. Published 2018 May 29. doi:10.1186/s12913-018-3204-9

For peer review only

24. Berland GK, Elliott MN, Morales LS, et al. Health information on the Internet: accessibility, quality, and readability in English and Spanish. *JAMA*. 2001;285(20):2612-2621. doi:10.1001/jama.285.20.2612

25. Risling T, Martinez J, Young J, Thorp-Froslie N. Evaluating Patient Empowerment in Association With eHealth Technology: Scoping Review. *J Med Internet Res*. 2017;19(9):e329. Published 2017 Sep 29. doi:10.2196/jmir.7809

26. Leonardsen AL, Hardeland C, Helgesen AK, Grøndahl VA. Patient experiences with technology enabled care across healthcare settings- a systematic review. BMC Health Serv Res. 2020;20(1):779. Published 2020 Aug 24. doi:10.1186/s12913-020-05633-4

27. Curran GM, Bauer M, Mittman B, Pyne JM, Stetler C. Effectiveness-implementation hybrid designs: combining elements of clinical effectiveness and implementation research to enhance public health impact. Med Care. 2012;50(3):217-226. doi:10.1097/MLR.0b013e3182408812

28. Damschroder LJ, Aron DC, Keith RE, Kirsh SR, Alexander JA, Lowery JC. Fostering implementation of health services research findings into practice: a consolidated framework for advancing implementation science. Implement Sci. 2009;4:50. Published 2009 Aug 7. doi:10.1186/1748-5908-4-50

29. Gaglio B, Shoup JA, Glasgow RE. The RE-AIM framework: a systematic review of use over time. *Am J Public Health*. 2013;103(6):e38-e46. doi:10.2105/AJPH.2013.301299

30. Vilagut G, Ferrer M, Rajmil L, et al. El Cuestionario de Salud SF-36 español: una década de experiencia y nuevos desarrollos [The Spanish version of the Short Form 36 Health Survey: a decade of experience and new developments]. *Gac Sanit*. 2005;19(2):135-150. doi:10.1157/13074369

31. Cunqueiro MJ, Comeche MI, Docampo D. Childbirth Self-Efficacy Inventory: psychometric testing of the Spanish version. *J Adv Nurs*. 2009;65(12):2710-2718. doi:10.1111/j.1365-2648.2009.05161.x

For peer review only - http://bmjopen.bmj.com/site/about/guidelines.xhtml

32. Oliver-Roig A, d'Anglade-González ML, García-García B, Silva-Tubio JR, Richart-Martínez M, Dennis CL. The Spanish version of the Breastfeeding Self-Efficacy Scale-Short Form: reliability and validity assessment. *Int J Nurs Stud*. 2012;49(2):169-173. doi:10.1016/j.ijnurstu.2011.08.005

33. Martínez-Galiano JM, Hernández-Martínez A, Rodríguez-Almagro J, Delgado-Rodríguez M. Quality of Life of Women after Giving Birth: Associated Factors Related with the Birth Process. *J Clin Med*. 2019;8(3):324. Published 2019 Mar 7. doi:10.3390/jcm8030324

34. Schubert KO, Air T, Clark SR, et al. Trajectories of anxiety and health related quality of life during pregnancy. *PLoS One*. 2017;12(7):e0181149. Published 2017 Jul 24. doi:10.1371/journal.pone.0181149

35. Hennink MM, Kaiser BN, Weber MB. What Influences Saturation? Estimating Sample Sizes in Focus Group Research. *Qual Health Res*. 2019;29(10):1483-1496. doi:10.1177/1049732318821692

Footnotes

Author statement: IA, CP, PB and ME designed the EMAeHealth programme, with EMA-Q Group´s collaboration, AG has collaborated on the statistics and on the revision of the article. All of them have been involved in the design of the protocol and have collaborated in writing the manuscript; all authors read, contributed to, and finally approved the manuscript. *Contributors*: Arturo García (statistics) and patients involved.

Funding: This study has been funded by the Carlos III National Health Institute, (grant number: PI20/00899.), (Co-funded by European Regional Development Fund "A way to make Europe ").

Disclaimer: The funding source has not been involved in study design, collection, analysis and interpretation of data, writing of the report or in the decision to submit the article for publication.

Conflicts of interests: The authors declare that they have no competing interests.

For peer review only - http://bmjopen.bmj.com/site/about/guidelines.xhtml

Patient involvement: See details in the methods and analysis section.

Patient consent for publication: Not required

Ethics approval: Clinical Research Ethics Committee of Euskadi (Spain), in November 2020 (Ref: PI2020044)

Provenance and peer review: Not commissioned; externally peer reviewed

Data sharing statement: Access to the data is feasible by requesting permission from the main researcher, Isabel Artieta. The protocol and the research results will be published. Access to data, ICF, etc. are available by requesting it from the main researcher, Isabel Artieta. (MARIAISABEL.ARTIETAPINEDO@osakidetza.eus)

Word count: 4603 (References and footnotes not included), 5903 (Total)

Figure legends

Figure 1. EMAeHealth design description

Figure 2. EMAeHealth effectiveness flow chart

Figure 3. SPIRIT-Timetable

For peer review only - http://bmjopen.bmj.com/site/about/guidelines.xhtml

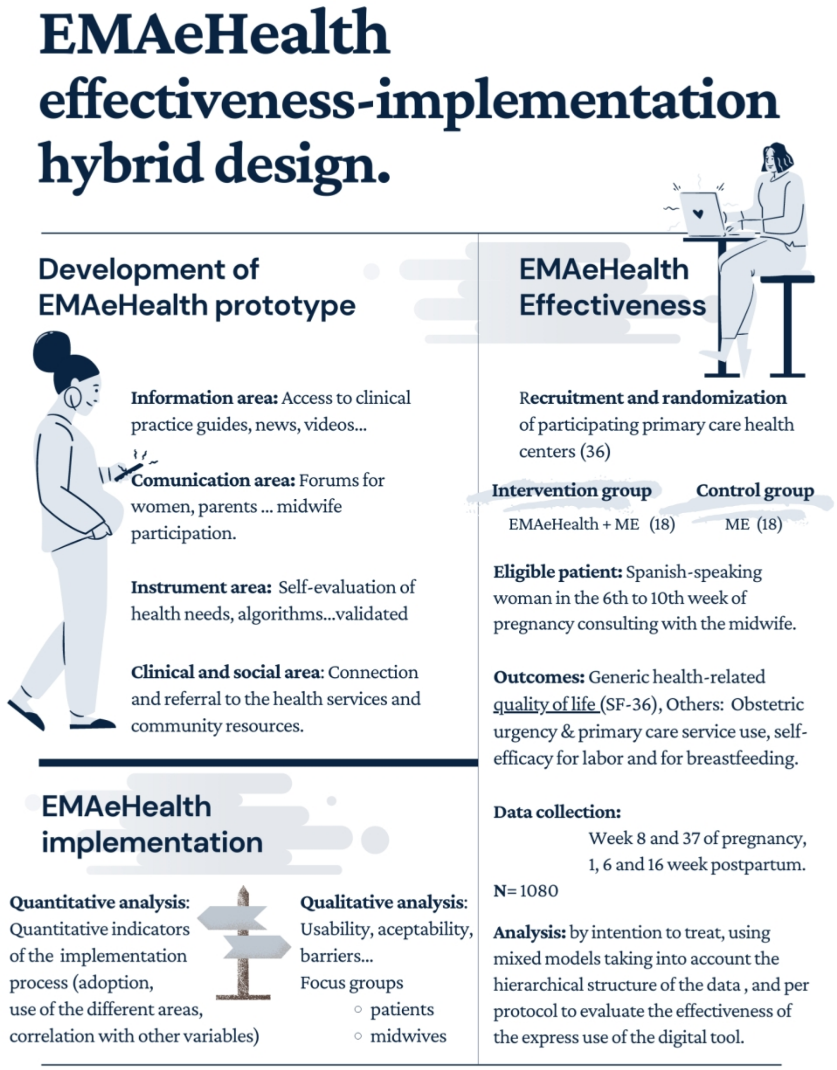

Figure 1. EMAeHealth design description

1060x1500mm (38 x 38 DPI)

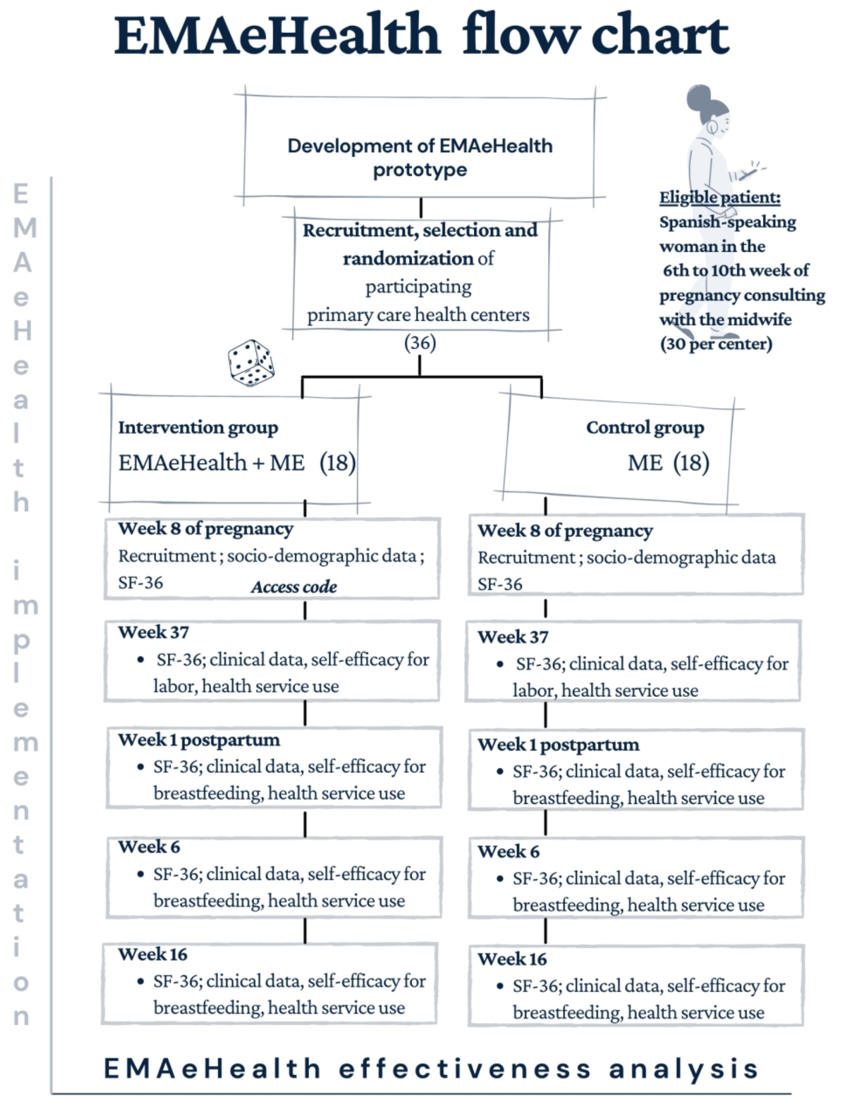

Figure 2. EMAeHealth effectiveness flow chart

1060x1500mm (38 x 38 DPI)

EMAeHealth Spirit timeline table.

	STUDY PERIOD							
	Enrolment	Allocation	Post-allocation					Close-out
TIMEPOINT**	*January 2022-june 2022*	0	*week 8 of pregnancy*	*week 37 of pregnancy*	*Week 1 postpartum*	*Week 6 postpartum*	*Week 16 postpartum*	*June 2023-Dec 2023*
**ENROLMENT:**	January 2022-june 2022. Enrollment of participants will be after allocation of the centers. It will be done, for both arms, consecutively, until complete the sample (Total N=1080)							
**Eligibility screen**	Spanish-speaking woman around the 8th week of pregnancy consulting with the midwife of eligible centers.							
**Informed consent**	First consultation with the midwife between week around 8th week of gestation							
**Allocation**		The eligible health centers will be randomized centrally, blindly, and electronically, in Biocruces-Bizkaia Research Institute, stratified by Integrated Healthcare Organization (IHO) and in a 1: 1 ratio. Therefore, the participating centers will have access to EMAeHealth, while it will not be accessible to the control group centers.						
**INTERVENTIONS:**								
EMAeHealth +traditional ME (usual care)			←	—	—	—	→	
traditional ME (usual care)			←	—	—	—	→	
**ASSESSMENTS:**								
Baseline: Socio-demographic data and SF-36			X					
Outcomes: SF-36, clinical outcomes, self-efficacy for labor, self-efficacy for breastfeeding, health service use				X	X	X	X	X
Predictive, modifying and confounding variables			X	X	X	X	X	X

For peer review only

Patient information sheet for clinical trial and consent

Patient Information Sheet

For years we have been trying to improve the initiatives we carry out at Osakidetza to improve care for women during pregnancy and postpartum. We are therefore contacting you to ask for your consent to participate in a study your health center and midwife are collaborating in.

Invitation to participate and description of the procedure

Your health center and particularly your midwife are actively collaborating with the Bizkaia Primary Care Research Unit, part of the Biocruces-Bizkaia institute, to promote maternal education that is better adapted to the needs of women today. As a result, our research team is developing a website to support Maternal Education and we want to know how effective it is, so we would like to ask for your participation.

This website will offer rigorous information, selected by health professionals, on topics of interest throughout the period of pregnancy, childbirth and postpartum. It will enable women to access their clinical data securely, and will contain and offer resources at key moments in the maternity/paternity process. It will inform and support women in leading a healthy lifestyle, helping in the development of a birth plan that is adapted to each woman, advising on the appropriate choice at the onset of childbirth and offering care in the first days postpartum.

The website will be available to some randomly selected health centers, while others will not have this resource. We are doing this to compare the results on the health of women who use this resource (which you access with a password provided by your midwife) with those of women who do not have access to it.

Your participation in the study, regardless of your reference health center (with or without the website) will consist of filling in a series of sociodemographic questionnaires (e.g. age, level of studies, profession) and clinical questions (such as quality of life related to health or perception of self-efficacy during the birth process and breastfeeding), and it will also involve giving us permission to collect data from your medical records related to your pregnancy, childbirth and postpartum history.

The questionnaires must be completed at the beginning of pregnancy (approximately week 8), at weeks 24 and 34-36, and at weeks 1 and 6 postpartum. You fill it in online; your midwife will ask you for your mobile phone number and/or email address, and you will be sent a link to access the questionnaires. You will be able to complete them on a mobile phone, tablet or computer.

If you decide that you wish to participate, we will ask you to sign the attached consent form, which will be filed with the rest of the study documents.

Data processing and protection

The data that we collect via your mobile phone will be processed in an anonymized form, since it will be accessed with a numerical code, and confidentiality will be guaranteed at all times. The data collected for the study will be identified by this code, so that it does not include information that can identify you, and only your study doctor/researchers will be able to relate this data to you and your medical records. Therefore, your identity will not be disclosed to any person,

For peer review only

except in cases of medical emergency or legal requirement. The processing, communication and transfer of personal data of all participants will comply with the provisions of the law.

This study complies with the provisions of REGULATION (EU) 2016/679 OF THE EUROPEAN PARLIAMENT AND OF THE COUNCIL of 27 April 2016 on the protection of natural persons with regard to the processing of personal data and the free movement of such data, and of Organic Law 3/2018, of 5 December 2018 on the Protection of Personal Data and guarantee of digital rights that repeals Organic Law 15/1999 of 5 December 1999, on the protection of personal data. Personal data will be processed by the Basque Health Service, Osakidetza. No data will be transferred to third parties, except under legal obligation. The patient will be informed that they have the right of access, rectification, deletion of their data, and limitation or opposition to its use. In addition, further information on data protection can be found at the following website: http://www.osakidetza.euskadi.eus/protecciondatos

Benefits and risks

Participation in the study is entirely altruistic and carries no risk to you. The only cost that can be incurred is the time dedicated to answering the questions in the questionnaires. You are not required to answer questions that you consider to be private.

In return, you will be collaborating with research dedicated to the evaluation and improvement of the health service.

Voluntary participation

Your participation in the study is entirely voluntary.

Right to withdraw from the study

You have the right to withdraw from the study at any time without consequence to you.

Who to contact about the study

You can contact the Lead Researcher, Isabel Artieta, on tel. 946007711 if you need any clarification or want to resolve any doubts regarding the study.

The Coordinating Center for the study is the Bizkaia Primary Care Research Unit, which is part of Biocruces-Bizkaia and is located in Building 3, C/Plaza de Cruces, s/n. 48903 Barakaldo (Bizkaia). Tel. 946006637.

This study has been supervised and approved by the Basque Clinical Research Ethics Committee. (CEIm)

DECLARATION OF CONSENT

Study Title: “**Effectiveness and usability of a website for the self-management of women's health needs during pregnancy, childbirth and the postpartum period: EMAeHealth**”

Centre: **Bizkaia Primary Care Research Unit (Biocruces-Bizkaia Institute)**

For peer review only

I (name and surname) declare that

……

I have read the information sheet given to me.

I was able to ask questions about the study.

I have received enough information about the study.

I understand that in case of doubt I can go to the lead researcher:

Isabel Artieta. Zuazo Health Center (Barakaldo). Tel. 946007711

I understand that my participation is voluntary and that at any time, without explanation and without any impact on my medical care, I can withdraw from the study.

I freely give my consent to participate in the study.

DATE: PARTICIPANT'S SIGNATURE

The data collected will be added to a computer database in anonymized form, and be used to evaluate the research. This study complies with the provisions of REGULATION (EU) 2016/679 OF THE EUROPEAN PARLIAMENT AND OF THE COUNCIL of 27 April 2016 on the protection of natural persons with regard to the processing of personal data and the free movement of such data, and of Organic Law 3/2018, of 5 December 2018 on the Protection of Personal Data and guarantee of digital rights that repeals Organic Law 15/1999 of 5 December 1999, on the protection of personal data. Personal data will be processed by the Basque Health Service, Osakidetza. No data will be transferred to third parties, except under legal obligation. The patient will be informed that they have the right of access, rectification, deletion of their data, and limitation or opposition to its use. In addition, further information on data protection can be found at the following website: http://www.osakidetza.euskadi.eus/protecciondatos

THIS STUDY HAS BEEN EVALUATED AND APPROVED BY THE BASQUE CLINICAL RESEARCH ETHICS COMMITTEE (CEIm)

For peer review only

Focus groups of patients and focus groups of professionals and consent

Patient Information Sheet

In Osakidetza, we are trying to improve care for women during pregnancy and postpartum, and we would like your participation in a focus group in order to **get your opinion about the EMAeHealth website** that we have developed.

Invitation to participate in a focus group and description of the procedure

Your health center and particularly your midwife, together with researchers from the Bizkaia Primary Care Research Unit (Biocruces-Bizkaia), are looking into how to promote care that is better adapted to the needs of women today. To achieve this goal, as you know, a website called EMAeHealth has been designed.

As we would like to get a better idea of the opinions of the users about this new resource for Maternal Education - in terms of its ease of use, appropriateness and effectiveness - we would like to invite you to attend a focus group to explore these issues in depth. This participation is voluntary and only means attending a focus group meeting once. This meeting will be held on xxxxx (date), at xxxx (address) and will last approximately 90 minutes. The discussion will be moderated by two researchers from the Research Unit (xx and xx) and will be attended by other web users (between 6 and 8 of them) from different health centers, to talk about the topics under discussion.

Data processing and protection

The conversation generated by the group will be recorded entirely in audio to facilitate its subsequent transcription and analysis, with the collaboration of the qualitative studies research company xxx, which complies with the relevant legislation on data protection and is based in the Basque Country. We assure you that your opinions will be anonymized and that the confidentiality of the data collected will be guaranteed at all times. The audio files of the recordings and their corresponding transcripts (with names previously removed) will be stored in the computers of the Research Unit, access to which is restricted to researchers and

For peer review only

password-protected. Before the start of the recording, each participant will sign a consent form to authorize their participation in the study.

This study complies with the provisions of REGULATION (EU) 2016/679 OF THE EUROPEAN PARLIAMENT AND OF THE COUNCIL of 27 April 2016 on the protection of natural persons with regard to the processing of personal data and the free movement of such data, and of Organic Law 3/2018, of 5 December 2018 on the Protection of Personal Data and guarantee of digital rights that repeals Organic Law 15/1999 of 5 December 1999, on the protection of personal data. Personal data will be processed by the Basque Health Service, Osakidetza. No data will be transferred to third parties, except under legal obligation. The patient will be informed that they have the right of access, rectification, deletion of their data, and limitation or opposition to its use. In addition, further information on data protection can be found at the following website: http://www.osakidetza.euskadi.eus/protecciondatos

Benefits and risks

During the focus group discussion, the moderators will explain in detail what the technique involves, and you will be able to ask any questions or raise doubts throughout the entire session to make sure that you feel comfortable in that situation. You will also be given all kinds of opportunities to express your opinions freely and voluntarily, guaranteeing you an atmosphere of respect and confidentiality at all times. The main benefit of this participation is sharing your experience about the website, reflecting on ways to improve it and adapt it to the objective of promoting Maternal Education to meet your needs, thus contributing to the improvement of our health system.

Moreover, the moderators will take measures to minimize the impact of any potential risk that you or other participants may perceive or feel during the discussion in the group (such as fatigue, discomfort with any questions or conflict between participants). For example, you can take breaks, remain silent, state that you wish not to continue in the study, request more information about the objectives and procedures of the study and so on.

We remain at your entire disposal to offer any clarifications and answer your questions. If you need more details, you can contact the lead researcher of the study or the Bizkaia Primary Care Research Unit:

- **Isabel Artieta. Zuazo Health Center (Barakaldo). Tel. 946007711**
- **Bizkaia Primary Care Research Unit (Biocruces-Bizkaia). Tel. 946006637.** (Maite Espinosa)

For peer review only

Healthcare Professional Information Sheet

For years we have been involved in improving the initiatives we carry out in Osakidetza to improve care for women during pregnancy and postpartum. Therefore, we are contacting you to request your participation in a focus group, in order to **get your opinion about the EMAeHealth website** that we are developing.

Invitation to participate in a focus group and description of the procedure

We work with the Bizkaia Primary Care Research Unit (UIAPB) - Biocruces Bizkaia, in the development of strategies to promote Maternal Education that is better adapted to the needs of women today. To achieve this goal, as you know a website, EMAeHealth, has been designed, which we intend to subject to a constant process of improvement and updating.

As we would like to hear the opinions of the professionals about this new resource for supporting maternal education - in terms of its ease of use, suitability and effectiveness - we would like to invite you to attend a group conversation, or focus group, to analyze these issues in depth. Participation is voluntary and only requires you to attend once. This meeting will be held on xxxxx (date), at xxxx (address) and will last approximately 90 minutes. The conversation will be moderated by two researchers from the Research Unit (xx and xx) and will be attended by other professional users of the web (between 6 and 8 people), from different health centers, to talk about the topics under discussion.

Data processing and protection

Following the methodology of a focus group, the conversation generated by the group will be recorded entirely in audio to facilitate its subsequent transcription and analysis. In this task, the qualitative studies company xxx will collaborate with the UIAPB researchers. The company XXX (description) meets all the applicable requirements in terms of quality and data protection, and is based in the Basque Country, at (address xxx). The opinions of the participants will be processed in anonymized form and the confidentiality of the data collected will be guaranteed at all times. The audio files of the recordings and their corresponding transcripts (previously anonymized) will be stored on the computers of the Research Unit,

For peer review only

access to which is restricted to researchers and password-protected. Before the start of the recording, each participant will sign a consent form to authorize their participation in the study.

This study complies with the provisions of REGULATION (EU) 2016/679 OF THE EUROPEAN PARLIAMENT AND OF THE COUNCIL of 27 April 2016 on the protection of natural persons with regard to the processing of personal data and the free movement of such data, and of Organic Law 3/2018, of 5 December 2018 on the Protection of Personal Data and guarantee of digital rights that repeals Organic Law 15/1999 of 5 December 1999, on the protection of personal data. Personal data will be processed by the Basque Health Service, Osakidetza. No data will be transferred to third parties, except under legal obligation. The patient will be informed that they have the right of access, rectification, deletion of their data, and limitation or opposition to its use. In addition, further information on data protection can be found at the following website: http://www.osakidetza.euskadi.eus/protecciondatos

Benefits and risks

At the beginning of the session, the moderators will explain in detail what the technique involves and you will be able to clarify any doubts about the interview. Likewise, the participants will be able to express their opinions freely, in a climate of respect and confidentiality. Participation in the group is entirely altruistic, the main benefit being the possibility of sharing your experience about the website with other colleagues, reflecting on ways to improve it and adapt it to the objective of promoting Maternal Education that is more appropriate to the needs of women today.

Moreover, the moderators will take measures to minimize the impact of any potential risk that you may perceive during the development of the focus group (such as fatigue, discomfort with any questions or conflict between participants). You can withdraw your participation if you wish at any time, even during the interview itself. We remain at your entire disposal to offer any clarification or to answer your questions. If you need more information, you can contact the lead researcher of the study or the Bizkaia Primary Care Research Unit:

- **Isabel Artieta. Zuazo Health Center (Barakaldo). Tel. 946007711**
- **Bizkaia Primary Care Research Unit (Biocruces-Bizkaia). Tel. 946006637.** (Maite Espinosa)

Thank you very much.

For peer review only

DECLARATION OF CONSENT

Study Title: "**Effectiveness and usability of a website for the self-management of women's health needs during pregnancy, childbirth and the postpartum period: EMAeHealth**"

Centre: **Bizkaia Primary Care Research Unit (Biocruces-Bizkaia Institute)**

I (name and surname) declare that

....

I have read the information sheet given to me.
I was able to ask questions about the study.
I have received enough information about the study.
I know that in case of doubt I can go to the lead researcher:

Isabel Artieta. Zuazo Health Center (Barakaldo). Phone 946007711

I understand that my participation in the focus group is voluntary and that I can leave the meeting at any time, without explanation and without repercussion.
I freely give my consent for the meeting to be audio recorded and for the content of the meeting, including my contributions, to be transcribed in anonymized form for later analysis and research purposes.

DATE: PARTICIPANT'S SIGNATURE.

The data collected will be added to a computer database in anonymized form, and be used to evaluate the research. This study complies with the provisions of REGULATION (EU) 2016/679 OF THE EUROPEAN PARLIAMENT AND OF THE COUNCIL of 27 April 2016 on the protection of natural persons with regard to the processing of personal data and the free movement of such data, and of Organic Law 3/2018, of 5 December 2018 on the Protection of Personal Data and guarantee of digital rights that repeals Organic Law 15/1999 of 5 December 1999, on the protection of personal data. Personal data will be processed by the Basque Health Service, Osakidetza. No data will be transferred to third parties, except under legal obligation. The patient will be informed that they have the right of access, rectification, deletion of their data, and limitation or opposition to its use. In addition, further information on data protection can be found at the following website: http://www.osakidetza.euskadi.eus/protecciondatos

THIS STUDY HAS BEEN EVALUATED AND APPROVED BY THE BASQUE CLINICAL RESEARCH ETHICS COMMITTEE

For peer review only

SPIRIT

STANDARD PROTOCOL ITEMS: RECOMMENDATIONS FOR INTERVENTIONAL TRIALS

SPIRIT 2013 Checklist: Recommended items to address in a clinical trial protocol and related documents*

Section/item	Item No	Description
**Administrative information**		
Title	1	**EMAeHealth, digital tool for the self-management of women's health needs during pregnancy, childbirth and the puerperium. Study protocol for an effectiveness-implementation hybrid design**. (Title page. Pag.1)
Trial registration	2a	ClinicalTrials.gov ID: NCT04937049
	2b	
Protocol version	3	Manuscript BMJ- 24-06-2021-v.1_EN.docx
Funding	4	Footnotes section. Funding section. Page 19.
Roles and responsibilities	5a	Footnotes section. Page 19 (Contributors)
	5b	Footnotes section. Page 19 (Funding)
	5c	Footnotes section. Page 19 (Disclaimer)
	5d	Footnotes section. Page 19
**Introduction**		
Background and rationale	6a	Introduction section. Page 3, 4, 5 and 6.
	6b	Methods and analysis section. Page 9 (Random assignment of the centers)
Objectives	7	Aims section. Pages 6 and 7
Trial design	8	Methods and analysis section. Page 7,8,9
**Methods: Participants, interventions, and outcomes**		
Study setting	9	Methods section. Pages 8 and 9.
Eligibility criteria	10	Methods section. Pages 8 (eligible centers) and page 9 (eligible patients)

For peer review only - http://bmjopen.bmj.com/site/about/guidelines.xhtml

For peer review only

Interventions	11a	Background page 5 (last paragraph) Methods section, page 9 (Intervention centers)
	11b	N/A
	11c	N/A
	11d	N/A
Outcomes	12	Methods section. Outcomes (pages 10,11,12)
Participant timeline	13	Methods section. Outcomes (page11) and figure 2
Sample size	14	Methods section. Statistical methods: Study size page 13.
Recruitment	15	Methods section. Figure 1 and pages 8, and 9.
**Methods: Assignment of interventions (for controlled trials)**		
Allocation:		
Sequence generation	16a	Methods section. *Random assignment of centers* (page 9)
Allocation concealment mechanism	16b	Methods section. *Random assignment of centers* (pages 9)
Implementation	16c	Methods section. Random assignment of centers (pages 9), *Participating centers*)
Blinding (masking)	17a	Methods section. Statistical methods: EMAeHealth *Clinical Effectiveness Results* page 12 (paragraph 1)
	17b	N/A
**Methods: Data collection, management, and analysis**		
Data collection methods	18a	Methods section. Outcomes (page 10, paragraph 1,2 and 3)
	18b	N/A
Data management	19	Methods section. Outcomes (pages 10)
Statistical methods	20a	Methods section. Statistical methods (pages 12)
	20b	Methods section. Statistical methods (pages 12)
	20c	Methods section. Statistical methods (pages 12)
**Methods: Monitoring**		

Data monitoring	21a	The research team will oversee and manage the project. A Trial Monitoring Committee has been established and contains a representative group of the researchers (Midwifes, Quality management expert, statistic, psychologist) who are responsible for the conduct of the trial and monitoring the study. There is not competing interest.
	21b	The trial Monitoring Committee will contact the midwives every fortnight to promote capturing the sample and gradually obtaining information on the intervention process. Bimonthly reports will be prepared on progress. Biannual meetings will be held for quality control and ongoing training.
Harms	22	N/A
Auditing	23	Annual reports will be prepared for the ethics committee, the Biocruces-Bizkaia clinical research institute and the Carlos III Health Institute
**Ethics and dissemination**		
Research ethics approval	24	See Ethics approval and consent to participate in Footnotes page 19.
Protocol amendments	25	No important protocol modifications are foreseen because because this is the final version of the protocol approved by the corresponding Ethics committee.
Consent or assent	26a	Methods section page 9 and 10
	26b	N/A
Confidentiality	27	Methods/ Outcomes 1st paragraph.
Declaration of interests	28	Footnotes section: Competing interest page 19.
Access to data	29	The study is ongoing. The datasets that we will use and/or analysed during the study will be available from the corresponding author on reasonable request.
Ancillary and post-trial care	30	N/A
Dissemination policy	31a	The results of the study will be published in open access scientific journals and scientific and professional conferences and meetings. Patient associations will also be informed.

For peer review only

	31b	We will follow the recommendations of ICMJE Authorship eligibility guidelines.
	31c	N/A
**Appendices**		
Informed consent materials	32	Model consent form and other related documentation given to participants and authorised surrogates are available as a supplementary file
Biological specimens	33	N/A

*It is strongly recommended that this checklist be read in conjunction with the SPIRIT 2013 Explanation & Elaboration for important clarification on the items. Amendments to the protocol should be tracked and dated. The SPIRIT checklist is copyrighted by the SPIRIT Group under the Creative Commons "Attribution-NonCommercial-NoDerivs 3.0 Unported" license.

For peer review only
